# Experimental and Genomic Evaluation of the Oestrogen Degrading Bacterium *Rhodococcus equi* ATCC13557

**DOI:** 10.3389/fmicb.2021.670928

**Published:** 2021-07-01

**Authors:** Sarah L. Harthern-Flint, Jan Dolfing, Wojciech Mrozik, Paola Meynet, Lucy E. Eland, Martin Sim, Russell J. Davenport

**Affiliations:** ^1^School of Engineering, Newcastle University, Newcastle upon Tyne, United Kingdom; ^2^Faculty Engineering and Environment, Northumbria University, Newcastle upon Tyne, United Kingdom; ^3^Department of Inorganic Chemistry, Faculty of Pharmacy, Medical University of Gdańsk, Gdańsk, Poland; ^4^School of Computing Science, Newcastle University, Newcastle upon Tyne, United Kingdom

**Keywords:** *Rhodococcus equi*, oestrogen, genome, degradation, genes, bacteria

## Abstract

*Rhodococcus equi* ATCC13557 was selected as a model organism to study oestrogen degradation based on its previous ability to degrade 17α-ethinylestradiol (EE2). Biodegradation experiments revealed that *R. equi* ATCC13557 was unable to metabolise EE2. However, it was able to metabolise E2 with the major metabolite being E1 with no further degradation of E1. However, the conversion of E2 into E1 was incomplete, with 11.2 and 50.6% of E2 degraded in mixed (E1-E2-EE2) and E2-only conditions, respectively. Therefore, the metabolic pathway of E2 degradation by *R. equi* ATCC13557 may have two possible pathways. The genome of *R. equi* ATCC13557 was sequenced, assembled, and mapped for the first time. The genome analysis allowed the identification of genes possibly responsible for the observed biodegradation characteristics of *R. equi* ATCC13557. Several genes within *R. equi* ATCC13557 are similar, but not identical in sequence, to those identified within the genomes of other oestrogen degrading bacteria, including *Pseudomonas putida* strain SJTE-1 and *Sphingomonas* strain KC8. Homologous gene sequences coding for enzymes potentially involved in oestrogen degradation, most commonly a cytochrome P450 monooxygenase (*oecB*), extradiol dioxygenase (*oecC*), and 17β-hydroxysteroid dehydrogenase (*oecA*), were identified within the genome of *R. equi* ATCC13557. These searches also revealed a gene cluster potentially coding for enzymes involved in steroid/oestrogen degradation; 3-carboxyethylcatechol 2,3-dioxygenase, 2-hydroxymuconic semialdehyde hydrolase, 3-alpha-(or 20-beta)-hydroxysteroid dehydrogenase, 3-(3-hydroxy-phenyl)propionate hydroxylase, cytochrome P450 monooxygenase, and 3-oxosteroid 1-dehydrogenase. Further, the searches revealed steroid hormone metabolism gene clusters from the 9, 10-*seco* pathway, therefore *R. equi* ATCC13557 also has the potential to metabolise other steroid hormones such as cholesterol.

## Introduction

Since the 1990s, there have been increasing concerns about concentrations of endocrine-disrupting (ED) steroidal oestrogens in the aquatic environment and their effects upon aquatic organisms (Sumpter and Jobling, 1995; Jobling et al., 2002). Of particular relevance are the oestrogens; oestrone (E1), 17β-oestradiol (E2), and oestriol (E3), which are naturally occurring steroid hormones derived from cholesterol and released from the ovaries, adrenal cortex, testes, adipose tissue, other non-reproductive tissues, and the placenta in humans (Shutt et al., 1974; Hanukoglu, 1992; Simpson et al., 1994; Nelson and Bulun, 2001; Simoni et al., 2002; Cui et al., 2013). The biosynthesis of oestrogens is also present in animals including mammals (Andaluri et al., 2012; Ray et al., 2013). Additionally, the synthetic oestrogen 17α-ethinylestradiol (EE2), the main ingredient of some contraceptive pharmaceuticals, is of concern due to its environmental persistence and harmful effect on living organisms (Aris et al., 2014). Therefore, EE2, E2, and E1 have been included in the first watch list of substances for European Union-wide monitoring (European Commission, 2018).

Oestrogen degrading bacteria have been isolated from various environments, where oestrogen degradation has been associated with growth-linked metabolism (Supplementary Tables 1, 2). Aerobic oestrogen degradation has been proposed to proceed *via* the 4, 5-*seco* pathway (Chen et al., 2017, 2018). Alternative degradation pathways of oestrogen have also been proposed, such that a singular complete metabolic pathway of oestrogen degradation by bacteria remains unclear, especially in comparison to the well-characterised metabolic pathway of testosterone, an analogous steroid hormone, by *Comamonas testosteroni* (Horinouchi et al., 2012). Until recently, studies proposing oestrogen degradation pathways by bacteria have mainly relied on metabolite identification through chemical analysis only (Lee and Liu, 2002; Xuan et al., 2008; Kurisu et al., 2010; Nakai et al., 2011). This has allowed the prediction of the putative enzymes that may carry out each step. However, given the uncertainty of the proposed pathways, it is important to evaluate the genomes of oestrogen degrading bacteria to identify the genes coding for potential enzymes involved in the metabolism of oestrogen.

Genomic analysis has been critical in identifying genes coding for enzymes in steroid degradation pathways and understanding their evolution. This approach has been successful in discovering the testosterone degradation pathway in *C. testosteroni* (Horinouchi et al., 2012). More recently, 8000 taxa were predicted to degrade steroids by searching genome databases for gene clusters coding enzymes involved in steroid degradation (Bergstrand et al., 2016). The cholate degradation pathway was found to be evolutionarily conserved, with genes encoding enzymes within cholate and cholesterol degradation pathways located on a single gene cluster within the genome of *Rhodococcus equi* (Bergstrand et al., 2016). The whole genomes of 16 oestrogen degrading bacteria have been sequenced in the past decade, all but one of which are aerobes (see Supplementary Table 3). This includes; four E2-only degraders; one E1-only degrader; seven E1 and E2-degraders; one degrader of E1, E2, and E3; and one degrader of E1, E2, E3, and EE2 (*Pseudomonas citronellolis* SJTE-3, Zheng et al., 2016). The denitrifying anaerobe, *Denitratisoma* sp. strain DHT3, can degrade E2 into androgens in the presence of vitamin B12 (Wang P.H. et al., 2020).

The first oestrogen-degrader genome to be sequenced was that of *Sphingomonas* strain KC8 (Hu et al., 2011). Several genes encoding the enzymes suggested to be putatively involved in oestrogen degradation, such as hydroxysteroid dehydrogenase, 3-ketosteroid-delta1-dehydrogenase, Rieske dioxygenase, catechol 2,3-dioxygenase, were observed within its genome (Hu et al., 2011), as well as that of *Pseudomonas putida* strain SJTE-1 (Liang et al., 2012). Putative genes for enzymes involved in the transformation of E2 to E1 and its subsequent degradation have been further identified (Chen et al., 2017). One gene, *oecA*, encoding 17β-hydroxysteroid dehydrogenase was not clustered with other genes but was expressed in the presence of E2 relative to a control, demonstrating the conversion of E2 to E1 in heterologous expression analyses (Chen et al., 2017). Two other genes on two gene clusters were also expressed in the presence of E2 relative to a control, these include; *oecB* that encodes flavin-dependent monooxygenase in cluster I and *oecC* that encodes an extradiol dioxygenase in cluster II (both involved in E1 A-ring degradation *via* 4-hydroxyestrone and the 4, 5-*seco* pathway). Other genes were also identified as coding for β-oxidation in cluster III, putatively involved in the degradation of steroid C/D rings (Chen et al., 2017; Hsiao et al., 2020, 2021). Further gene and metabolite analysis are indicative that these clusters are involved in the degradation of oestrogens to 3aα-H-4α(3′-propanoate)- 7aβ-methylhexahydro-1,5-indanedione (HIP), a common steroid metabolite that undergoes further degradation through a central pathway (Wu et al., 2019). There is growing evidence that these gene clusters are evolutionarily conserved within the genomes of putative oestrogen degraders. Indeed, clusters similar to clusters I and II within the genomes of *Novosphingobium tardaugens* and *Altererythrobacter estronivorus*, and homologous open reading frames (ORFs) within cluster III were identified within the genomes of *C. testosteroni, A. estronivorus*, and *N. tardaugens* (Chen et al., 2017; Ibero et al., 2020). Additionally, those genes involved in the 4, 5-*seco* pathway (*oecB* and *oecC*) were identified in the genomes of 12 bacteria belonging to the alphaproteobacteria or gammaproteobacteria (>40% amino acid sequence identity) (Chen et al., 2017).

More recent studies have confirmed both the existence of orthologues for the above clusters and the genes on them, in addition to their function (through gene knock-out experiments) or upregulation in transcriptomic studies. Thus, orthologues of 17β-hydroxysteroid dehydrogenase (*oecA*), which encodes an enzyme that transforms E2 to E1, have been identified in *N. tardaugens* NBRC16725 (Ibero et al., 2020), *Deinococcus actinosclerus* SJTR1 (Xiong et al., 2018), and the actinobacteria, *Rhodococcus* sp. strain DSSKP-R-001 (Zhao et al., 2018; Tian et al., 2020) and *Rhodococcus* sp. P14 (Ye et al., 2017), which were shown to be upregulated in the presence of E2 in the latter two organisms. Homologous clusters to gene clusters I, II, and III, and specific orthologues of *oecB* and *oecC* involved in the degradation of E1, have been identified with confirmed function for the latter two enzymes in *N. tardaugens* NBRC16725 (Ibero et al., 2020) and *Rhodococcus* sp. strain B50 (Hsiao et al., 2020). In proteobacteria, *N. tardaugens* NBRC16725, the *edc* gene cluster was determined functionally essential to the degradation of E1 (Ibero et al., 2020). The *edc* cluster contains, gene *edcA* encoding cytochrome P450 hydroxylase, gene *edcB* encoding 4-hydroxyestrone-4,5-dioxygenase, gene *edcC* encoding a member of the indolepyruvate ferredoxin oxidoreductase family, gene *edcT* encoding a TonB dependent receptor, and other genes encoding hydratase, a lipid-transfer protein, enoyl-CoA hydratase/isomerase, 2-ketocyclohexanecarboxyl-CoA hydrolase, acetyl-CoA acyltransferase, 3-hydroxy-3-metylglutaryl-CoA synthase, 3-hydroxyacyl-CoA dehydrogenase acyl-CoA dehydrogenase, and a vicinal oxygen chelate domain responsible for multiple enzyme functions (Ibero et al., 2020). The gene *edcB* of *N. tardaugens* shares 52% and identity to gene *OecC* and *EGO55_13440* is homologous to gene *OecB*, additionally outside of the cluster genes encoding 17β-hydroxysteroid dehydrogenase with similarity to gene *OecA* in *Sphingomonas* sp. strain KC8 were identified (Chen et al., 2017; Ibero et al., 2020). Gene knock-out confirmed that genes *ecdA* coding for cytochrome P450 hydroxylase, *ecdB* encoding 4-hydroxyestrone-4,5-dioxygenase, and *ecdT* encoding a TonB receptor, are responsible for 4-hydroxylation of E1, A-ring cleavage, and oestrogen uptake, respectively (Ibero et al., 2020). More recently, gene expression experiments combined with identification of the enzymatic products of degradation revealed two monooxygenase systems EstP and EstO within the genome of *Novosphingobium* sp. ES2-1 (Li et al., 2021). EstP is a three-component cytochrome P450 monooxygenase that catalyses the oxidation of E1, whilst EstO is a two-component monooxygenase system that catalyses the oxidation of 4-hydroxyestrone (Li et al., 2021). Further, degradation of different steroids may be interlinked as gene *EstO1* which encodes EstO monooxygenase may be involved in E2, testosterone, androstenedione, progesterone, and pregnenolone degradation, however, gene *EstP1* encoding EstP monooxygenase is only involved in E2 degradation (Li et al., 2021). In actinobacteria, *Rhodococcus* sp. strain B50 the *aed* gene cluster contains genes *AedA* and *AedB* which encode cytochrome P450 monooxygenase and 4-hydroxyestrone-4,5-dioxygenase, respectively (Hsiao et al., 2021).

Genomic and transcriptomic studies have revealed numerous other genes putatively involved in oestrogen and steroid metabolism. The genome of *Stenotrophomonas maltophilia* SJTL3 contains 12 short-chain dehydrogenase/reductase (SDR) encoding genes, the superfamily of genes to which 17β-hydroxysteroid dehydrogenase belongs (Xiong et al., 2020). It also contains two 3β-hydroxysteroid dehydrogenases, five dioxygenase-encoding genes, two FAD-dependent monooxygenase-encoding genes, and 17 other dehydrogenases including acyl-CoA dehydrogenase and alcohol dehydrogenase putatively involved in the downstream degradation of oestrogens (Xiong et al., 2020). Four of the genes encoding acyl-CoA dehydrogenase within the genome of *S. maltophilia* SJTL3 showed high similarity, containing the same Y-X-X-D/E-R motif, to a 17β-hydroxysteroid dehydrogenase identified within the genome of *Sphingomonas* strain KC8 (Chen et al., 2017; Xiong et al., 2020). The genes coding for the putative enzymes catechol 1,2 dioxygenase, dioxygenase, and 7α-hydroxysteroid dehydrogenase were expressed in *Acinetobacter* sp. DSSKY-A-001 in the presence of E2 compared to a control, suggesting their involvement in the degradation of E2 (Qiu et al., 2019). Furthermore, several other genes encoding the enzymes potentially involved in oestrogen degradation were identified in the genome of the same strain (e.g., phenol hydroxylase, alkane oxygenase, short-chain dehydrogenase, catechol 2,3-dioxygenase, acetyl-CoA-acetyltransferase, and enoyl-CoA hydratase) (Qiu et al., 2019). Wang Y. et al. (2020) predicted 159 dehydrogenases, 9 monooxygenase, 6 dioxygenase, 16 hydratase, and 27 hydrolase encoding genes within the genome of the E2-degrader, *Lysinibacillus sphaericus* DH-B01. Genes encoding cholesterol oxidase, steroid Δ-isomerase, cytochrome P450 monooxygenase, 3δ-steroid-1-dehydrogenase, 3-steroid-9α-hydroxylase (KSH), and 3α,20β-hydroxysteroid steroid dehydrogenase were identified within the genome of the E1-degrading *Rhodococcus* sp. strain BH2-1 (Pratush et al., 2020). Gene expression studies confirmed up-regulation of gene *kshA* encoding a 3-indolone-9α-hydroxylase oxidase subunit, gene *kshB* encoding a 3-indolone-9α-hydroxylase reductase subunit, gene *tesI* encoding a 3-oxo-5α-steroid 4-dehydrogenase, *ksdI* encoding isomerase, *fadD3* encoding a CoA ligase, *hsaC* encoding a dioxygenase, *hsaD* encoding a hydrolase, *hsaA* encoding monooxygenase, and genes encoding 17β-hydroxysteroid dehydrogenase, 3-keto-steroid-9α hydroxylase, and aldehyde dehydrogenase were induced by oestrogen in the genome of *R. equi* DSSKP-R-001 (Zhao et al., 2018; Tian et al., 2020). Transcriptomic analysis of *R. equi* DSSKP-R-001 revealed 270 differentially expressed genes in the presence of E1, E2, and EE2 together, whilst 720, 983, and 845 genes were up-regulated in the presence of E1, E2, and EE2 each, respectively (Tian et al., 2020). The genome of *Denitratisoma* sp. strain DHT3 contains the gene cluster, *emtABCD*, which encodes a vitamin B12 dependent methyltransferase system, which was expressed in the presence of oestrogen relative to a control, and required for anaerobic catabolism of oestrogens (Wang P.H. et al., 2020).

Although the oestrogen degradation pathway(s) for bacteria remains unclear, there is some consensus that oestrogens can be degraded anaerobically *via* the 2, 3-*seco* pathway and aerobically *via* the 4, 5-*seco* pathway (Chiang et al., 2019; Wu et al., 2019). For the bacteria, *Sphingomonas* strain KC8, *P. putida* SJTE-1*/P. citronellolis* SJTE-3, *A. estronivorus* sp. nov., *S. maltophilia* SJTL3, *Rhodococcus* sp. P14, *D. actinosclerus* SJTR1, *Acinetobacter* sp. DSSKY-A-001, *Novosphingobium* sp. ES2-1, and *L. sphaericus* DH-B01, the first step in the degradation of E2 appears to be the oxidation of the C-17 hydroxyl position by an SDR such as 17β-hydroxysteroid dehydrogenase to produce E1 (Hu et al., 2011; Qin et al., 2016; Zheng et al., 2016; Chen et al., 2017; Ye et al., 2017; Xiong et al., 2018, 2020; Qiu et al., 2019; Wang et al., 2019; Wu et al., 2019; Wang Y. et al., 2020; Li et al., 2021). However, there are examples of different E2 degradation pathways reported in bacteria, such as hydroxylation at different positions within different saturated rings (B, C, or D) to produce 4-hydroxyestradiol (4-OH-E2), keto-E1, keto-E2, or E3 (Xuan et al., 2008; Kurisu et al., 2010; Yu et al., 2013). For *Sphingomonas* strain KC8 and *L. sphaericus* DH-B01, E1 is suggested to be further degraded by A ring hydroxylation by monooxygenase to produce 4-hydroxy oestrone (4-OH-E1), followed by A ring cleavage by dioxygenase (Chen et al., 2017; Wang Y. et al., 2020). Although in E1 degradation by *Acinetobacter* sp. DSSKY-A-001 the product is not 4-OH-E1, but another metabolite catabolised by oxygenase, followed by A ring cleavage (Qiu et al., 2019). Different pathways for E1 degradation are also proposed (Yu et al., 2013), for example; (i) E1 conversion by an unknown enzyme into androsta-1,4,6-triene-3,17-dione (ATD) and by isomerase into 3-hydroxyandrosta-5,7,9(11)-trien-17-one by *Rhodococcus* sp. strain BH2-1 (Pratush et al., 2020), (ii) B ring cleavage (Haiyan et al., 2007), (iii) oxidation of the saturated D ring to produce a lactone (Lee and Liu, 2002), and (iv) hydration of the saturated D ring at positions C-16 and C-17 to produce E3 (Xuan et al., 2008). However, for many of the bacteria carrying out these transformations, a genome sequence is unavailable and the pathways were suggested due to the presence of metabolites.

In this study, we wanted to examine the oestrogen degrading capacity through experimentation and genome analysis of an oestrogen-degrading isolate whose genome was previously unsequenced. We selected *R. equi* ATCC^®^ 13557^TM^ as a model organism from the limited number of oestrogen degrading bacteria (10) available at the time in culture collections (see Supplementary Tables 1, 2). This organism was identified as an EE2 degrading bacterium (Larcher and Yargeau, 2013; O’Grady et al., 2009), which had the highest removal of EE2 of those in the culture collections (see Supplementary Table 2).

Degradation experiments were carried out to confirm oestrogen metabolism by *R. equi* ATCC13557 under the experimental conditions. The genome of *R. equi* ATCC13557 was assembled and analysed for the genes identified as coding for enzymes potentially involved in oestrogen degradation from the literature, including genes identified within the genomes of *Sphingomonas* sp. strain KC8 (Hu et al., 2011) and *P. putida* strain SJTE-1 (Liang et al., 2012).

## Materials and Methods

### Chemicals

Oestrone (E1), 17β-oestradiol (E2), 17α-ethinylestradiol (EE2), M9 mineral salts, methanol, formalin, nutrient agar, yeast extract, and ringers solution, were purchased from Merck KGaA Sigma (Darmstadt, Germany). Acetonitrile and formic acid were purchased from Thermo Fisher Scientific, Fisher Scientific (Waltham, MA, United States).

### Biodegradation Assays

Two series of batch bioreactors were used to confirm the involvement of *R. equi* ATCC13557 in the metabolism of EE2 and test its ability to degrade E1 and E2.

*Rhodococcus equi* ATCC13557 (American Type Culture Collection) was acclimated to a series of oestrogens for 24 h (liquid culture at 25°C, 155 rpm) before being used as inoculum in the batch reactors. Culture media consisted of M9, Minimal Salts, 5 X MSM (Sigma-Aldrich) (33.9 g/L Na_2_HPO_4_, 15 g/L KH_2_PO_4_, 5 g/L NH_4_Cl, and 2.5 g/L NaCl), supplemented with 0.6 g/L of yeast extract. Stock solutions of EE2, E2, and mixed E1-E2-EE2 (1 g/L) were prepared in 50:50 of acetonitrile:methanol (AcN:MeOH), and added to the culture media to obtain a concentration in the media of 5 mg/L. Before their use, oestrogens stock solutions were concentrated under a stream of nitrogen, then mixed to the minimal salt (MSM) supplemented with 0.6 g/L of yeast extract, and heated on a hot plate to remove remaining traces of acetonitrile and methanol.

Sterile (autoclaved at 120°C) 0.5 L bottles constituted four series of batch bioreactors (in triplicate) inoculated with the acclimated *R. equi* ATCC13557 (liquid culture at 25°C, 155 rpm), with firstly (i) mixed E2-EE2, (ii) E2, (iii) without oestrogen, and (iv) 0.1% formalin and mixed E2-EE2. Secondly, (i) mixed E1-E2-EE2, (ii) E2, (iii) without oestrogen, and (iv) 0.1% formalin and mixed E1-E2-EE2. Batch bioreactor conditions with formalin (iv) served as a control to confirm that adsorption was not taking place and that the changes in oestrogen concentration were due to biotic factors.

Triplicate samples were regularly collected firstly for between 0 and 18 h with a final sample taken at 311 h, and then secondly between 16 and 24.5 h, with an initial sample at 0 h and a final sample at 45.5 h, and used to (i) measure bacterial growth (1 mL) using a Spectrophotometer (ATI Unicam 8625 UV/Vis), (ii) determine oestrogen concentration by uHPLC analysis (2 mL), and (iii) for colony counts (100 μL) through plating (triplicate) on nutrient agar (incubation at 25°C for 24 h).

### uHPLC Quantification

The collected samples (2 mL) were immediately centrifuged at 13,000 × *g* for 3 min, and 1 mL of supernatant filtered (0.2 μm pore size, 13 mm EMD Millipore Durapore^TM^ PVDF membrane syringe filters) into a 2 mL amber glass vial with PTFE septum (Chromacol, Supelco). A series of standard solutions for E1, E2, EE2 (1, 2.5, 5, 7.5, and 10 mg/L) were also prepared in MSM, were processed in the same way as the samples, and used to quantify the oestrogen in the bioreactor samples. Individual standard curves are reported in Supplementary Figures 1–3.

Oestrogen quantification was carried out on a Dionex uHPLC system (Thermo Scientific), using a Thermo Scientific Accucore C18 100 × 2.1 mm, 2.6 μm particle size column with the settings of; flow rate = 0.4 mL/min, temperature = 40°C, injection volume = 50–90 μL, electrochemical potential = 1800 mV. The run was isocratic using 62% of mobile phase A and 38% of mobile phase B for 4–6.5 min. Mobile phase A contained 95% water, 5% acetonitrile, and 0.1% formic acid. Mobile phase B contained 95% acetonitrile and 5% water with 0.1% formic acid. The detection limit of oestrogen was 100 ng/L.

### Statistical Analysis

All graphs were produced using SigmaPlot version 12.5, and statistical analysis was carried out using Minitab 17 (Minitab 17 Statistical Software, 2010).

The biodegradation data was determined to be non-normally distributed using the Anderson Darling normality test. A Kruskal–Wallis test was carried out to determine if the difference in optical density and CFU/mL were significantly different when comparing the treatment conditions to each other. A Pearson correlation was carried out to determine if there was a significant correlation between time and the concentration of oestrogen.

### Genome Sequencing

*Rhodococcus equi* ATCC13557 was cultured in 5 mL of nutrient broth (ATCC media 3) at 30°C for 24 h. Genomic DNA of *R. equi* ATCC13557 was extracted using a Qiagen DNeasy extraction kit (Qiagen, United States). The DNA quality was quantified using a NanoDrop spectrophotometer (Thermo Scientific, Waltham, MA, United States) and DNA quantified using a Qubit V2.0 fluorometer (Life Technologies, Carlsbad, CA, United States). Illumina sequencing library of genomic DNA was prepared using Nextera^TM^ XT DNA Library Prep Kit (Illumina, San Diego, CA, United States) using amplicons >300 bp. Library quality was validated by a Bioanalyzer high-sensitivity DNA kit (Agilent Technologies, Palo Alto, CA, United States) before sequencing. The genome of *R. equi* ATCC13557 was sequenced on Illumina MiSeq 300 bp paired-end read run on a V3 cartridge, on the “FASTQ only” setting to remove the adapter sequences (Illumina Inc., San Diego, CA, United States).

### Genome Assembly, Annotation, and Mapping

The sequences were trimmed using Trimmomatic, with an initial ILLUMINACLIP step to remove Nextera paired-end adaptor sequences (Bolger et al., 2014) and assembled using SPAdes both using usegalaxy.org (Bankevich et al., 2012; Blankenberg et al., 2014; Afgan et al., 2016). QUAST (Gurevich et al., 2013) was used to evaluate the SPAdes genome assembly. Mauve (Darling, 2004; Darling et al., 2004) was used to align the original reads back to the assembled genome. Bowtie 2 on the usegalaxy.org (Langmead and Salzberg, 2012; Blankenberg et al., 2014; Afgan et al., 2016) was used to reorder the contigs. Artemis was used to browse the genome and look for SNPs and insertions or deletions (Rutherford et al., 2000; Carver et al., 2012).

The genome assembly was submitted to RAST for annotation (Aziz et al., 2008). The CGView Comparison Tool (CCT) (Grant et al., 2012) was used to create a graphical representation of the genome, *R. equi* strain 103S was used as a reference as it was identified, in a Basic Local Alignment Search Tool (BLAST) search of the largest contig within the genome of *R. equi* ATCC13557, as the most similar complete genome.

### Determining the Most Closely Related Genomes

A Tetra Correlation Search based on tetra nucleotide frequencies and correlation coefficients was carried out to compare the genome of *R. equi* ATCC13557 against a continuously updated reference database of JSpeciesWS called GenomesDB, from which 14 genomes with the closest genomic identities were selected (Richter et al., 2016). Average nucleotide identity (ANI) analyses were performed using JSpeciesWS and BLAST (Goris et al., 2007; Camacho et al., 2009; Richter et al., 2016). The distance matrix was generated based on distance values, which were calculated by 1 – ANI value. The distance matrix was provided in Phylip format to produce the phylogenetic tree using T-REX, which was calculated using the neighbour-joining method (Saitou and Nei, 1987; Boc et al., 2012). The phylogenetic tree was created from the resulting Newick format using iTOL version 6.1.1 and was rooted at the midpoint (Farris, 1972; Letunic and Bork, 2019).

### Genomic Evaluation for Genes Coding for Enzymes Potentially Involved in Steroid Hormone Biodegradation

The RAST SEED viewer was used to search for the genes identified in the literature that may encode enzymes involved in oestrogen degradation (Table 1; Aziz et al., 2008; Overbeek et al., 2014; Brettin et al., 2015). Cluster Locator was used to determine the statistical significance of an identified gene cluster, using a maximum gap of 5 (Obregón et al., 2018).

**TABLE 1:** The major enzymes potentially involved in oestrogen degradation.

Metabolic step	Suggested enzyme	EC number	References
EE2 to 2-OH-EE2	Dioxygenase	1.13.11.-	Yi and Harper, 2007
EE2 to E1	*Oxygenase	1.13.- or 1.14.-	Haiyan et al., 2007
E1 to E3	Hydratase	4.2.1.-	Xuan et al., 2008; Adeel et al., 2017
E2 to E3	Hydroxylase	1.14.16.-	Xuan et al., 2008; Adeel et al., 2017
E2 to keto E2	Hydroxylase	1.14.16.-	Kurisu et al., 2010
E2 to 4-OH-E2	Dioxygenase	1.13.11.-	Kurisu et al., 2010
E2 to E1	17β-hydroxysteroid dehydrogenase	1.1.1.51	Lee and Liu, 2002; Ke et al., 2007; Yu et al., 2007; Xiong et al., 2018; Qiu et al., 2019
E1 to 4-hydroxy-E1	Dioxygenase/P450 monooxygenase	1.13.11.-	Coombe et al., 1966; Yu et al., 2016; Chen et al., 2017; Hsiao et al., 2020; Ibero et al., 2020; Li et al., 2021
E1 to 4-[3a-methyl-3,7-dodecane-6H-cyclopentadiene(a)naphthalene-6-Subunit]-2-methoxy-3-butenoic acid	Oxygenase	1.13.- or 1.14.-	Qiu et al., 2019
4-[3a-methyl-3,7-dodecane-6H-cyclopentadiene(a)naphthalene-6-Subunit]-2-methoxy-3-butenoic acid to (Z)-8-(7a-methyl-1n 5-dioxo-octahydro-1H-inden-4-yl)-2o-6-dioxy-4-butenoic acid	Oxygenase	1.13.- or 1.14.-	Qiu et al., 2019
E1 to 3-HAS	Hydroxylase	1.14.16.-	Haiyan et al., 2007
3-HSA to 3,4-DHSA	Hydroxylase	1.14.16.-	Horinouchi et al., 2004
3,4-DHSA to 4,9-DHSA	Dioxygenase	1.13.11.-	Horinouchi et al., 2001
4,9-DHSA to 2-hydroxyhexa-2,4-dienoic acid and 3-[(3aS, 7aS)-7a-methyl-1,5-dioxooctahydro-1H-inden-4-yl] propanoate	Hydrolase	3.7.1.-	Horinouchi et al., 2003a,b
2-hydroxyhexa-2,4-dienoic acid to 4-hydroxy-2-oxohexanoate	Hydratase	4.2.1.-	Horinouchi et al., 2005
4-hydroxy-2-oxohexanoate to the p-xylene pathway	BphI and BphJ (aldolase and dehydrogenase complex)	4.1.3.43	Horinouchi et al., 2005; Carere et al., 2011

*Suggested oxygenation of the ethinyl group, however, a specific enzyme is unknown.

A database of genes containing enzymes potentially involved in oestrogen degradation was compiled using the current literature about oestrogen degradation (Table 1). In addition to these, 3-ketosteroid Δ1-dehydrogenase, also named 3-oxosteroid 1-dehydrogenase, responsible for the conversion of 4-AD to androsta-1,4-diene-3,17-dione (ADD) within the testosterone degradation pathway, was included in a database of potential oestrogen degradation genes due to up-regulation when exposed to E2 (Sang et al., 2011). Gene sequences identified in the genome of *C. testosteroni* (Horinouchi et al., 2003a,b, 2004, 2005, 2010) and the two genome sequences of the oestrogen degrading bacteria *P. putida* SJTE-1 (Liang et al., 2012) and *Sphingomonas* strain KC8 (Hu et al., 2011), along with the PAH dioxygenase encoding genes (Meynet et al., 2015) and dehydrogenase encoding genes (Kisiela et al., 2012) were stored in FASTA format. The compiled list was then used to search for other genes with at least 85% sequence similarity and any BLAST results were added to the list, the list of sequences can be found in Supplementary Tables 4, 5, these included monooxygenase and isomerase due to their similarities to dioxygenase and dehydrogenase, respectively. A RefSeq ID list is provided for some of the genes encoding for dehydrogenase in Supplementary Table 6 (Kisiela et al., 2012).

Analysis of the *R. equi* ATCC13557 genome was carried out using BLAST in BioLinux to search for the presence of the genes encoding the enzymes within the compiled lists, the sequence lists can be found in Supplementary Tables 4–6, which are potentially involved oestrogen degradation. Additionally, BLASTP in RAST (Altschul et al., 1997) was used to search the genome specifically for the presence of the genes *oecA*, *oecB*, and *oecC* involved in oestrogen (E2 and E1) degradation, which were identified within the genome of *Sphingomonas* strain KC8 (Chen et al., 2017). The amino acid sequences for 3β, 17β-hydroxysteroid dehydrogenase gene (*oecA*; KC8_09390), flavin-dependent monooxygenase (oestrone 4-hydroxylase) (*oecB;* KC8_16650), and extradiol dioxygenase (4-hydroxyestrone-4,5-dioxygenase) (*oecC;* (KC8_05325), were obtained from NCBI accession number CP016306 (Chen et al., 2017). The nucleic acid sequences, from the genomes of bacteria known to degrade oestrogen and *R. equi* ATCC13557, of genes encoding the enzymes 3β, 17β-hydroxysteroid dehydrogenase, cytochrome P450 monooxygenase (oestrone 4-hydroxylase), and extradiol dioxygenase (4-hydroxyestrone-4,5-dioxygenase) were compiled into a fasta file. The nucleic acid sequences from non-oestrogen degrading bacterial genomes of genes encoding (i) P450 monooxygenase (*BisdB*), (ii) 3-ketosteroid-delta-1-dehydrogenase (*kstD* and *TesH*), (iii) 3-ketosteroid-Δ^4^(5α)-dehydrogenase (TesI), and (iv) dioxygenase (*HsaC*), with the functions of bisphenol A, cholate, testosterone, and cholesterol degradation (Horinouchi et al., 2003b; Van der Geize et al., 2007; Sasaki et al., 2008; Mohn et al., 2012), respectively, were added to this file for comparison. These sequences were used to construct phylogenetic trees using Phylogeny.fr, which included the steps MUSCLE for multiple alignments, Gblocks to remove poorly aligned and divergent regions, PhyML for phylogenetic reconstruction, and finally, the production of a phylogenetic tree using TreeDyn (Dereeper et al., 2008). The amino acid sequences of genes, *cyp125*, *ipdA, ipdB, ipdC, hsaA, hsaB, hsaC, hsaD, fadD3, echA20, kstR, kstR2*, and *mce4A-E*, involved in cholesterol degradation by *R*hodococcus *jostii* RHA1 (Van der Geize et al., 2007; Rosłoniec et al., 2009), were downloaded from UniProt into a fasta file (The UniProt Consortium, 2021). These sequences were then used to search the genome of *R. equi* ATCC13557 using BLASTP in RAST (Altschul et al., 1997).

M1CR0B1AL1Z3R (Avram et al., 2019) was used to compare, mine, and analyse *R. equi* ATCC13557 and the genomes of other oestrogen degrading bacteria whose sequences were retrieved from GenBank; *A. estronivorus* strain MH-B5, *Sphingobium estronivorans* sp. strain AXB, *N. tardaugens* NBRC 16725, *R. equi* DSSKP-R-001, *Rhodococcus* sp. strain B50 and *Sphingomonas* strain KC8 (Hu et al., 2011; Chen et al., 2017; Zhao et al., 2018; Ibero et al., 2020; Qin et al., 2020; Tian et al., 2020). Also to compare, mine, and analyse *R. equi* ATCC13557 and the genomes of bacteria previously found to possess cholesterol biotransformation potential; *R. jostii* RHA1, *R. equi* 103S, *Mycobacterium tuberculosis* H37Rv, *Rhodococcus* sp. strain B50, and *R. equi* DSSKP-R-001 (Van der Geize et al., 2007, 2011; Rosłoniec et al., 2009; Hsiao et al., 2021). ORFs were extracted from the M1CR0B1AL1Z3R output (Avram et al., 2019) and their distributions were analysed for common ORFs that may be involved in oestrogen degradation. Also, ANI analyses were carried out, using the Kostas Lab ANI calculator tool, to compare the genome of *R. equi* ATCC13557 with these six genomes of oestrogen degrading bacteria (Goris et al., 2007; Rodriguez-R and Konstantinidis, 2014; Rodriguez-R and Konstantinidis, 2016).

## Results

### Biodegradation of Oestrogen by *R. equi* ATCC13557

**FIGURE 1:**
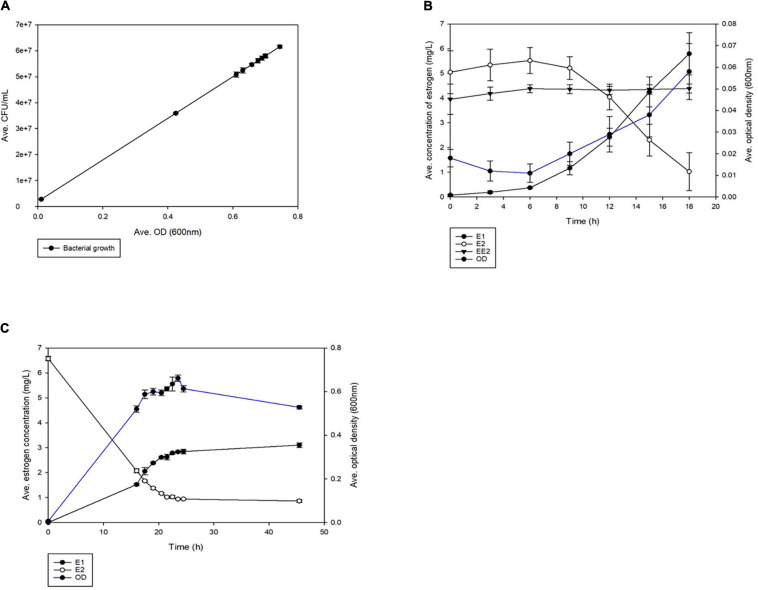
The average growth of *R. equi* ATCC13557 **(A)** whilst being grown in the different conditions, exposed to mixed oestrogens E1, E2, and EE2 and E2 only. Comparison of the average growth of *R. equi* ATCC13557 without oestrogen (control), exposed to mixed oestrogens E2 and EE2, E2-only, and abiotic. The average concentrations of oestrogens were measured over time, in mixed conditions E2 and EE2 **(B)**, and in E2-only condition **(C)**. The error bars represent standard deviation.

*Rhodococcus equi* ATCC13557 was able to grow (Figure 1) in the presence of a mixture of oestrogens (E2 and EE2) or E2 alone. There was no bacterial growth in the abiotic controls (Supplementary Figure 4). Growth occurred between 16 and 23.5 h and the yield (measured by OD) was statistically indistinguishable (*P* = 0.0639) (Figure 1A), with a maximum average yield of 5.9 × 10^7^ CFU/mL. It should be noted that while this biotransformation was associated with cell growth there were other carbon sources available in the medium from the yeast extract, which may suggest that the biotransformation was co-metabolic. However, the growth of *R. equi* ATCC13557 in the E2-only condition, as measured by OD, was significantly greater than the control without oestrogen between 12 and 15 h and at 311 h (*P* = 0.000) (Supplementary Figure 5). *R. equi* ATCC13557 was unable to degrade EE2 when grown in a mixture of E2 and EE2 (Figure 1), despite previously showing the ability to degrade EE2 (O’Grady et al., 2009; Larcher and Yargeau, 2013). However, the growth of the strain was associated with a significant decrease in the concentration of E2 in mixed oestrogen, E2-EE2 condition (*P* = 0.025, Figure 1B), and the E2-only condition (*P* = 0.000, Figure 1C). Again, the growth of the strain was also associated with a significant decrease in the concentration of E2 in the mixed oestrogen (E1-E2-EE2) condition (*P* = 0.001, Supplementary Figure 6) between 16 and 24.5 h. There was no change in the average concentration of EE2 in any condition (Figure 1B and Supplementary Figure 6). The average decrease in E2 correlated significantly with the increase in E1, occurring between 0 and 24.5 h (*P* = 0.0001, Figures 1B,C and Supplementary Figure 6), demonstrating that E2 was converted to E1. However, not all of the E2 was converted into E1 in both mixed (11.2%) and single substrate conditions (50.6%). Additionally, an unknown metabolite peak was detected, at a retention time of 1.425 min, between 16 and 45.5 h (peak area at 16 h: 50.196 nA × min, and peak area at 45.5 h: 43.4908 nA × min, see Supplementary Figures 7, 8).

### Genome Characteristics

**FIGURE 2:**
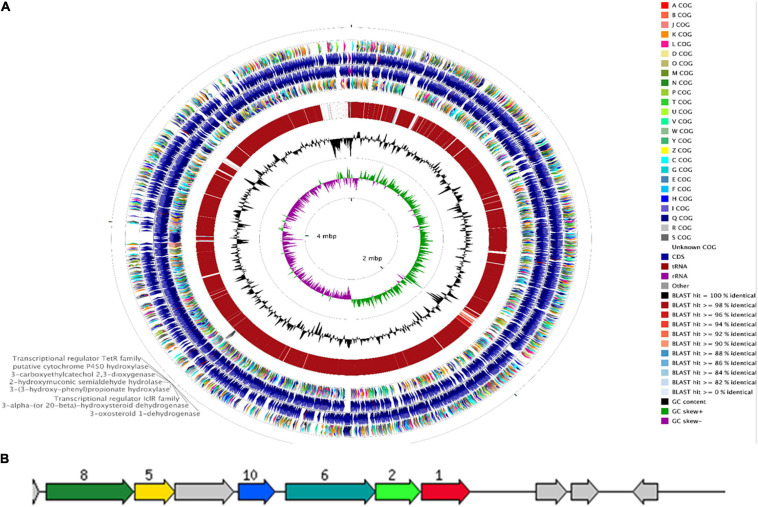
The CCT (Grant et al., 2012) genome map comparing *R. equi* ATCC 13557 to the *R. equi* 103S reference genome **(A)**. Starting from the outermost ring the feature rings depict; 1. COG features of the forward strand sequence; 2. forward strand sequence features of *R. equi* ATTC 13557; 3. reverse strand sequence features of *R. equi* ATCC 13557; 4. the COG features of the reverse strand sequence; 5. the sequence similarity was detected by BLAST comparisons conducted between nucleotide sequences from *R. equi* ATCC 13557 and *R. equi* 103S; and the final rings display the GC content and the GC skew. A gene cluster encoding enzymes potentially involved in oestrogen degradation is labelled on the outermost ring. A RAST diagram of a chromosomal region around the focus gene coding for 3-carboxyethylcatechol 2,3-dioxygenase “JO861_14995” (red, 1) **(B)**. The other genes present code for 2-hydroxymuconic semialdehyde hydrolase “JO861_15000” (light green, 2), 3-alpha-(or 20-beta)-hydroxysteroid dehydrogenase “JO861_15020” (yellow, 5), 3-(3-hydroxy-phenyl) propionate hydroxylase “JO861_15005” (turquoise, 6), 3-oxosteroid 1-dehydrogenase “JO861_15025” (dark green, 8), and transcriptional regulator IcIR family (blue, 10). The grey arrows are genes with the relative position conserved found in at least four other species.

**FIGURE 3:**
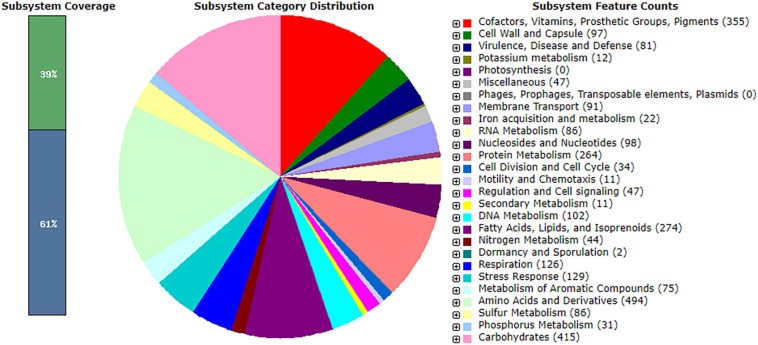
The subsystem distribution, coverage, and counts within the SPAdes assembly of *R. equi* ATCC13557 as annotated by Rapid Annotation System Technology (RAST) server (Aziz et al., 2008; Overbeek et al., 2014; Brettin et al., 2015).

The genome of *R. equi* ATCC13557 consists of 5.29 Mb, with an estimated genome coverage of 105×, an N50 contig length of 195 Kb, and a total of 173 contigs (Figure 2 and Table 2). The longest contig is 468.2 Kb, and 45 contigs are over 1000 bp in length (Table 2). There are 4927 genes identified as predicted coding sequences (CDSs) and 413 subsystems (Supplementary Data Sheet 1). There are 75 pathways for the metabolism of aromatic compounds, of which 14 are peripheral pathways and 59 are involved in the metabolism of central aromatic intermediates, which could assist in the degradation of steroids (Figure 3). This Whole Genome Shotgun project has been deposited at DDBJ/ENA/GenBank under the accession JAFFSZ000000000. The version described in this article is version JAFFSZ010000000.

**TABLE 2:** Assembly statistics for the SPAdes assembled *R. equi* ATCC13557 genome.

Statistics	Contigs
# contigs	173
# contigs (≥0 bp)	173
# contigs (≥1000 bp)	45
Largest contig	468,179
Total length	5,285,963
Total length (≥0 bp)	5,285,963
Total length (≥1000 bp)	5,231,681
N50	194,986
N75	117,183
L50	9
L75	18
GC (%)	68.51
Mismatches	
# N’s	0
# N’s per 100 kbp	0

**FIGURE 4:**
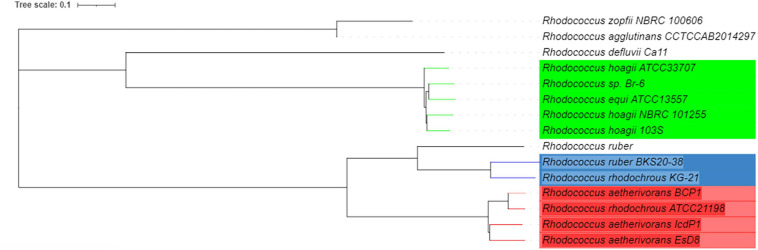
A phylogenetic tree of *R. equi* ATCC13557 and genomes with the closest genomic identities which were generated by T-REX and visualised in iTOL version 6.1.1, based on ANI analyses (Boc et al., 2012; Richter et al., 2016; Letunic and Bork, 2019).

According to ANI analyses based on BLAST (Richter et al., 2016) *Rhodococcus* sp. Br-6, *Rhodococcus hoagii* ATCC 33707, *R. hoagii* 103S, and *R. hoagii* NBRC 101255 were the genomes most closely related to *R. equi* ATCC13557, these have been highlighted green (Figure 4). There were no other genomes with significant similarity (>95%) to *R. equi* ATCC13557, however, there were two other groups that were significantly related to each other, highlighted red and blue (Figure 4).

### Genome Evaluation for Genes Coding for Enzymes Potentially Involved in Oestrogen Degradation

#### The Presence of a Cluster Containing Potential Oestrogen Degradation Genes in the Genome of *R. equi* ATCC13557

RAST annotation suggested the presence of a gene cluster, containing some genes coding for enzymes potentially involved in oestrogen degradation (Table 1 and Figure 2). The gene cluster was determined as statistically significant (*P* = 4.5944e-03) (Obregón et al., 2018). Further increasing the number of genes surrounding the cluster, to a total of 16 genes, revealed that the cluster may begin at a gene (JO861_14960) encoding acyl-CoA thioesterase and ending at a gene (JO861_15070) encoding ribose-5-phosphate isomerase (*P* = 1.1196e-02) (Obregón et al., 2018). The presence of the genes as a cluster suggests that they are evolutionarily conserved, like the cholate and cholesterol degradation pathways which are located on a single gene cluster, within the genome of *R. equi* (Bergstrand et al., 2016). The presence of genes, encoding oestrogen degradation enzymes, within gene clusters were identified in the genomes of *Sphingomonas* strain KC8, *N. tardaugens*, and *A. estronivorus* (Chen et al., 2017). The gene cluster within *R. equi* ATCC13557 contains genes coding for the enzymes potentially involved in oestrogen degradation (Table 1); 3-carboxyethylcatechol 2,3-dioxygenase (EC 1.13.11.16) which catalyses an oxidation reaction by the addition of two oxygen molecules; cytochrome P450 monooxygenase (EC 1.14.14.1) which catalyses an oxidation reaction; 2-hydroxymuconic semialdehyde hydrolase (EC 3.7.1.9) catalyses the hydrolysis of carbon-carbon bonds in ketonic substances; 3-alpha-(or 20-beta)-hydroxysteroid dehydrogenase is involved in steroid degradation by acting on the CH-OH groups with NAD+ or NADP+ as an acceptor; 3-(3-hydroxy-phenyl)propionate hydroxylase (EC 1.14.13.127) is involved in the degradation of aromatic compounds by incorporation or reduction of oxygen; 3-oxosteroid 1-dehydrogenase (EC 1.3.99.4), named 3-ketosteroid Δ1-dehydrogenase within the testosterone degradation pathway (Horinouchi et al., 2012), by acting upon CH-CH groups (Kanehisa and Goto, 2000; Kanehisa et al., 2016, 2017); and the IcIR family, transcriptional regulator, regulates the genes involved in the degradation of aromatic compounds (Molina-Henares et al., 2006). Genes encoding several enzyme classes, which contain enzymes with similar functions, potentially involved in oestrogen degradation, dioxygenase, hydroxylase, 17β-hydroxysteroid dehydrogenase, and hydrolase (Table 1) were identified within the genome of *R. equi* ATCC13557.

#### The Identification of Genes Potentially Coding for Enzymes Involved in Oestrogen Degradation in the Genomes of *R. equi* ATCC13557, *Sphingomonas* Strain KC8, and *P. putida* SJTE-1

**FIGURE 5:**
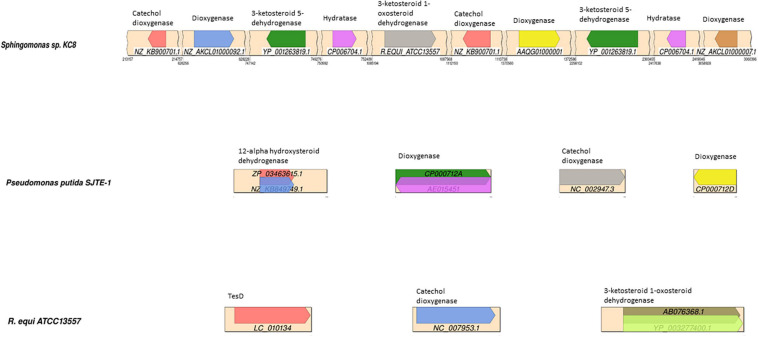
Synteny plots showing the BLAST hits of gene sequences in a database of potential oestrogen degradation genes, mapped to the contigs of; *Sphingomonas* strain KC8, *Pseudomonas putida* SJTE-1, and *R. equi* ATCC13557. The diagram of *R. equi* ATCC13557 shows the two highest-scoring BLAST sequences encoding 3-ketosteroid 1-oxosteroid dehydrogenase. The different coloured arrows represent the different sequences, with the sequence accession denoted below, encoding enzymes denoted above each arrow.

Protein BLAST (BLASTP) comparisons of the *R. equi* ATCC 13557, *Sphingomonas* strain KC8, and *P. putida* SJTE-1 genomes with a database of gene sequences coding for enzymes potentially involved in oestrogen degradation were carried out in Bio-Linux 8.0 software (Field et al., 2006). The BLASTP hits above 70% sequence similarity are summarised and the genes with significant similarity, having a low E-value and large coverage were mapped (Figure 5).

For *R. equi* there are 11 sequences with sequence similarity >70% to 3-ketosteroid 1-dehydrogenase (3-oxosteroid 1-dehydrogenase), which within the degradation pathway of testosterone catalyses the conversion of 4-AD to ADD, within other species of *Rhodococcus* and also in *Mycobacterium ulcerans* Agy99, all of which are from the phylogenetically related mycolic-acid containing sub-order *Corynebacterineae* (Figure 5). Although other genes showed similarity to genes coding for enzymes involved in steroid degradation, such as hydratase (*TesD*), which catalyses the conversion of 4,9-DHSA to 2-hydroxyhexa-2,4-dienoic acid and 3-[(3aS, 7aS)-7a-methyl-1,5-dioxooctahydro-1H-inden-4-yl] propanoate (Table 1), the sequence alignment length is very small (14 amino acids) suggesting only a small length of the gene had similarity and is likely a conserved sequence motif (Horinouchi et al., 2003a; Xiong et al., 2020). However, this may be important when considering the design of primers to target genes coding for enzymes involved in steroid degradation.

In *Sphingomonas* strain KC8, the genes with significant sequence similarity, greater than 70% and 0.0 e^–10^, to those in the database of genes coding for enzymes potentially involved in oestrogen degradation were different from those found in *R. equi*; 3-ketosteroid 5-dehydrogenase (71.1%) was identified in another *Sphingomonas* species, *Sphingomonas wittichii* RW1, and a dioxygenase (84.8%) present in *Pseudomonas* sp. SKA58 (Figure 5). All of these genes with significant sequence similarity were from *Proteobacteria*, which is a group phylogenetically distinct and unrelated to the sub-order *Corynebacterineae*, to which *R. equi* belongs. However, the gene coding for 3-ketosteroid 1-dehydrogenase of *R. equi* ATCC13557 shared 71.1% sequence similarity with *Sphingomonas* strain KC8 but had an E value of 5.9 over a small sequence length of 17 amino acids.

In *P. putida* SJTE-1, the genes with significant sequence similarity, greater than 70% and 0.0 e^–10^, to those in the database of genes encoding enzymes potentially involved in oestrogen degradation were two genes coding for dioxygenase (100 and 96.9%) and a gene coding for catechol dioxygenase (98.1%) present in other species of *Pseudomonas* (Figure 5).

When the genomes of *R. equi* ATCC13557, *P. putida* SJTE-1, and *Sphingomonas* strain KC8 were compared to each other, there was no presence of genes coding for the following enzymes putatively involved in oestrogen degradation; hydroxylase, responsible for the conversion of E2 to E3, E2 to keto-E2, E1 to 3-HSA, and 3-HSA to 3,4-DHSA (Table 1); monooxygenase, included due to similarity to dioxygenase sequences; or cytochrome P450, included as it may have a role in the conversion of E1 to 16α-hydroxyestrone (Simpson et al., 1994; Xu et al., 2005; Thomas and Potter, 2013). However, the presence of genes coding for the following enzymes suggested to be involved in oestrogen degradation was discovered: dioxygenase, catalysing the conversion of EE2 to 2-OH-EE2, E2 to 4-OH-E2, E1 to 4-OH-E1 (Table 1); dehydrogenase, catalysing the conversion of E2 to E1 (Table 1); hydratase, catalyses the conversion of E1 to E3; hydrolase, catalysing the conversion of 4,9-DHSA to 2-hydroxyhexa-2,4-dienoic acid and 3-[(3aS, 7aS)-7a-methyl-1,5-dioxooctahydro-1H-inden-4-yl] propanoate (Table 1); and isomerase, included due to similarity to dehydrogenase. There are five genes coding for hydratases present in all three genomes and seven genes coding for isomerase present in all three, with one gene coding for isomerase being present in *R. equi* ATCC13557 and *P. putida* SJTE-1.

#### Genomic Comparison of *R. equi* ATCC13557 and Six Other Oestrogen Degrading Bacteria

**FIGURE 6:**
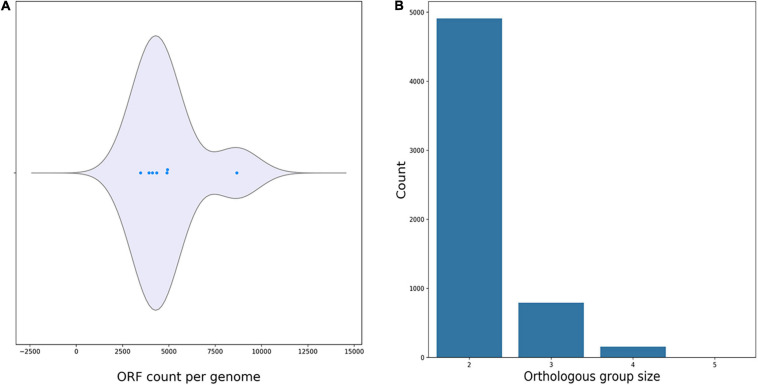
Plots produced by M1CR0B1AL1Z3R (Avram et al., 2019) showing the ORF count per genome **(A)**, and orthologous groups shared between the genomes of oestrogen degrading bacteria **(B)**.

Average nucleotide identity analyses comparing *R. equi* ATCC13557 with the genomes of the oestrogen degrading bacteria *A. estronivorus* strain MH-B5, *S. estronivorans* sp. strain AXB, *N. tardaugens* NBRC 16725, *R. equi* DSSKP-R-001, *Rhodococcus* sp. strain B50, and *Sphingomonas* strain KC8 (Hu et al., 2011; Chen et al., 2017; Zhao et al., 2018; Ibero et al., 2020; Qin et al., 2020; Tian et al., 2020), revealed that there were significant genomic identities between *R. equi* ATCC13557 and *R. equi* DSSKP-R-001 (99.05%). However, there were not significant genomic identities (>95%) shared between *R. equi* ATCC13557 and the other genomes. The ORF count distributions were relatively similar ranging from 3475 to 4927 ORFs, however, there were many more ORFs present in the genome of *Rhodococcus* sp. strain B50 with an ORF count of 8669 (Figure 6A). There are almost 5000 ORFs shared between two of the genomes of oestrogen degrading bacteria, almost 1000 ORFs shared between three of the genomes, and around 200 ORFs shared between four of the genomes, however, only one ORF was shared between five genomes and there were none shared between all of the genomes (Figure 6B). However, the ORFs shared were usually between bacteria from the same phyla, between proteobacteria or actinobacteria, and only one ORF, which encoded the DNA repair recombinase RecA, common between proteobacteria which was also present in the genome of *Rhodococcus* sp. strain B50. ORF comparisons revealed that the gene cluster present within the genomes of both *Rhodococcus* sp. strain B50 and *R. equi* DSSKP-R-001 was not present in the genome of *R. equi* ATCC13557 (Zhao et al., 2018; Hsiao et al., 2020, 2021).

#### Genomic Evaluation for Genes With Homology to *oecA*, *oecB*, and *oecC*

**FIGURE 7:**
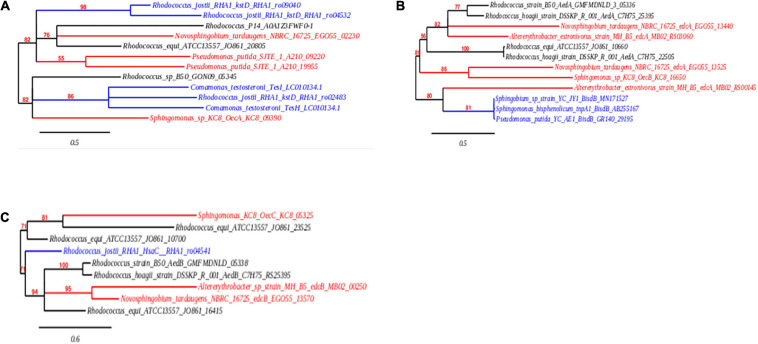
Phylogenetic trees showing the evolutionary relationship, in the orthologous genes encoding 17β-hydroxysteroid dehydrogenase **(A)**, cytochrome P450 monooxygenase **(B)**, and extradiol dioxygenase **(C)**. The nucleic acid sequences from actinobacteria are shown in black, proteobacteria are shown in red, and non-oestrogen degrading bacterial genomes of genes encoding (i) P450 monooxygenase (*BisdB)*, (ii) 3-ketosteroid-delta-1-dehydrogenase (*kstD* and *TesH)*, (iii) 3-ketosteroid-Δ^4^(5α)-dehydrogenase (TesI), and (iv) dioxygenase (*HsaC)*, which function in bisphenol A, cholate, testosterone, and cholesterol degradation, respectively, are shown in blue.

Whilst the gene cluster present within *R. equi* DSSKP-R-001 and *Rhodococcus* sp. strain B50 was not identified in the ORF comparison of the genomes within the genome of *R. equi* ATCC13557 (Zhao et al., 2018; Hsiao et al., 2020, 2021; Tian et al., 2020), there were similarities in genes identified as coding for enzymes responsible for E2 conversion to E1 (*oecA*), and the further degradation of E1 (*oecB* and *oecC*). In this study, homologous genes to *oecA* and *oecB* were identified in the genome of *R. equi* ATCC13557, one sequence with 46% amino acid sequence similarity to gene *oecA* and a sequence with 40% amino acid sequence similarity to gene *oecB* (Table 3). There were no amino acid sequences identified with similarity >40% to gene *oecC*, however, there were several sequences (4) with low similarity 34–38% (Table 3). Phylogenetic tree construction revealed that the sequences in the genome of *R. equi* ATCC13557, with similarity to the gene encoding 17β-hydroxysteroid dehydrogenase (*oecA*), was closely related to *Rhodococcus* sp. P14 and *N. tardaugens* NBRC16725 (Figure 7A), and that with similarity to the gene encoding P450 monooxygenase (*oecB*), was closely related to *Rhodococcus* sp. DSSKP-R-001 (Figure 7B). Also, of the genes with similarity to extradiol dioxygenase, genes (JO861_23525 and JO861_10700) were more closely related to *Sphingomonas* strain KC8, whilst the other gene (JO861_16415) identified with similarity to extradiol dioxygenase (*oecC*) was distinct, and more closely related to the genes of *Rhodococcus* strain B50, *R. equi* DSSK-R-001, *Altererythrobacter* sp. strain MH-B5, and *N. tardaugens* NBRC16725 (Table 3 and Figure 7). The gene with similarity to P450 monooxygenase and one of the genes with similarity to extradiol dioxygenase (JO861_10700) are clustered together within the genome of *R. equi* ATCC13557. The presence of these oec-like genes, in the genome of *R. equi* ATCC13557, and their phylogenetic relationship with orthologous genes in other oestrogen degrading bacteria (Figure 7), suggest that oestrogen degradation is carried out *via* a similar pathway in both proteobacteria and actinobacteria.

**TABLE 3:** Genes located in the genome of *R. equi* ATCC13557 with similarity to *oecA*, *oecB*, and *oecC*.

Contig	Locus tag	Start	End	BLASTP alignment	Percentage similarity (%)	E value
NODE_23_length_81690_cov_215.308863	JO861_20805	11,811	10,648	*oecA*	46	e-117
NODE_7_length_250389_cov_226.591626	JO861_10660	197,128	195,962	*oecB*	40	e-102
NODE_33_length_40541_cov_222.929037	JO861_23525	22,067	22,948	*oecC*	38	4e-56
NODE_5_length_279867_cov_223.006136	JO861_08165	232,647	231,745	*oecC*	35	5e-54
NODE_7_length_250389_cov_226.591626	JO861_10700	204,627	203,722	*oecC*	34	1e-46
NODE_14_length_140806_cov_217.300509	JO861_16415	100,943	101,875	*oecC*	35	2e-45

#### Genomic Evaluation for Genes Involved in the Biodegradation of Other Steroid Hormones

**FIGURE 8:**
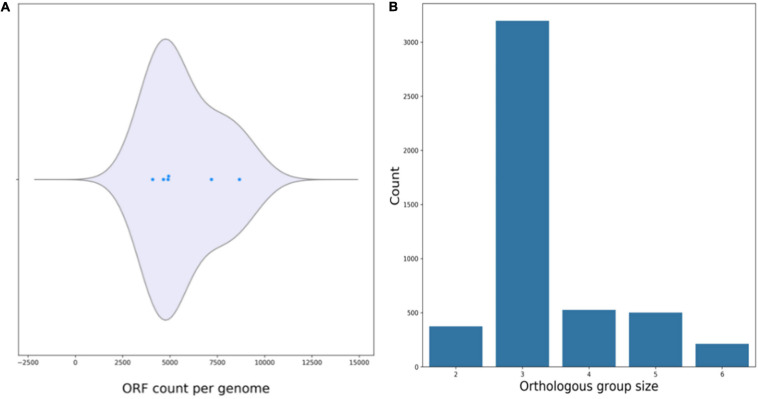
Plots produced by M1CR0B1AL1Z3R (Avram et al., 2019) showing the ORF count per genome **(A)**, and orthologous groups shared between the genomes of cholesterol degrading bacteria **(B)**.

Genomic comparisons of *R. equi* ATCC13557 with bacteria with the potential to biotransform cholesterol, *R. jostii* RHA1, *R. equi* 103S, *M. tuberculosis* H37Rv, *Rhodococcus* sp. strain B50, and *R. equi* DSSKP-R-001 (Van der Geize et al., 2007; Rosłoniec et al., 2009; Van der Geize et al., 2011; Hsiao et al., 2021), revealed that the ORF count distributions were relatively similar ranging from 4085 to 4927 ORF’s, however, there were many more ORFs present in the genomes of *Rhodococcus* sp. strain B50 and *R. jostii* RHA1 with ORF counts of 8669 and 7191, respectively (Figure 8A). There are almost 400 ORFs shared between two of the genomes of cholesterol degrading bacteria, almost 4000 ORFs shared between three of the genomes, around 500 ORFs shared between four and five of the genomes, and around 250 ORFs shared between all six genomes (Figure 8B). There was a large number, without a gap, of ORFs, shared between *R. equi* ATCC13557 and three to five of the rhodococci, starting at JO861_07330 and ending at JO861_08275. Further analysis revealed that this span of ORFs on contig 5, contained gene clusters for cholesterol degradation. The first cluster (JO861_07825 to JO861_07895) housed the *mce4* genes responsible for cholesterol import (Pandey and Sassetti, 2008). The next cluster (JO861_07930_JO861_08025) contained genes encoding for enzymes responsible for cholesterol side-chain, A/B ring, and C/D ring degradation; *cyp125* (JO861_07960), *echA20* (JO861_07990). *ipdA* (JO861_07995), *ipdB* (JO861_08000), and *ipdC* (JO861_08005) (Rosłoniec et al., 2009; Wilburn et al., 2018). The third (JO861_08050 to JO861_08090) contained the *kst*R2 and *fadD*3 genes encoding a transcription repressor and an enzyme responsible for cholesterol C/D ring degradation (Casabon et al., 2013). Further, the *hsaA, hsaB, hsaC*, and *hsaD* genes responsible for the A/B cholesterol ring cleavage were found in a cluster (JO861_08165 to JO861_08190) (Van der Geize et al., 2007). These gene clusters were determined as statistically significant (*P* = 5.5231e-04, 8.1835e-04, 4.4097e-04, and 5.0946e-04), respectively (Obregón et al., 2018).

## Discussion

In this study, conversion of E2 to E1 as the major metabolite was observed in biodegradation experiments, with the average increase in the concentration of E1 correlating significantly to the average decrease in E2. There was no observed degradation of EE2 and E1 did not appear to be further degraded. However, the degradation of E2 in this instance may be co-metabolic since other carbon sources were present in the growth medium. It has been previously reported that *R. equi* ATCC13557 partially degraded EE2 (up to 60%) in the presence of a co-substrate, within 300 h (O’Grady et al., 2009; Larcher and Yargeau, 2013). The absence of EE2 degradation in *R. equi* ATCC13557 is different than previously reported. The presence of 0.5% (v/v) ethanol in the other study may have increased oestrogen degradation of EE2 *via* co-metabolism (Larcher and Yargeau, 2013). The findings here were similar to the conversion of E2 to E1 by other rhodococci; *Rhodococcus* sp. P14 (Ye et al., 2017), *Rhodococcus* sp. strain B50 (Hsiao et al., 2020), *Rhodococcus* sp. strain DSSKP-R-001 (Zhao et al., 2018; Tian et al., 2020), the latter of which shared significant genome identity with *R. equi* ATCC13557 in this study. In one of these studies, pH, inoculum concentration, and temperature had effects upon the degradation of E2 by *Rhodococcus* sp. DS201 (Yu et al., 2016), which may have influenced the differences observed between this and previous studies on *R. equi* ATCC13557. In the biodegradation experiments, there was no change in the average concentrations of oestrogen within the abiotic control, so it can be concluded that all changes in oestrogen concentration within the experimental conditions are due to biotic factors. In the mixed oestrogen condition, the conversion from E2 to E1 was almost complete, with little, 11.2% of E2, not converted into E1. However in the E2-only condition, the amount of E1 detected was much less than the amount of E2 degraded (50.6%), suggesting that other metabolites were produced or complete mineralisation occurred. Another metabolite was detected in the E2-only condition that may account for some of the difference in E2 and E1 concentration, however, the metabolite was not identified or quantified to perform a mass balance. A similar additional peak with a retention time similar to that of E3 has been reported previously, however, this was under anaerobic conditions using lake sediment and sludge (Czajka and Londry, 2006), though the conversion of E1 and E2 to E3 has been reported in soil (Xuan et al., 2008). Furthermore, the degradation of E2 was associated with significant growth in the E2-only condition.

The pathway of oestrogen degradation for *R. equi* ATCC13557 can be postulated based on the above-observed biodegradation characteristics of *R. equi* ATCC13557 and the presence of certain genes. The main metabolite detectable was E1, suggesting that the first step in oestrogen metabolism by *R. equi* ATCC13557 involves 17β-hydroxysteroid dehydrogenase (Table 1), which has the function of converting E2 to E1 (Ye et al., 2017). BLASTP searches of the *R. equi* ATCC13557 genome revealed genes with similarity to genes encoding 17β-hydroxysteroid dehydrogenase, cytochrome P450 monooxygenase, and extradiol dioxygenase (see, Table 3), which were determined in the literature as functionally active in the metabolism of oestrogens E2 and E1 by *Sphingomonas* strain KC8 (Chen et al., 2017). The gene (JO861_10700) encoding extradiol dioxygenase is clustered with the gene encoding P450 monooxygenase, like those of *Rhodococcus* sp. DSSKP-R-001 and *Rhodococcus* sp. B50 (Zhao et al., 2018; Hsiao et al., 2021), in this study. Comparably, the gene *oecA* of *Sphingomonas* strain KC8 and the gene of *N. tardaugens* NBRC 16725 encoding 17β-hydroxysteroid dehydrogenase were both outside of the main gene cluster, like the gene in this study (Chen et al., 2017; Ibero et al., 2020). Phylogenetic trees were constructed to confirm the evolutionary relationship of these gene sequences to those identified as functionally involved in oestrogen metabolism within other bacteria. One of the sequences (JO861_08165; Table 3) was not included in the phylogenetic analyses as it was contained within the gene cluster of genes in the 9, 10-*seco* pathway. The sequences were within distinct clades with the sequences from other oestrogen degrading bacteria, thus supporting our hypothesis that they may function in E2 metabolism. The gene encoding 17β-hydroxysteroid dehydrogenase may be responsible for the production of the E1 metabolite during E2 metabolism by *R. equi* ATCC13557. E1, as a starting carbon substrate or as a metabolite, was not observed to degrade further despite the presence of genes phylogenetically related to *oecA*, *oecB*, and *oecC*. It is not clear if this was due to catabolite repression in favour of E2, or that these genes are not functionally active. Further transcriptomic and enzyme expression would be needed to determine this.

The presence of an unknown metabolite peak in the E2-only condition, and the lack of equimolar conversion of E2 to E1, suggests that there were other E2 metabolites produced. Thus there may be more than one initial step in E2 metabolism by *R. equi* ATCC13557, the occurrence of two possible initial steps has been reported previously for *Sphingomonas* sp. ED8 (Kurisu et al., 2010). Although these metabolites cannot be identified without further metabolite analyses, we can assume that based on the other two pathways for E2, which are the conversion of E2 to keto-E2 by the action of hydroxylase or conversion to 4-OH-E2 by dioxygenase (Table 1; Kurisu et al., 2010), the genes coding for catechol 2,3-dioxygenase or 3-(3-hydroxy-phenyl) propionate hydroxylase, may function in oestrogen degradation by *R. equi* ATCC13557. The hydroxylation of the A ring is suggested to be the most common first step in oestrogen degradation (Sih et al., 1965; Coombe et al., 1966; Yu et al., 2016; Chen et al., 2017) and supported by the EAWAG Biocatalysis/Biodegradation database pathway prediction (EAWAG, 2011). Additionally, the function of 3-oxosteroid 1-dehydrogenase is unclear in the degradation of E2, but in the degradation of testosterone it functions to assist in the cleavage of the A ring by the dehydrogenation leading to a double bond, however, the double bond already exists within the aromatic A ring of both E1 and E2 (Guevara et al., 2017). The gene encoding extradiol dioxygenase may function in the conversion of E2 to the unknown metabolite, possibly 4-OH-E2, or if similar to the hydroxylation of E1 to produce 4-OH-E1, by cytochrome P450 monooxygenase, which was confirmed to be common in bacterial and human metabolism of oestrogen, according to PHARMGKB oestrogen metabolism pathway. Cytochrome P450 monooxygenase may therefore be responsible for conversion of E2 to 4-OH-E2, additionally, a gene encoding cytochrome P450 monooxygenase in the genome of *Novosphingobium* sp. ES2-1 was expressed in the presence of E2 (Whirl-Carrillo et al., 2012; Hsiao et al., 2020; Ibero et al., 2020; Li et al., 2021). However, gene expression or gene knockout experiments are required to provide conclusive evidence that these genes are responsible for the metabolism of E2 by *R. equi* ATCC13557.

In this study, a gene cluster was identified within the genome of *R. equi* ATCC13557, containing 3-carboxyethylcatechol 2,3-dioxygenase (JO861_14995), 2-hydroxymuconic semialdehyde hydrolase (JO861_15000), 3-alpha-(or 20-beta)-hydroxysteroid dehydrogenase (JO861_15020), 3-(3-hydroxy-phenyl)propionate hydroxylase (JO861_15005), cytochrome P450 monooxygenase (JO861_14990), and 3-oxosteroid 1-dehydrogenase (JO861_15025). It is unclear if this cluster is involved in steroid degradation in general or oestrogen degradation specifically. The presence of oestrogen degradation encoding genes within clusters in the genomes of *R. equi* ATCC13557, *Sphingomonas* strain KC8, *N. tardaugens*, and *A. estronivorus* (Chen et al., 2017) with the presence of dioxygenase encoding genes in common (Table 4), may suggest that these bacteria share a similar oestrogen degradation pathways. However, not all oestrogen degrading bacteria harbour such gene clusters. The genome of *S. maltophilia* SJTL3 did not contain gene clusters despite, also being capable of degrading E1 and E2 like *Sphingomonas* strain KC8, *N. tardaugens*, and *A. estronivorus* (Chen et al., 2017). It may therefore use a different mechanism of oestrogen degradation (Xiong et al., 2020).

**TABLE 4:** Genes encoding oestrogen degradation enzymes found within the genomes of known oestrogen degrading bacteria.

Enzyme	Names of bacterial genomes containing genes	Number of degraders known to have the gene	References
Dioxygenase	*R. equi* ATCC13557, *P. putida* SJTE-1, *Sphingomonas* strain KC8, *Stenotrophomonas maltophilia* SJTL3, *Acinetobacter* sp. DSSKY-A-001, *Lysinibacillus sphaericus* DH-B01, *Deinococcus actinosclerus* SJTR1, *Novosphingobium tardaugens, N. tardaugens* NBRC16725, *Rhodococcus* sp. strain B50, *Rhodococcus equi* DSSKP-R-001, and *Altererythrobacter estronivorus*	12	Hu et al., 2011; Liang et al., 2012; Chen et al., 2017; Xiong et al., 2018, 2020; Zhao et al., 2018; Qiu et al., 2019; Hsiao et al., 2020; Ibero et al., 2020; Tian et al., 2020; Wang Y. et al., 2020
Hydrolase	*R. equi* ATCC13557, *P. putida* SJTE-1, *Sphingomonas* strain KC8, *Lysinibacillus sphaericus* DH-B01, *N. tardaugens* NBRC16725	5	Hu et al., 2011; Liang et al., 2012; Ibero et al., 2020; Wang Y. et al., 2020
Short-chain dehydrogenase (17β-hydroxysteroid dehydrogenase)	*R. equi* ATCC13557, *P. putida* SJTE-1, *Sphingomonas* strain KC8, *Stenotrophomonas maltophilia* SJTL3, *Rhodococcus* sp. P14, *Acinetobacter* sp. DSSKY-A-001, *Lysinibacillus sphaericus* DH-B01, *Rhodococcus* sp. strain BH2-1, *Deinococcus actinosclerus* SJTR1, *Rhodococcus equi* DSSKP-R-001, *Novosphingobium* sp. ES2-1, and *N. tardaugens* NBRC16725	12	Hu et al., 2011; Liang et al., 2012; Zhang Y. et al., 2012; Ye et al., 2017; Xiong et al., 2018, 2020; Zhao et al., 2018; Qiu et al., 2019; Ibero et al., 2020; Pratush et al., 2020; Tian et al., 2020; Wang Y. et al., 2020; Li et al., 2021
Hydroxylase	*R. equi* ATCC13557, *Acinetobacter* sp. DSSKY-A-001, *Rhodococcus* sp. strain BH2-1; *Rhodococcus equi* DSSKP-R-001	4	Zhao et al., 2018; Qiu et al., 2019; Pratush et al., 2020; Tian et al., 2020
3-oxosteroid 1-dehydrogenase	*R. equi* ATCC13557, and *Sphingomonas* strain KC8	2	Hu et al., 2011
Hydratase	*R. equi* ATCC13557, *P. putida* SJTE-1, *Sphingomonas* strain KC8, *Acinetobacter* sp. DSSKY-A-001, *Lysinibacillus sphaericus* DH-B01, *N. tardaugens* NBRC16725, and *Novosphingobium* sp. ES2-1	7	Hu et al., 2011; Liang et al., 2012; Qiu et al., 2019; Wang Y. et al., 2020; Ibero et al., 2020; Li et al., 2021
Isomerase	*R. equi* ATCC13557, *P. putida* SJTE-1, *Sphingomonas* strain KC8, *Stenotrophomonas maltophilia* SJTL3, *Rhodococcus* sp. strain BH2-1, and *N. tardaugens* NBRC16725, and *Novosphingobium* sp. ES2-1	7	Hu et al., 2011; Liang et al., 2012; Ibero et al., 2020; Pratush et al., 2020; Xiong et al., 2020; Li et al., 2021
Enoyl CoA hydratase/acetyl CoA/acyl CoA dehydrogenase	*R. equi* ATCC13557, *Sphingomonas* strain KC8. *Stenotrophomonas maltophilia* SJTL3, *Acinetobacter* sp. DSSKY-A-001, and *N. tardaugens* NBRC16725	5	Hu et al., 2011; Qiu et al., 2019; Ibero et al., 2020; Xiong et al., 2020
Monooxygenase	*Sphingomonas* strain KC8, *Stenotrophomonas maltophilia* SJTL3, *Lysinibacillus sphaericus* DH-B01, *Rhodococcus* sp. strain BH2-1; *N. tardaugens* NBRC16725, *Rhodococcus* sp. strain B50; *Rhodococcus equi* DSSKP-R-001; and *Novosphingobium* sp. ES2-1	8	Hu et al., 2011; Zhao et al., 2018; Hsiao et al., 2020; Ibero et al., 2020; Tian et al., 2020; Wang Y. et al., 2020; Xiong et al., 2020; Li et al., 2021

Although the genomes of some oestrogen degrading bacteria were not included within the genome comparison, it is important to note the genes encoding oestrogen degradation enzymes and their possible degradation function. These bacteria include the E2 degrader *Acinetobacter* sp. DSSKY-A-001, in which similar genes were identified including phenol hydroxylase, alkane oxygenase, short-chain dehydrogenase, catechol 2,3-dioxygenase, acetyl-CoA C-acetyltransferase, and enoyl-CoA hydratase (Qiu et al., 2019). Hydroquinone 1,2-dioxygenase, dioxygenase, and 7α-hydroxysteroid dehydrogenase were identified within the genome of *Acinetobacter* sp. DSSKY-A-001 and these were expressed in the presence of E2 relative to a control, the dehydrogenase likely converting E2 to E1 and dioxygenase carrying out oxygenation to further metabolise E1 (Qiu et al., 2019). The degradation pathway of *L. sphaericus* DH-B01 was postulated in which the enzyme dehydrogenase was suggested to be responsible for the conversion of E2 to E1, dioxygenase for A ring cleavage, oxygenase may be responsible for the conversion of 4-hydroxyl-E1 and B ring cleavage (Wang Y. et al., 2020). *S. maltophilia* SJTL3 also contained genes coding for the oestrogen degradation enzymes, dioxygenase, 3/17β-hydroxysteroid dehydrogenase, acyl-CoA dehydrogenase, and alcohol dehydrogenase these are likely to carry out the oxidation of the A ring, dehydrogenation of the D ring, degradation of E1 by the lactone pathway, and ketonisation, respectively (Lee and Liu, 2002; Kurisu et al., 2010; Yu et al., 2016; Xiong et al., 2020). *Rhodococcus* sp. strain BH2-1 possesses the genes coding for; cholesterol oxidase that oxidises 3β-hydroxyl group within the B ring in the first step of cholesterol degradation, steroid delta-isomerase responsible for the production of the intermediate metabolite 3-hydroxyandrosta-5,7,9(11)-trien-17-one by rearrangement of the covalently bonded carbon atoms, cytochrome P450 responsible for hydroxylation of the A ring, 3α-hydroxysteroid dehydrogenase catalyses the redox reaction of the hydroxyl ketone group within the D ring, 3δ-steroid-1-dehydrogenase and 3α,20-β-hydroxysteroid steroid dehydrogenase which carries out dehydrogenation, and 3-steroid-9α-hydroxylase (KSH), responsible for hydroxylation to open the ring structures (Pratush et al., 2020).

Additionally, *R. equi* ATCC13557 has a complete set of cholesterol and androgen degradation genes from the 9, 10-*seco* pathway (Sih et al., 1965; Holert et al., 2018). Therefore, in addition to biotransformation of E2, *R. equi* ATCC13557 has the potential to metabolise steroid hormones such as testosterone and cholesterol *via* the 9, 10-*seco* pathway, like other rhodococci (Van der Geize et al., 2007, 2011; Rosłoniec et al., 2009; Hsiao et al., 2021). But further biodegradation experiments are required to confirm this hypothesis. Bacteria biotransform steroid hormones divergently for the A/B rings, except for the function of 17β-HSD having a function in both oestrogen and testosterone metabolism (Chen et al., 2017; Chiang et al., 2019; Wu et al., 2019). The degradation pathway of all steroid hormones, including cholesterol, converges at the production of the metabolite HIP in which further metabolism of the C/D ring takes place (Chen et al., 2017; Chiang et al., 2019; Wu et al., 2019). Further, genes involved in cholesterol degradation for example *hsaC*, in *R. equi* DSSKP-R-001 were upregulated in the presence of E2, although they are not functionally validated (Zhao et al., 2018; Tian et al., 2020).

## Conclusion

In conclusion, biodegradation experiments revealed the metabolism of E2 by *R. equi* ATCC13557, with the major metabolite being E1. Analysis of the whole genome sequence of *R. equi* ATCC13557 revealed a gene cluster containing genes encoding potential oestrogen degradation enzymes, which are present in the genomes of other oestrogen degraders. The genomic evaluation also identified the presence of amino acids with similarity to the genes *oecA, oecB*, and *oecC*, previously identified in the genome of the oestrogen degrading bacterium *Sphingomonas* strain KC8 (Chen et al., 2017). Although gene expression experiments are required to measure gene expression in the presence of E2, it is possible to postulate that the pathway of E2 metabolism by *R. equi* ATCC13557 may follow two simultaneous pathways due to the production of an unknown metabolite, including the production of E1 *via* the 4, 5-*seco* pathway. Further, *R. equi* ATCC13557 may metabolise steroid hormones *via* the 9, 10-*seco* pathway.

## Data Availability Statement

The datasets presented in this study can be found in online repositories. The names of the repository/repositories and accession number(s) can be found below: https://www.ncbi.nlm.nih.gov/genbank/, JAFFSZ000000000.

## Author Contributions

The genomic DNA extraction and sequencing of *R. equi* ATCC13557 was carried out by LE and MS, who provided the raw sequencing data for this research project. The genome assembly, mapping annotation, and evaluations were carried out by SH-F. All experiments were carried out by SH-F and WM carried out uHPLC sample analysis. The project was supervised by RD, JD, PM, and WM. SH-F drafted the research paper with significant contributions from RD, JD, PM, and WM. RD concieved the research idea and project.

## Conflict of Interest

The authors declare that the research was conducted in the absence of any commercial or financial relationships that could be construed as a potential conflict of interest.

## Supplementary Material

Supplementary Figure S1 The standard curve for E1 used to determine the concentration of the E1 in the samples of the biodegradation experiment. Error bars represent the standard deviation.

Supplementary Figure S2 The standard curve for E2 used to determine the concentration of the E2 samples of the biodegradation experiment. Error bars represent the standard deviation.

Supplementary Figure S3 The standard curve for EE2 used to determine the concentration of the EE2 samples of the biodegradation experiment. Error bars represent the standard deviation.

Supplementary Figure S4 The average concentrations of estrogens measured in the 0.1% formalin with E1, E2, and EE2 abiotic control. Error bars represent the standard deviation.

Supplementary Figure S5 The average growth of *R. equi* ATCC13557 under different experimental conditions. Error bars represent the standard deviation.

Supplementary Figure S6 The average concentrations of estrogens measured mainly between 16 to 24.5 hours, in mixed conditions E1, E2, and EE2. Error bars represent the standard deviation.

Supplementary Figure S7 Unknown metabolite peak (red) present at 16 hours at retention time 1.425 min and with a peak area of 50.196nA*min.

Supplementary Figure S8 Peak area of an unknown metabolite at retention time 1.425 min in the E2 only conditions.

Supplementary Table S1 List of known oestrogen degrading bacteria isolated from activated sludge.

Supplementary Table S2 List of known oestrogen degrading bacteria from other sources.

Supplementary Table S3 The whole genomes of 16 oestrogen degrading bacteria.

Supplementary Table S4 Database of potential oestrogen genes.

Supplementary Table S5 Dehydrogenase gene sequences database.

Supplementary Table S6 Dehydrogenase gene sequences taken from Kisiela et al. (2012).

Supplementary Data Sheet 1 List of RAST annotations of the *R. equi* ATCC13557 genome.
