# Supplementary material for: Experimental and Genomic Evaluation of the Oestrogen Degrading Bacterium Rhodococcus equi ATCC13557
Source: Front Microbiol. 2021 Jul 1;12:670928. doi: 10.3389/fmicb.2021.670928 (PMC8281962; doi:10.3389/fmicb.2021.670928)
Supplement: Supplementary Figure S1 — The standard curve for E1 used to determine the concentration of the E1 in the samples of the biodegradation experiment. Error bars represent the standard deviation. [file Data_Sheet_2.docx]

Supplementary Information

This file contains:

Supplementary Figure 1: The standard curve for E1 used to determine the concentration of the E1 in the samples of the biodegradation experiment. Error bars represent the standard deviation. 2

Supplementary Figure 2: The standard curve for E2 used to determine the concentration of the E2 samples of the biodegradation experiment. Error bars represent the standard deviation. 2

Supplementary Figure 3: The standard curve for EE2 used to determine the concentration of the EE2 samples of the biodegradation experiment. Error bars represent the standard deviation. 3

Supplementary Figure 4: The average concentrations of estrogens measured in the 0.1% formalin with E1, E2, and EE2 abiotic control. Error bars represent the standard deviation. 3

Supplementary Figure 5: The average growth of *R. equi* ATCC13557 under different experimental conditions. Error bars represent the standard deviation 4

Supplementary Figure 6: The average concentrations of estrogens measured mainly between 16 to 24.5 hours, in mixed conditions E1, E2, and EE2. Error bars represent the standard deviation. 4

Supplementary Figure 7: Unknown metabolite peak (red) present at 16 hours at retention time 1.425 min and with a peak area of 50.196nA*min 5

Supplementary Figure 8: Peak area of an unknown metabolite at retention time 1.425 min in the E2 only conditions 5

Supplementary Table 1: List of known estrogen degrading bacteria isolated from activated sludge 6

Supplementary Table 2: List of known estrogen degrading bacteria from other sources 10

Supplementary Table 3: The whole genomes of 16 estrogen degrading bacteria 14

Supplementary Table 4: Database of potential estrogen genes 15

Supplementary Table 5: Dehydrogenase gene sequences database 67

Supplementary Table 6: Dehydrogenase gene sequences taken from Kisiela et al., (2012). 76

Supplementry Material References List ……………………………………………………..89

Supplementary Figure 1: The standard curve for E1 used to determine the concentration of the E1 in the samples of the biodegradation experiment. Error bars represent the standard deviation.

Supplementary Figure 2: The standard curve for E2 used to determine the concentration of the E2 samples of the biodegradation experiment. Error bars represent the standard deviation.

Supplementary Figure 3: The standard curve for EE2 used to determine the concentration of the EE2 samples of the biodegradation experiment. Error bars represent the standard deviation.


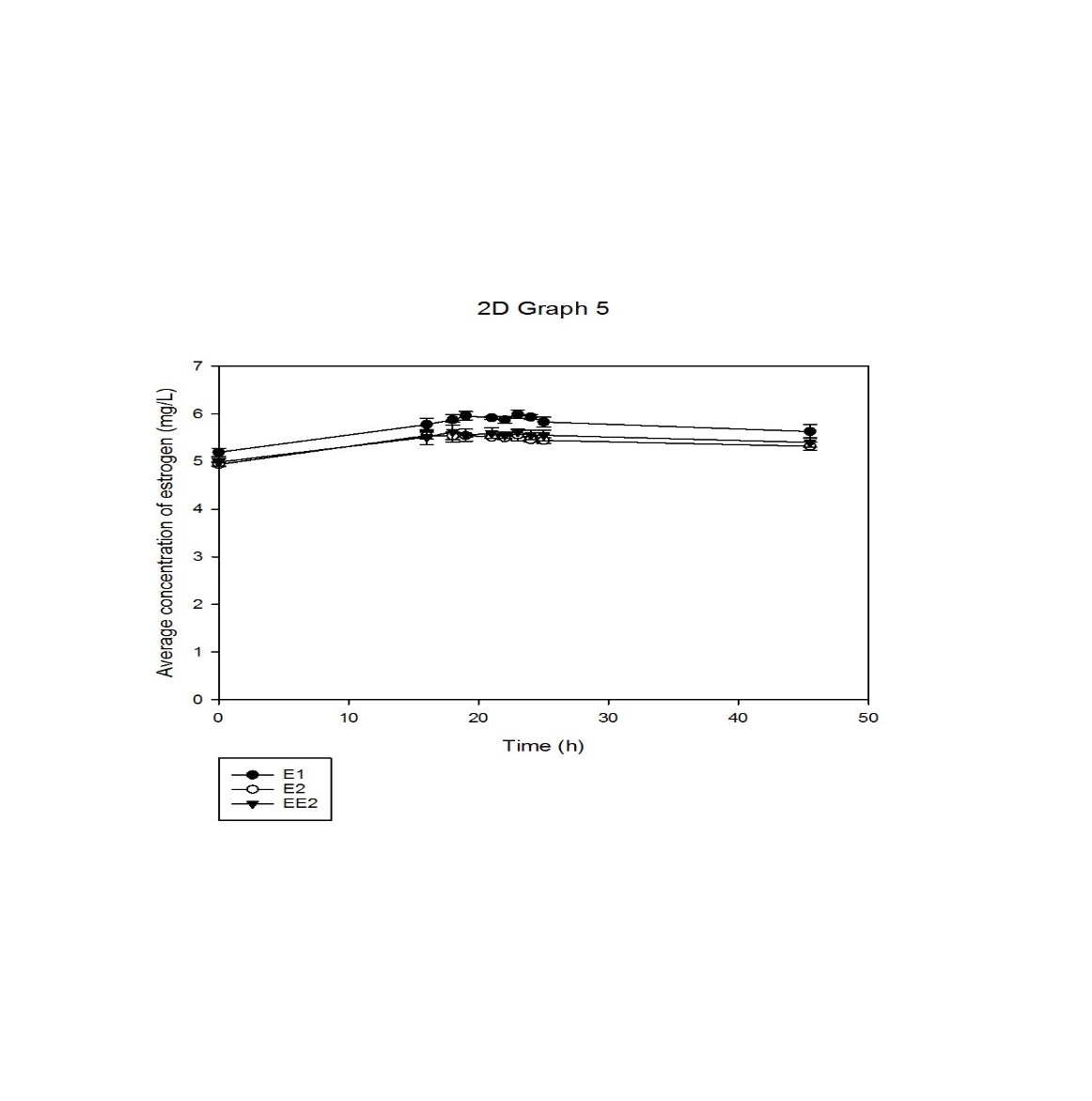


Supplementary Figure 4: The average concentrations of estrogens measured in the 0.1% formalin with E1, E2, and EE2 abiotic control. Error bars represent the standard deviation.


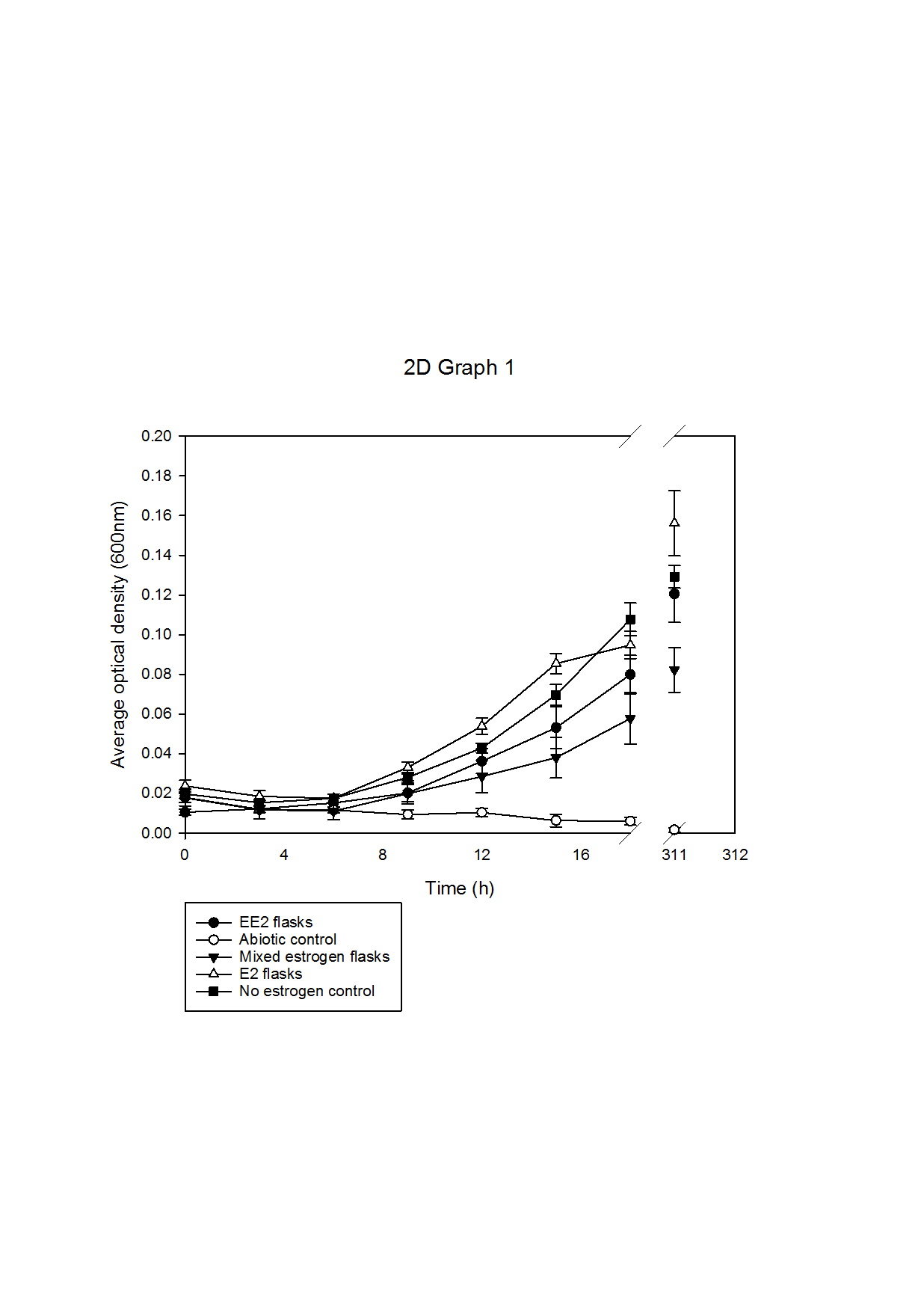


Supplementary Figure 5: The average growth of *R. equi* ATCC13557 under different experimental conditions. Error bars represent the standard deviation.


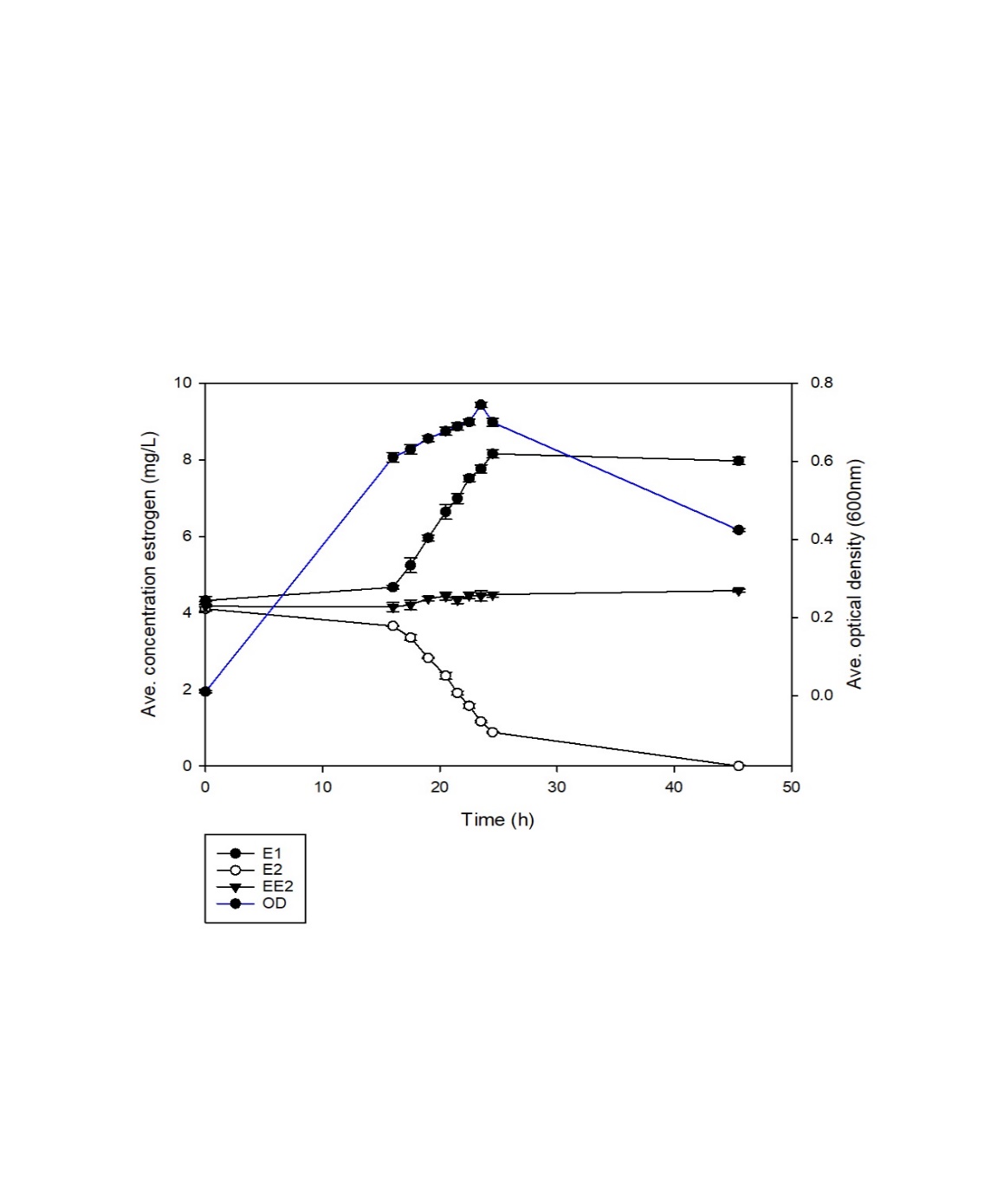


Supplementary Figure 6: The average concentrations of estrogens measured mainly between 16 to 24.5 hours, in mixed conditions E1, E2, and EE2. Error bars represent the standard deviation.

**
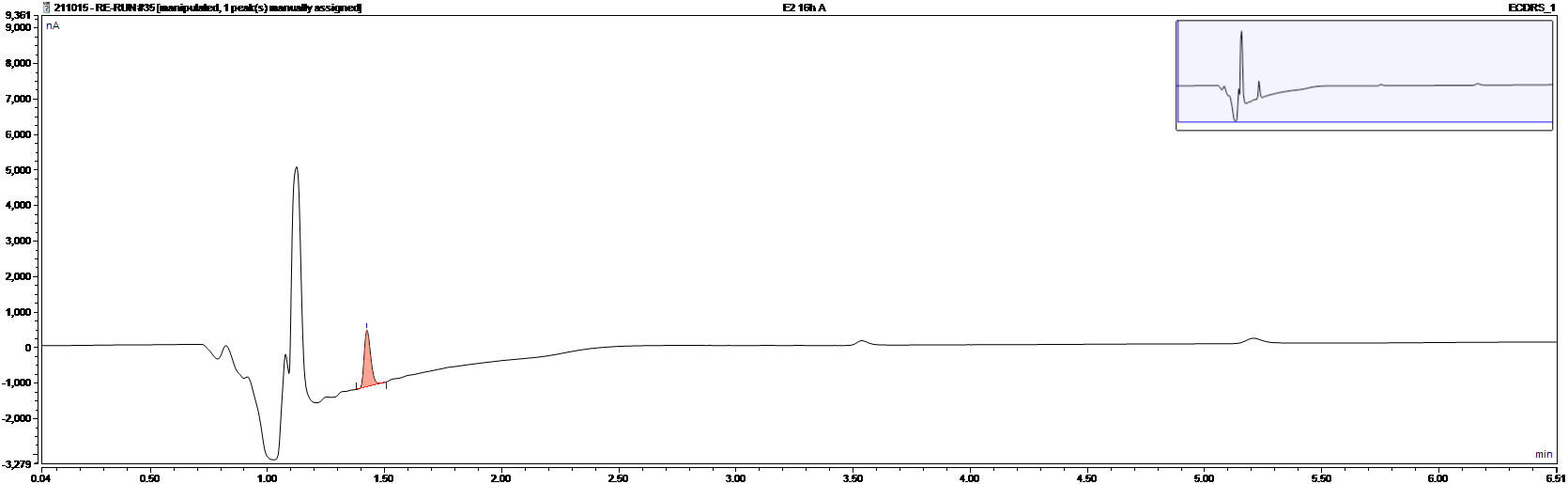
**

Supplementary Figure 7: Unknown metabolite peak (red) present at 16 hours at retention time 1.425 min and with a peak area of 50.196nA*min.

**
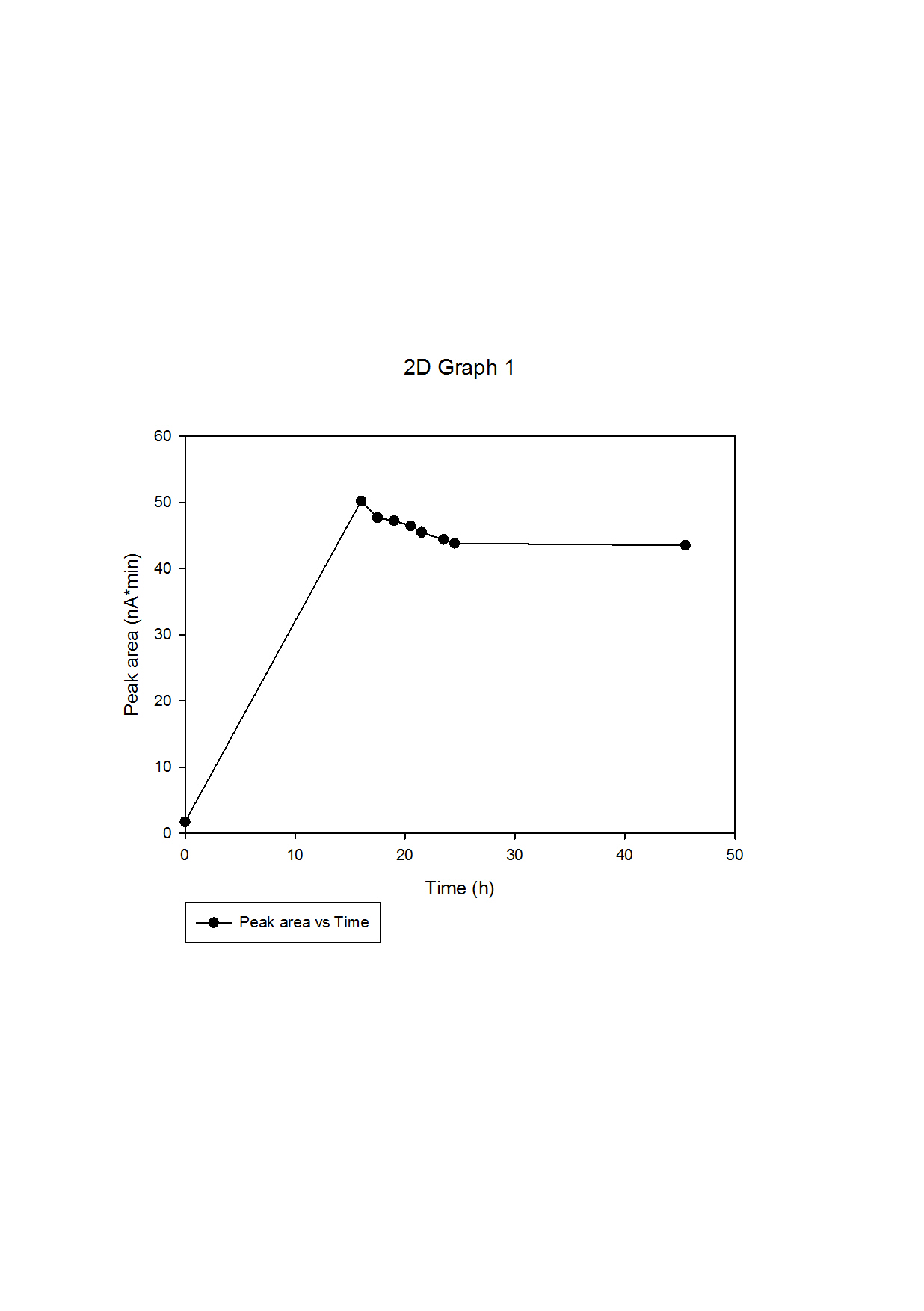
**

Supplementary Figure 8: Peak area of an unknown metabolite at retention time 1.425 min in the E2 only conditions.

Supplementary Table 1: List of known estrogen degrading bacteria isolated from activated sludge

| Bacterial species | Degradation of estrogen | Starting concentration of estrogen (mg/L) | Estrogen removal (%) | References |
| --- | --- | --- | --- | --- |
| *Novosphingobium tardaugens* NBRC 16725 (formerly sp. ARI-1) | Metabolises E1, E2, and E3. | 0.3 of E1, E2 and E3 | 40% E1 in 20 d; 99% E3 in 10 d; 60% E2 in 14 d. | Fujii et al., 2002; Fujii et al., 2003; Ibero et al., 2020. |
| *Aminobacter aminovorans* sp. KC7 | Degrades* E1 and E2. | 3 of E2 | Transformed E2 to E1 in 7 d; 23-31% E1 removed in 7 d. | Yu et al., 2007. |
| *Aminobacter* sp. KC6 | Degrades* E1 and E2. | 3 of E2 | Transformed E2 to E1 in 7 d; 16-26% E1 degraded in 7 d. | Yu et al., 2007. |
| *Sphingomonas* strain KC8 | Metabolises E1 and E2. | 3 of E2 | Complete mineralization of E1 and E2 in 7 d. | Roh and Chu, 2010; Yu et al., 2007; Yu et al., 2013. |
| *Brevundimonas diminuta* strain NK 2 (EU35276) | Transforms E2 to E1 | 3- 4 of E2 | The residual concentration of E2 was 38 ± 1% after 168 h of incubation. | Muller et al., 2010. |
| *Brevundimonas vesicularis* KC12 | Transforms E2 to E1 | 3 of E2 | Complete transformation within 7 days. | Yu et al., 2007. |
| *Rhodococcus ruber* M2 KC4 | Transforms E2 to E1 | 3 of E2 | Complete transformation within 1 day. | Yu et al., 2007. |
| *Microbacterium testaceum* KC5. | Transforms E2 to E1 | 3 of E2 | Complete transformation within 1 day. | Yu et al., 2007. |
| *Sphingomonas* sp. KC9-11 | Transforms E2 to E1 | 3 of E2 | Complete transformation within 1 day. | Yu et al., 2007. |
| *Flavobacterium* sp. KC1 | Transforms E2 to E1 | 3 of E2 | Complete transformation within 7 days. | Yu et al., 2007. |
| Bacteroidetes sp. KC2 | Transforms E2 to E1 | 3 of E2 | Complete transformation within 7 days. | Yu et al., 2007. |
| *Nocardioides simplex* sp. KC3 | Transforms E2 to E1 | 3 of E2 | Complete transformation within 7 days. | Yu et al., 2007. |
| *Escherichia coli* sp. KC13 | Transforms E2 to E1 | 3 of E2 | Complete transformation within 7 days. | Yu et al., 2007. |
| *Sphingomonas* sp. KC14 | Transforms E2 to E1 | 3 of E2 | Complete transformation within 7 days. | Yu et al., 2007. |
| *Achromobacter xylosoxidans* | Degrades* E1, E2, and transforms E3. | 0.1- 5.5 | Complete transformation within 2 d. | Weber et al., 2005. |
| *Ralstonia* sp. | Degrades* E1, E2, and transforms E3. | 0.1 – 5.5 | Complete transformation within 2 d. | Weber et al., 2005. |
| *Pseudomonas aeruginosa* sp. TJ1 | Metabolises E2. | 5 x 10^-3^ – 1.5 x 10^-2^ | Complete mineralization of E2 in 2 h. | Zeng et al., 2009. |
| *Bacillus subtilis* sp. E2Y4 | Degrades* E1 and E2. | 10 | Transformed E2 completely in 4 days; 40% E1 within 9 d. | Jiang et al., 2010. |
| *Bacillus* sp. E2Y1 | Degrades* E1 and E2. | 10 | Transformed E2 completely in 6 days; 20% E1 within 9 d. | Jiang et al., 2010. |
| *Bacillus amylonquefaciens* sp. E2Y2 | Transforms E2 to E1. | 10 | Transformed E2 to E1 within 4 days. | Jiang et al., 2010. |
| *Bacillus* sp. E2Y3 | Transforms E2 to E1. | 10 | Transformed E2 to E1 within 5 days. | Jiang et al., 2010. |
| *Bacillus cereus* sp. E2Y5 | Transforms E2 to E1. | 10 | Transformed E2 to E1 within 6 days. | Jiang et al., 2010. |
| *Denitratisoma oestradiolicum* gen. nov., sp. nov | Metabolises E2 under anoxic conditions. | 0.3 | Complete mineralization of E2. | Fahrbach et al., 2006. |
| *Steroidobacter denitrificans* gen. nov., sp. nov | Metabolises E2 under anoxic conditions. | 0.3 | Complete mineralization of E2. | Fahrbach et al., 2008. |
| *Nitrosomonas europaea* | Co-metabolises E1, E2, E3, and EE2 under nitrifying conditions. | 1 | >95-100% E1, E2 and EE2 in 8 days; >62.5% E3 in 9 d. | Shi et al., 2004. |
| *Sphingobacterium* sp. JCR5 | Metabolises E1, E2, E3, and EE2. | 30 | 87% EE2 within 10 d. | Haiyan et al., 2007. |
| *Novosphingobium* sp. JEM-1 | Degrades* E1, E2, and EE2. | 4.5 x10^-5^ ± 4.8 x 10^-6^ of E1 and 2.0x10^-5^ ± 3.0 x10^-6^ of E2 | 99-100% E1 and E2 in <1 h. | Hashimoto et al., 2010. |
| *Rhodococcus zopfii* strain Y 50158 | Metabolises E1, E2, E3, and EE2. | 100 | 81% E2 in 2 h; 91% E1 in 3 h; 96% E3 in 4 h; 70% EE2 in 7 h; Complete mineralisation in 24 h. | Yoshimoto et al., 2004. |
| *Rhodococcus equi* strains Y 50155, Y 50156, and Y 50157 | Metabolises E1, E2, E3, and EE2. | 100 | >80% E2 in 5 h; 99% E2 in 24 h; >80% E1 in 8 h; 99% in 24 h; 72-95% E3 in 24 h; >70% EE2 in 8 h; >80-95% in 24 h. | Yoshimoto et al., 2004. |
| *Pseudomonas citronellolis* SS-2 | Degradation of E1, E2, and EE2, but not E3 | 4 of EE2  2 of E2 | 93.6% EE2 removal in 168 hours.  99% E2 in 36 hours. | Zhang et al., 2012b |
| *Pseudomonas citronellolis* SJTE-3 | Metabolises E1, E2, E3, and EE2. | Unpublished data | Unpublished data | Zheng et al., 2016. |
| *Novosphingobium* sp. E2S | Degradation of E2 | 50 | 63.29% of E2 was removed within 7 days. | Li et al., 2017 |
| *Novosphingobium* sp. ES2-1 | Degradation of E1 and E2 | 10 | 97.1% of E2 was removed within 7 days and | Li et al., 2020 |
| *Rhodococcus* sp. DS201 | Metabolises E2 and E1 | 0.5 – 10 | 63.4-100% E2 removed in 3 days, highest removal 100% of 1 mg/L E2 in 3 days. Lowest 63.4% removal of 10mg/L E2 in 3 days. | Yu et al., 2016 |
| *Deinococcus actinosclerus* SJTR1 | Transforms E2 to E1 | 10 | 90% E2 removal in 5 days. | Xiong et al., 2018 |
| *Denitratisoma* sp. strain DHT3 | Metabolises E2 under denitrifying anaerobic conditions | 500 | 500 mg consumed in 72 hours. | Wang et al., 2020b |
| *Stenotrophomonas maltophilia SJTL3* | Metabolises E2 | 0.001  0.01  0.025 | 95% E2 removal in 6 days.  90% E2 removal in 14 days.  78% E2 removal in 14 days. | Xiong et al., 2020 |
| *Sphingobium estronivorans* sp. nov. | Metabolises E1 and E2 | 2 | Using cell immobilization technique, 94% E2 under 12 h hydraulic retention time (HRT). | Qin et al., 2020 and Ma et al., 2016 |
| *Sphingobium bisphenolivorans* sp. nov | Metabolises E1 and E2 | 2 | Using cell immobilization technique, 94% E2 under 12 h hydraulic retention time (HRT). | Qin et al., 2020 and Ma et al., 2016 |
| *Novosphingobium* sp. strain SLCC | Metabolises E1 and E2, not EE2. | 0.001 | Metabolites detected after 24 hours. | Chen et al., 2018 |
| *Lysinibacillus sphaericus* DH‑B01 | Metabolises E2 | 30 | >90% E2 removal in 72 hours. | Wang et al., 2020a |

* Where strains were unable to grow upon estrogen as the sole energy or carbon source, but co-metabolism was not confirmed. All strains are aerobic unless otherwise stated.

Supplementary Table 2: List of known estrogen degrading bacteria from other sources

| Bacterial species | Degradation of estrogen | Source of bacteria | References |
| --- | --- | --- | --- |
| *Phyllobacterium myrsinacearum* | Metabolises E1, E2, E3, and co-metabolizes EE2 in the presence of E1, E2, and E3. | Compost | Pauwels et al., (2008) |
| *Ralstonia picketti* BP2 | Metabolises E1, E2, E3, and co-metabolizes EE2 in the presence of E1, E2, and E3. | Compost | Pauwels et al., (2008) |
| *Pseudomonas aeruginosa* BP3 | Metabolises E1, E2, E3, and co-metabolizes EE2 in the presence of E1, E2, and E3. | Compost | Pauwels et al., (2008) |
| *Pseudomonas* sp. BP7 | Metabolises E1, E2, E3, and co-metabolizes EE2 in the presence of E1, E2, and E3. | Compost | Pauwels et al.,(2008) |
| *Acinetobacter* sp. | Metabolises E1, E2, E3, and co-metabolizes EE2 in the presence of E1, E2, and E3. | Compost | Pauwels et al., (2008) |
| *Sphingomonas* sp. CYH | Degrades E1 (aerobic and anoxic) and transforms E2 to E1. | Artificial sandy aquifer | Ke et al., (2007) |
| *Agromyces* sp. LHJ3 | Degrades E3 (aerobic), E2 (anoxic), and transforms E2 to E1. | Artificial sandy aquifer | Ke et al., (2007) |
| *Acinetobacter* sp. LHJ1 | Transforms E2 to E1. | Artificial sandy aquifer | Ke et al., (2007) |
| *Sphingomonas* ED8 and 9. | Metabolises E1 and E2. | Soil | Kurisu et al., (2010) |
| *Rhodococcus* ED6, 7, and 10. | Metabolises E1 and E2. | Soil | Kurisu et al., (2010) |
| *Pseudomonas putida* strain SJTE-1 | Metabolises E1 and E2 | Soil | Liang et al., (2012) |
| *Leptothrix discophora* | Manganese Mn^2+^-  dependent degradation of EE2 | Belgian coordinated  collections of  Microorganisms. | Sabirova et al., (2008) |
| *Pseudomonas putida* MnB1, 6 and 29. | Manganese Mn^2+^-  dependent degradation of EE2 | Belgian coordinated  collections of  Microorganisms. | Sabirova et al., (2008) |
| *Nitrosomonas europaea* ATCC 19718 | Co-metabolism and Nitration of EE2 | ATCC | Gaulke et al., (2008);  Skotnicka-Pitak et al.,  (2009) |
| *Buttiauxella* | Metabolism of E2 and Testosterone | Baltic Sea | Zhang et al., (2011) |
| *Vibrio* sp. H5 | Metabolism of E2 and Testosterone | Baltic Sea | Sang et al., (2012) |
| *Rhodococcus equi* ATCC 13557 | Partial degradation of EE2 in the presence of a cosubstrate. EE2 61% ± 1% in 300h. | ATCC | O’Grady et al., (2009); Larcher and Yargeau (2013) |
| *Rhodococcus erythropolis* ATCC 4277 | Partial degradation of EE2 in the presence of a cosubstrate. EE2 46% ± 2% in 300h | ATCC | O’Grady et al., (2009 ); Larcher and Yargeau (2013) |
| *Rhodococcus zopfii* ATCC 51349 | Partial degradation of EE2 in the presence of a cosubstrate. EE2 38% ± 1% in 300h | ATCC | O’Grady et al., (2009); Larcher and Yargeau (2013) |
| *Bacillus subtilis* ATCC 6051 | Partial degradation of EE2 in the presence of a cosubstrate. EE2 27% ± 2% in 300h. | ATCC | Larcher and Yargeau (2013) |
| *Pseudomonas aeruginosa PA01* | Partial degradation of EE2 in the presence of a cosubstrate. EE2 34% ± 2% in 300h. | ATCC | Larcher and Yargeau (2013) |
| *Pseudomonas putida ATCC12633* | Partial degradation of EE2 in the presence of a cosubstrate. EE2 21% ± 2% in 300h | ATCC | Larcher and Yargeau (2013) |
| *Rhodococcus rhodochrous ATCC13808* | Partial degradation of EE2 in the presence of a cosubstrate. No EE2 detected after 48 h (possibly co-metabolic) | ATCC | Larcher and Yargeau (2013) |
| *Bacillus cereus* Socransky 67 | Conversion of E2 to unknown metabolites | Dental plaque | Ojanotko-Harri et al., (1990) |
| *Streptococcus mutans* Ingbritt | Conversion of E2 to E1 | Dental plaque | Ojanotko-Harri et al., (1990) |
| *Streptococcus mutans* NCTC 10449 | Conversion of E2 to E1 | Dental plaque | Ojanotko-Harri et al., (1990) |
| *Streptococcus mutans* NCTC 10904 | Conversion of E2 to E1 | Dental plaque | Ojanotko-Harri et al., (1990) |
| *Rhodococcus* sp*.* P14 | Conversion of E2 to E1 | Crude oil | Ye et al., 2017; Song et al., 2011. |
| *Nocardia* sp. E110 | Degradation of E1 | Soil | Coombe et al., 1966 |
| *Acinetobacter* sp. DSSKY-A-001 | Degradation of E2 | Soil | Qiu et al., 2019 |
| *Altererythrobacter estronivorus* sp. nov | Degradation of E1 and E2 | Lagoon | Qin et al., 2016 |
| *Rhodococcus* sp. strain B50 | Degradation of E1, E2, E3 and testosterone | Soil | Hsiao et al., 2020; Hsiao et al., 2021 |
| *Rhodococcus* sp. strain DSSKP-R-001 | Degradation of E1, E2 and EE2 | Soil | Zhao et al., 2018; Tian et al., 2020 |
| *Rhodococcus* sp. strain BH2-1 | Degradation of E1 | Mangrove soil | Pratush et al., 2020 |

| Bacteria | Degradation of steroid hormones | Reference |
| --- | --- | --- |
| *Sphingomonas* sp. strain KC8 | E1 and E2 | Hu et al., 2011 |
| *Pseudomonas putida* strain SJTE-1 | E1 and E2 | Liang et al., 2012 |
| *Altererythrobacter estronivorus* sp. nov | E1 and E2 | Qin et al., 2016 |
| *Sphingobium estronivorans* sp. nov. | E1 and E2 | Qin et al., 2020 |
| *Sphingobium bisphenolivorans* sp. nov. | E1 and E2 | Qin et al., 2020 |
| *Stenotrophomonas maltophilia* SJTL3 | E1 and E2 | Xiong et al., 2020 |
| *Novosphingobium* sp. ES2-1 | E1 and E2 | Li et al., 2020; Li et al., 2021 |
| *Rhodococcus* sp. P14 | Transforms E2 to E1 | Ye et al., 2017; Zhang et al., 2012a |
| *Deinococcus actinosclerus* SJTR1 | Transforms E2 to E1 | Xiong et al., 2018 |
| *Pseudomonas citronellolis* SJTE-3 | E1, E2, E3 and EE2 | Zheng et al., 2016 |
| *Novosphingobium tardaugens* NBRC 16725 | Testosterone, E1, E2, and E3 | Fujii et al., 2002; Fujii et al., 2003; Ibero et al., 2019; Ibero et al., 2020 |
| *Rhodococcus equi* DSSKP-R-001 | E1, E2, and EE2 | Tian et al., 2020; Zhao et al.. 2018 |
| *Acinetobacter* sp. DSSKY-A-001 | E2 | Qiu et al., 2019 |
| *Lysinibacillus sphaericus* DH‑B01 | E2 | Wang et al., 2020a |
| *Rhodococcus* sp. strain BH2-1 | E1 | Pratush et al., 2020 |
| *Denitratisoma* sp. strain DHT3 | Anaerobic E2 degrader, and transforms E2 to androgens | Wang et al., 2020b |

Supplementary Table 3: The whole genomes of 16 estrogen degrading bacteria

Supplementary Table 4: Database of potential estrogen genes

| **Gene/enzyme** | **Accession n.** | **Description** |
| --- | --- | --- |
| ORF11 | AB063482 a | *Comamonas testosteroni* ORF11, tesA, tesD, tesE, tesF, tesG genes, complete cds |
| TesA/ORF12 (hydroxylase) | AB063482 b | *Comamonas testosteroni* ORF11, tesA, tesD, tesE, tesF, tesG genes, complete cds |
| TesA2 (hydroxylase of 3-hydroxy-9, 10-secoandrosta -1, 3, 5(10)-triene-9, 17-dione, flavin-dependent monooxygenase oxygenase subunit) | LC010134 | *Comamonas testosteroni* DNA, putative mega-cluster of steroid degradation genes, strain: T441. |
| flavin-dependent monooxygenase oxygenase subunit | CP006704 | *Comamonas testosteroni* TK102, complete genome. |
| ORF17 (3-ketosteroid-9-alpha-hydroxylase reductase) 1a (EC(1.14.13.142) | AB076368 | *Comamonas testosteroni* tesH, tesI, ORF17, ORF18 genes for 3-ketosteriod-delta1-dehydrogenase, 3-ketosteriod-delta4(5alpha)-dehydrogenase, hypothetical protein, complete cds. |
| 3-ketosteroid-9-alpha-hydroxylase reductase subunit | CP006704 | *Comamonas testosteroni* TK102, complete genome. |
| 3-(3-hydroxy-phenyl)proprionate hydroxylase | JAFFSZ010000000 | *R. equi* ATCC13557 rast annotation of spades assembly |
| Chromosome I | LN879547 | *Comamonas testosteroni* P19 genome assembly Comamonas testosteroni P19, chromosome : I |
| 3-ketosteroid 9alpha-monooxygenase (1.14.13.142) | BCMM01000037.1 | *Streptomyces scabiei* DNA, scaffold: NODE_037_cov_57.5993, strain: S58, whole genome shotgun sequence |
| 3-ketosteroid 9alpha-monooxygenase (1.14.13.142) | LLWJ01000056.1 | *Pseudomonas* sp. TAA207 contig000056, whole genome shotgun sequence |
| 3-ketosteroid 9alpha-monooxygenase (1.14.13.142) | LLWI01000067.1 | *Pseudomonas* sp. TAD18 contig000067, whole genome shotgun sequence |
| 3-ketosteroid 9alpha-monooxygenase (1.14.13.142) | BCMK01000014.1 | *Streptomyces acidiscabies* DNA, scaffold: NODE_014_cov_52.5735, strain: a10, whole genome shotgun sequence |
| 3-ketosteroid 9alpha-monooxygenase (1.14.13.142) | BCMK01000200.1 | *Streptomyces acidiscabies* DNA, scaffold: NODE_200_cov_56.898, strain: a10, whole genome shotgun sequence |
| 3-ketosteroid 9alpha-monooxygenase (1.14.13.142) | BCMN01000002.1 | *Streptomyces turgidiscabies* DNA, scaffold: NODE_002_cov_60.061, strain: T45, whole genome shotgun sequence |
| 3-ketosteroid 9alpha-monooxygenase (1.14.13.142) | CP012042.1 | *Burkholderia pseudomallei* strain Bp1651 chromosome 2, complete sequence |
| 3-ketosteroid 9alpha-monooxygenase (1.14.13.142) | CVQP01000002.1 | *Rhodococcus* sp. RD6.2 genome assembly RD6.2, scaffold RHCRD62_Contig_10, whole genome shotgun sequence |
| 3-ketosteroid 9alpha-monooxygenase (1.14.13.142) | CVQP01000005.1 | *Rhodococcus* sp. RD6.2 genome assembly RD6.2, scaffold RHCRD62_Contig_2, whole genome shotgun sequence |
| 3-ketosteroid 9alpha-monooxygenase (1.14.13.142) | CVQP01000007.1 | *Rhodococcus* sp. RD6.2 genome assembly RD6.2, scaffold RHCRD62_Contig_4, whole genome shotgun sequence |
| Rieske (2Fe-2S) domain-containing protein | CP011072.1 | *Azoarcus* sp. CIB, complete genome |
| 3-ketosteroid 9alpha-monooxygenase (1.14.13.142) | JYNL01000001.1 | *Mycobacterium chlorophenolicum* strain DSM 43826 MCHLDSM_contig000001, whole genome shotgun sequence |
| 3-ketosteroid-9-alpha-monooxygenase oxygenase subunit | JYNL01000008.1 | *Mycobacterium chlorophenolicum* strain DSM 43826 MCHLDSM_contig000008, whole genome shotgun sequence |
| 3-ketosteroid-9-alpha-monooxygenase oxygenase | JYNU01000001.1 | *Mycobacterium obuense* strain DSM 44075 MOBUDSM44075_contig000001, whole genome shotgun sequence |
| 3-ketosteroid-9-alpha-monooxygenase oxygenase | JYNU01000002.1 | *Mycobacterium obuense* strain DSM 44075 MOBUDSM44075_contig000002, whole genome shotgun sequence |
| 3-ketosteroid-9-alpha-monooxygenase oxygenase | JYNX01000035.1 | *Mycobacterium chubuense* strain DSM 44219 MCHUDSM44219_contig000035, whole genome shotgun sequence |
| 3-ketosteroid-9-alpha-monooxygenase oxygenase | JYNX01000038.1 | *Mycobacterium chubuense* strain DSM 44219 MCHUDSM44219_contig000038, whole genome shotgun sequence. |
| 3-ketosteroid-9-alpha-monooxygenase oxygenase | JYNL01000069.1 | *Mycobacterium chlorophenolicum* strain DSM 43826 MCHLDSM_contig000070, whole genome shotgun sequence |
| 3-ketosteroid-9-alpha-monooxygenase oxygenase | CDHG01000028.1 | *Mycobacterium caprae* genome assembly Genome assembly of *M caprae* MB2, contig 28, whole genome shotgun sequence |
| 3-ketosteroid-9-alpha-monooxygenase oxygenase | CDHE01000065.1 | *Mycobacterium bovis* genome assembly Assembly of *Mycobacterium bovis* MB4 genome, contig 65, whole genome shotgun sequence |
| 3-ketosteroid-9-alpha-monooxygenase oxygenase | CP006850.1 | *Nocardia nova* SH22a, complete genome |
| 3-ketosteroid-9-alpha-monooxygenase oxygenase | CDHH01000076.1 | *Mycobacterium bovis* genome assembly Assembly of the genome MB3, contig 76, whole genome shotgun sequence |
| 3-ketosteroid-9-alpha-monooxygenase oxygenase | CDHF01000008.1 | *Mycobacterium bovis* genome assembly Genome assembly of Mycobacterium bovis MB1, contig 8, whole genome shotgun sequence |
| 3-ketosteroid-9-alpha-monooxygenase oxygenase | HG322950.1 | *Pseudomonas knackmussii* B13 complete genome |
| 3-ketosteroid-9-alpha-monooxygenase oxygenase | CCSD01000032.1 | *Rhodococcus ruber* genome assembly BDK_PRJEB6917_v1, contig BDK_RHRU231_Contig_23, whole genome shotgun sequence |
| 3-ketosteroid-9-alpha-monooxygenase oxygenase | CCSD01000043.1 | *Rhodococcus ruber* genome assembly BDK_PRJEB6917_v1, contig BDK_RHRU231_Contig_33, whole genome shotgun sequence |
| 3-ketosteroid-9-alpha-monooxygenase oxygenase | CCSD01000089.1 | *Rhodococcus ruber* genome assembly BDK_PRJEB6917_v1, contig BDK_RHRU231_Contig_75, whole genome shotgun sequence |
| 3-ketosteroid-9-alpha-monooxygenase oxygenase | LN482603.1 | *Rhodococcus ruber* genome assembly BDK_PRJEB6917_v1, scaffold BDK_RHRU231_scaffold13, whole genome shotgun sequence |
| 3-ketosteroid-9-alpha-monooxygenase oxygenase | LN482615.1 | *Rhodococcus ruber* genome assembly BDK_PRJEB6917_v1, scaffold BDK_RHRU231_scaffold25, whole genome shotgun sequence |
| flavodoxin reductase family protein | JTJI01000003.1a | *Prauserella* sp. Am3 HQ32_scaffold_2.3, whole genome shotgun sequence |
| Rieske (2Fe-2S) domain-containing | JTJI01000003.1b | *Prauserella* sp. Am3 HQ32_scaffold_2.3, whole genome shotgun sequence |
| 3-ketosteroid-9-alpha-monooxygenase oxygenase | CP008953.1 | *Amycolatopsis japonica* strain MG417-CF17, complete genome |
| 3-ketosteroid-9-alpha-monooxygenase oxygenase | JNNW01000040.1 | Mycobacterium tuberculosis strain A4 Contig_136_len_305152, whole genome shotgun sequence |
| 3-ketosteroid-9-alpha-monooxygenase oxygenase | JNGF01000142.1 | *Mycobacterium tuberculosis* strain A2 Contig_56, whole genome shotgun sequence |
| 3-ketosteroid-9-alpha-monooxygenase oxygenase | AZIN01000001.1 | Gammaproteobacteria bacterium MOLA455 MOLA455_Contig1, whole genome shotgun sequence |
| putative oxidoreductase | FN554889.1a | *Streptomyces scabiei* 87.22 complete genome |
| putative oxidoreductase | FN554889.1b | *Streptomyces scabiei* 87.22 complete genome |
| rieske [2Fe-2S] domain protein | CP009160.1 | *Burkholderia pseudomallei* TSV 48 chromosome 2, complete sequence |
| Rieske (2Fe-2S) protein | CP012577.1 | *Burkholderia pseudomallei* strain 982 chromosome 2, complete sequence |
| rieske [2Fe-2S] domain protein | CP010066.1 | *Burkholderia mallei* strain 2002721276 chromosome 2, complete sequence. |
| rieske [2Fe-2S] domain protein | CP009943.1 | *Burkholderia mallei* strain KC_1092 chromosome 2 sequence. |
| rieske [2Fe-2S] domain protein | CP009898.1 | *Burkholderia pseudomallei* *Pasteur* 52237 chromosome 2, complete sequence. |
| rieske [2Fe-2S] domain protein | CP009588.1 | *Burkholderia mallei* strain 11 chromosome II, complete sequence. |
| rieske [2Fe-2S] domain protein | CP009537.1 | *Burkholderia pseudomallei* K96243 chromosome II, complete sequence. |
| rieske [2Fe-2S] domain protein | CP009484.1 | *Burkholderia pseudomallei* MSHR491 chromosome II, complete sequence. |
| rieske [2Fe-2S] domain protein | CP009338.1 | *Burkholderia mallei* strain 2002734299 chromosome 2, complete |
| rieske [2Fe-2S] domain protein | CP010974.1 | *Burkholderia pseudomallei* strain vgh07 chromosome 2, complete sequence. |
| rieske [2Fe-2S] domain protein | CP008731.2 | *Burkholderia mallei* strain 2000031063 chromosome 2, complete |
| rieske [2Fe-2S] domain protein | CP009156.1 | *Burkholderia* sp. TSV202 chromosome 2, complete sequence. |
| rieske [2Fe-2S] domain protein | CP009150.1 | *Burkholderia pseudomallei* B03 chromosome 2, complete sequence. |
| rieske [2Fe-2S] domain protein | CP009164.1 | *Burkholderia pseudomallei* A79A chromosome 2, complete sequence. |
| rieske [2Fe-2S] domain protein | CP009152.1 | *Burkholderia pseudomallei* MSHR3965 chromosome 2, complete sequence. |
| rieske [2Fe-2S] domain protein | CP004378.1 | Complete genome sequences for 59 *burkholderia* isolates, both pathogenic and near neighbor |
| rieske [2Fe-2S] domain protein | CP008917.1 | *Burkholderia* sp. BGK chromosome 2 sequence. |
| rieske [2Fe-2S] domain protein | CP007801.1 | *Burkholderia mallei* NCTC 10247 chromosome 2, complete sequence. |
| rieske [2Fe-2S] domain protein | CP009147.1 | *Burkholderia mallei* strain FMH 23344 chromosome 2, complete |
| rieske [2Fe-2S] domain protein | CP008912.1 | *Burkholderia pseudomallei* HBPUB10134a chromosome 2, complete |
| rieske [2Fe-2S] domain protein | CP008910.1 | *Burkholderia pseudomallei* MSHR5848 chromosome 2, complete sequence. |
| rieske [2Fe-2S] domain protein | CP008783.1 | *Burkholderia pseudomallei* MSHR5855 chromosome 2, complete sequence. |
| rieske [2Fe-2S] domain protein | CP008754.1 | *Burkholderia pseudomallei* strain 9 chromosome 2, complete sequence. |
| rieske [2Fe-2S] domain protein | CP008722.1 | *Burkholderia mallei* strain BMQ chromosome 2, complete sequence. |
| rieske [2Fe-2S] domain protein | CP008710.1 | *Burkholderia mallei* strain 6 chromosome 2, complete sequence. |
| rieske [2Fe-2S] domain protein | CP008705.1 | *Burkholderia mallei* strain 23344 chromosome 2, complete sequence. |
| Rieske iron-sulphur domain protein | LK936443.1 | *Burkholderia pseudomallei* genome assembly BP_3921g, chromosome : 2. |
| iron-sulfur cluster-binding protein, Rieske family | CP000547.1 | *Burkholderia mallei* NCTC 10247 chromosome II, complete sequence. |
| iron-sulfur cluster-binding protein, Rieske | CP000545.1 | *Burkholderia mallei* NCTC 10229 chromosome II, complete sequence. |
| iron-sulfur cluster-binding protein, rieske | CP000525.1 | *Burkholderia mallei* SAVP1 chromosome II, complete sequence. |
| iron-sulfur cluster-binding protein, rieske | CP000125.1 | *Burkholderia pseudomallei* 1710b chromosome II, complete sequence. |
| iron-sulfur cluster-binding protein, rieske | CP000011.2 | *Burkholderia mallei* ATCC 23344 chromosome 2, complete sequence. |
| Rieske iron-sulphur domain protein | BX571966.1 | *Burkholderia pseudomallei* strain K96243, chromosome 2, complete |
| rieske [2Fe-2S] domain protein | CP009546.1 | *Burkholderia pseudomallei* strain MSHR668 chromosome II, complete |
| rieske [2Fe-2S] domain protein | CP009536.1 | *Burkholderia pseudomallei* 7894 chromosome II, complete sequence. |
| rieske [2Fe-2S] domain protein | CP009477.1 | *Burkholderia pseudomallei* MSHR2543 chromosome II, complete |
| rieske [2Fe-2S] domain protein | CP009473.1 | *Burkholderia pseudomallei* MSHR840 chromosome II, complete sequence. |
| rieske [2Fe-2S] domain protein | CP009234.1 | *Burkholderia pseudomallei* MSHR62 chromosome 2, complete sequence. |
| rieske [2Fe-2S] domain protein | CP009269.1 | *Burkholderia pseudomallei* MSHR2243 chromosome 2 sequence. |
| rieske [2Fe-2S] domain protein | CP009272.1 | *Burkholderia pseudomallei* MSHR1153 chromosome 2 sequence |
| rieske [2Fe-2S] domain protein | CP009210.1 | *Burkholderia pseudomallei* strain BDP chromosome 2, complete |
| rieske [2Fe-2S] domain protein | CP008891.1 | *Burkholderia pseudomallei* MSHR5858 chromosome 2, complete sequence. |
| rieske [2Fe-2S] domain protein | CP008779.1 | *Burkholderia pseudomallei* strain MSHR1655 chromosome 2, complete |
| rieske [2Fe-2S] domain protein | CP004369.1 | *Burkholderia pseudomallei* MSHR520 chromosome 2, complete sequence |
| rieske [2Fe-2S] domain protein | CP003977.1 | *Burkholderia pseudomallei* NCTC 13179 chromosome 2, complete |
| rieske [2Fe-2S] domain protein | CP006469.1 | *Burkholderia pseudomallei* MSHR305 chromosome 2, complete sequence. |
| iron-sulfur cluster-binding protein, Rieske | CP000571.1 | *Burkholderia pseudomallei* 668 chromosome II, complete sequence. |
| Rieske (2Fe-2S) protein | CP012518.1 | *Burkholderia pseudomallei* strain vgh16W chromosome 2, complete |
| Rieske (2Fe-2S) protein | CP012516.1 | *Burkholderia pseudomallei* strain vgh16R chromosome 2, complete |
| Rieske (2Fe-2S) protein | CP012093.1 | *Burkholderia pseudomallei* strain 350105 chromosome 2 sequence. |
| Rieske (2Fe-2S) protein | CP009550.1 | *Burkholderia pseudomallei* PB08298010 chromosome II, complete |
| Rieske (2Fe-2S) protein | CP009297.1 | *Burkholderia pseudomallei* 406e chromosome 2, complete sequence. |
| Rieske (2Fe-2S) protein | CP004380.1 | *Burkholderia pseudomallei* 1026b chromosome 2, complete sequence. |
| Rieske (2Fe-2S) protein | CP009163.1 | *Burkholderia pseudomallei* K42 chromosome 2, complete sequence. |
| Rieske (2Fe-2S) protein | CP009127.1 | *Burkholderia pseudomallei* strain BSR chromosome 2, complete |
| Rieske (2Fe-2S) protein | CP008835.1 | *Burkholderia pseudomallei* strain BGR chromosome 2, complete |
| Rieske (2Fe-2S) protein | CP008782.1 | *Burkholderia pseudomallei* strain Mahidol-1106a chromosome 2, |
| Rieske (2Fe-2S) protein | CP008778.1 | *Burkholderia pseudomallei* 576 chromosome 2, complete sequence. |
| Rieske (2Fe-2S) protein | CP008759.1 | *Burkholderia pseudomallei* strain 1106a chromosome 2, complete |
| Rieske (2Fe-2S) protein | CP004043.1 | *Burkholderia pseudomallei* MSHR146 chromosome 2, complete sequence. |
| Rieske (2Fe-2S) protein | CP004024.1 | *Burkholderia pseudomallei* MSHR511 chromosome 2, complete sequence. |
| Rieske (2Fe-2S) protein | CP004002.1 | *Burkholderia pseudomallei* NCTC 13178 chromosome 2, complete |
| Rieske (2Fe-2S) protein | CP004004.1 | *Burkholderia pseudomallei* NAU20B-16 chromosome 2, complete |
| Rieske family iron-sulfur cluster-binding | CP003782.1 | *Burkholderia pseudomallei* BPC006 chromosome II, complete sequence. |
| Rieske (2Fe-2S) protein | CP002834.1 | *Burkholderia pseudomallei* 1026b chromosome 2, complete sequence. |
| Rieske (2Fe-2S) protein | CP000573.1 | *Burkholderia pseudomallei* 1106a chromosome II, complete sequence. |
| Rieske (2Fe-2S) protein | CP009586.1 | *Burkholderia pseudomallei* strain PHLS 112 chromosome II, complete |
| Rieske (2Fe-2S) protein | CP008893.1 | *Burkholderia pseudomallei* HBPUB10303a chromosome 2, complete |
| Rieske (2Fe-2S) protein | CP008763.1 | *Burkholderia pseudomallei* strain MSHR346 chromosome 2, complete |
| Rieske (2Fe-2S) protein | CP002600.1 | *Burkholderia gladioli* BSR3 chromosome 2, complete sequence. |
| putative FAD-dependent monooxygenase | FN563149.1a | *Rhodococcus equi* 103S chromosome. |
| cytochrome P450 monooxygenase | FN563149.1b | *Rhodococcus equi* 103S chromosome. |
| iron-sulfur binding oxidoreductase | KJ598877.1 | *Rhodococcus equi* strain U-S-A-18 clone S3A2 iron-sulfur binding oxidoreductase gene, complete cds. |
| iron-sulfur binding oxidoreductase | FN563149.1c | *Rhodococcus equi* 103S chromosome. |
| 3-ketosteroid-9-alpha-hydroxylase oxygenase subunit | CP003949.1a | *Rhodococcus opacus* PD630, complete genome. |
| 3-ketosteroid 9alpha-hydroxylase component KshA | AP011115.1a | *Rhodococcus opacus* B4 DNA, complete genome. |
| 3-ketosteroid-9-alpha-hydroxylase | CP011341.1a | *Rhodococcus aetherivorans* strain IcdP1, complete genome. |
| ketosteroid-9-alpha-hydroxylase, oxygenase | CP000431.1a | *Rhodococcus jostii* RHA1, complete genome. |
| 3-ketosteroid-9-alpha-hydroxylase | CP008947.1a | *Rhodococcus opacus* strain R7 sequence. |
| KshA-like protein | CP011269.1a | *Mycobacterium fortuitum* strain CT6, complete genome. |
| 3-ketosteroid-9-alpha-hydroxylase | CP014258.1a | *Mycobacterium fortuitum* subsp. fortuitum DSM 46621 = ATCC 6841 |
| 3-ketosteroid-9-alpha-hydroxylase oxygenase subunit | CP009914.1a | *Mycobacterium* sp. VKM Ac-1817D, complete genome. |
| hypothetical protein | FN563149.1 | *Rhodococcus equi* 103S chromosome. |
| 3-ketosteroid-9-alpha-hydroxylase | CP011341.1b | *Rhodococcus aetherivorans* strain IcdP1, complete genome. |
| 3-ketosteroid 9alpha-hydroxylase oxygenase | HQ425873.1 | *Rhodococcus rhodochrous* strain DSM 43269 hypothetical protein gene, partial cds; and 3-ketosteroid 9alpha-hydroxylase oxygenase(kshA1), putative ferredoxin (fd1), hypothetical protein, and 3-ketosteroid 9alpha-hydroxylase reductase (kshB) genes, complete cds. |
| 3-ketosteroid-9-alpha-hydroxylase oxygenase subunit | CP006996.1 | *Rhodococcus pyridinivorans* SB3094, complete genome. |
| 3-ketosteroid-9-alpha-hydroxylase | CP011295.1 | *Rhodococcus erythropolis* strain BG43, complete genome. |
| 3-ketosteroid 9alpha-hydroxylase component KshA | CP003761.1 | *Rhodococcus erythropolis* CCM2595, complete genome. |
| probable 3-ketosteroid 9alpha-hydroxylase component KshA | AP008957.1 | *Rhodococcus erythropolis* PR4 DNA, complete genome. |
| probable dioxygenase Rieske iron-sulfur component | CP000431.1b | *Rhodococcus jostii* RHA1, complete genome. |
| 3-ketosteroid 9alpha-hydroxylase component KshA2 | AP011115.1b | *Rhodococcus opacus* B4 DNA, complete genome. |
| 3-ketosteroid-9-alpha-hydroxylase | CP007255.1 | *Rhodococcus erythropolis* R138, complete genome. |
| 3-ketosteroid-9-alpha-hydroxylase oxygenase subunit | CP003949.1b | *Rhodococcus opacus* PD630, complete genome. |
| 3-ketosteroid-9-alpha-hydroxylase | CP008947.1b | *Rhodococcus opacus* strain R7 sequence. |
| putative iron-sulfur binding oxidoreductase | FN563149.1e | *Rhodococcus equi* 103S chromosome. |
| 3-ketosteroid 9alpha-hydroxylase oxygenase | HQ425877.1 | *Rhodococcus rhodochrous* strain DSM 43269 hypothetical proteins and 3-ketosteroid 9alpha-hydroxylase oxygenase (kshA5) genes, complete cds; and hypothetical protein gene, partial cds. |
| 3-ketosteroid-9-alpha-hydroxylase oxygenase subunit | LN868938.1 | *Nocardia farcinica* genome assembly NCTC11134, chromosome : 1. |
| putative terminal oxygenase | AP006618.1 | *Nocardia farcinica* IFM 10152 DNA, complete genome. |
| 3-ketosteroid 9alpha-hydroxylase component KshA | AP011115.1c | *Rhodococcus opacus* B4 DNA, complete genome. |
| 3-ketosteroid-9-alpha-hydroxylase | CP008947.1c | *Rhodococcus opacus* strain R7 sequence. |
| Ketosteroid-9-alpha-hydroxylase, oxygenase | FO082843.1 | *Nocardia cyriacigeorgica* GUH-2 chromosome complete genome. |
| probable dioxygenase Rieske iron-sulfur | CP000431.1c | *Rhodococcus jostii* RHA1, complete genome. |
| 3-ketosteroid-9-alpha-hydroxylase oxygenase | CP003949.1c | *Rhodococcus opacus* PD630, complete genome. |
| 3-ketosteroid-9-alpha-hydroxylase | CP011853.1 | *Gordonia* sp. QH-11, complete genome. |
| 2Fe-2S)-binding protein | CP014646.1 | *Thauera humireducens* strain SgZ-1, complete genome. |
| Rieske (2Fe-2S) domain protein | CP000511.1a | *Mycobacterium vanbaalenii* PYR-1, complete genome. |
| Rieske (2Fe-2S | CP002385.1a | *Mycobacterium gilvum* Spyr1, complete genome. |
| Rieske (2Fe-2S) | CP003053.1 | *Mycobacterium chubuense* NBB4, complete genome. |
| ring-hydroxylating dioxygenase, large terminal subunit | CP003078.1a | *Mycobacterium* sp. JS623, complete genome. |
| 2Fe-2S ferredoxin | CP014258.1b | *Mycobacterium fortuitum* subsp. fortuitum DSM 46621 = ATCC 6841 |
| Rieske (2Fe-2S) domain-containing protein | CP009914.1b | *Mycobacterium* sp. VKM Ac-1817D, complete genome. |
| 2Fe-2S ferredoxin | CP011022.1 | *Mycobacterium* sp. NRRL B-3805, complete genome. |
| 2Fe-2S ferredoxin | CP006936.2 | *Mycobacterium neoaurum* VKM Ac-1815D, complete genome. |
| 2Fe-2S ferredoxin | CP009496.1 | *Mycobacterium smegmatis* strain INHR2, complete genome. |
| 2Fe-2S ferredoxin | CP009495.1 | *Mycobacterium smegmatis* strain INHR1, complete genome. |
| 2Fe-2S ferredoxin | CP009494.1 | *Mycobacterium smegmatis* str. MC2 155, complete genome. |
| 2Fe-2S ferredoxin | CP001663.1 | *Mycobacterium smegmatis* str. MC2 155, complete genome. |
| 2Fe-2S ferredoxin | CP000480.1 | *Mycobacterium smegmatis* str. MC2 155, complete genome. |
| 2Fe-2S ferredoxin | LN831039.1 | *Mycobacterium smegmatis* genome assembly NCTC8159, chromosome : 1. |
| 2Fe-2S ferredoxin | CP010114.1a | *Mycobacterium avium* subsp. paratuberculosis strain E93, complete |
| 2Fe-2S ferredoxin | CP010113.1a | *Mycobacterium avium* subsp. paratuberculosis strain E1, complete |
| putative oxidoreductase, rieske (2Fe-2S | CP005928.1a | *Mycobacterium avium* subsp. paratuberculosis MAP4, complete genome. |
| hypothetical protein | AE016958.1a | *Mycobacterium avium* subsp. paratuberculosis str. k10, complete |
| 2Fe-2S ferredoxin | CP009614.1a | *Mycobacterium avium* subsp. avium strain DJO-44271, complete genome. |
| 2Fe-2S ferredoxin | CP009482.1a | *Mycobacterium avium* subsp. avium 2285 (S), complete genome. |
| 2Fe-2S ferredoxin | CP009493.1a | *Mycobacterium avium* subsp. avium 2285 (R), complete genome. |
| 2Fe-2S ferredoxin | CP000479.1a | *Mycobacterium avium* 104, complete genome. |
| hypothetical protein | CP011269.1b | *Mycobacterium fortuitum* strain CT6, complete genome. |
| 2Fe-2S ferredoxin | AP012555.1a | *Mycobacterium avium* subsp. hominissuis TH135 chromosomal DNA, |
| 2Fe-2S ferredoxin | CP002275.1 | *Mycobacterium indicus pranii* MTCC 9506, complete genome. |
| rieske (2Fe-2S) | CP003324.1 | *Mycobacterium intracellulare* MOTT-64, complete genome. |
| 3-ketosteroid-9-alpha-monooxygenase oxygenase subunit | CP014475.1 | *Mycobacterium phlei* strain CCUG 21000, complete genome. |
| rieske (2Fe-2S) | CP003347.1a | *Mycobacterium* sp. 05-1390, complete genome. |
| rieske (2Fe-2S) | CP003323.1 | *Mycobacterium intracellulare* MOTT-02, complete genome. |
| rieske (2Fe-2S) | CP003322.1 | *Mycobacterium intracellulare* ATCC 13950, complete genome. |
| rieske (2Fe-2S) | CP012150.1 | *Mycobacterium goodii* strain X7B, complete genome. |
| rieske (2Fe-2S) | CP003491.1a | *Mycobacterium* sp. MOTT36Y, complete genome. |
| oxidoreductase | CP002329.1 | *Mycobacterium sinense* strain JDM601, complete genome. |
| rieske (2Fe-2S) | CP009499.1a | *Mycobacterium intracellulare* 1956, complete genome. |
| Rieske (2Fe-2S) domain-containing protein | CP003053.1 | *Mycobacterium chubuense* NBB4, complete genome. |
| 3-ketosteroid-9-alpha-hydroxylase | CP014258.1c | *Mycobacterium fortuitum* subsp. fortuitum DSM 46621 = ATCC 6841 |
| Rieske (2Fe-2S) domain-containing protein | CP009914.1c | *Mycobacterium* sp. VKM Ac-1817D, complete genome. |
| KshA-like protein | CP011269.1c | *Mycobacterium fortuitum* strain CT6, complete genome. |
| 3-ketosteroid-9-alpha-hydroxylase | CP011773.1 | *Mycobacterium* sp. EPa45, complete genome. |
| oxygenase KshA | CP003347.1b | *Mycobacterium* sp. 05-1390, complete genome. |
| ring-hydroxylating dioxygenase, large terminal | CP002385.1b | *Mycobacterium gilvum* Spyr1, complete genome. |
| 3-ketosteroid-9-alpha-hydroxylase | CP009614.1b | *Mycobacterium avium* subsp. avium strain DJO-44271, complete genome. |
| 3-ketosteroid-9-alpha-hydroxylase | CP009482.1b | *Mycobacterium avium* subsp. avium 2285 (S), complete genome. |
| 3-ketosteroid-9-alpha-hydroxylase | CP009493.1b | *Mycobacterium avium* subsp. avium 2285 (R), complete genome. |
| oxygenase KshA | CP000479.1b | *Mycobacterium avium* 104, complete genome. |
| Rieske (2Fe-2S) domain protein | CP000656.1 | *Mycobacterium gilvum* PYR-GCK, complete genome. |
| oxygenase KshA | CP003491.1b | *Mycobacterium* sp. MOTT36Y, complete genome. |
| 3-ketosteroid-9-alpha-hydroxylase | CP010114.1b | *Mycobacterium avium* subsp. paratuberculosis strain E93, complete |
| 3-ketosteroid-9-alpha-hydroxylase | CP010113.1b | *Mycobacterium avium* subsp. paratuberculosis strain E1, complete |
| oxidoreductase | CP005928.1b | *Mycobacterium avium* subsp. paratuberculosis MAP4, complete genome. |
| hypothetical protein | AE016958.1b | *Mycobacterium avium* subsp. paratuberculosis str. k10, complete |
| 3-ketosteroid-9-alpha-hydroxylase | CP009499.1b | *Mycobacterium intracellulare* 1956, complete genome. |
| oxygenase KshA | AP012555.1b | *Mycobacterium avium* subsp. hominissuis TH135 chromosomal DNA, |
| Rieske (2Fe-2S) domain protein | CP000518.1 | *Mycobacterium* sp. KMS, complete genome. |
| Rieske (2Fe-2S) region | CP000384.1 | *Mycobacterium* sp. MCS, complete genome. |
| Rieske (2Fe-2S) domain protein | CP000580.1 | *Mycobacterium* sp. JLS, complete genome. |
| Rieske (2Fe-2S) domain-containing protein | CP003078.1b | *Mycobacterium* sp. JS623, complete genome. |
| Rieske (2Fe-2S) domain protein | CP000511.1b | *Mycobacterium vanbaalenii* PYR-1, complete genome. |
| Rieske (2Fe-2S) region | CP000656.1b | *Mycobacterium gilvum* PYR-GCK, complete genome. |
| Rieske (2Fe-2S) domain-containing protein | CP002385.1c | *Mycobacterium gilvum* Spyr1, complete genome. |
| hydroxylase of 3-hydroxy-9, 10-secoandrosta -1, 3, 5(10)-triene-9, 17-dione, flavin-dependent monooxygenase oxygenase subunit | LC010134.1b | *Comamonas testosteroni* DNA, putative mega-cluster of steroid degradation genes, strain: T441. |
| Acyl-CoA dehydrogenase, type 2-like protein | CP001220.2 | *Comamonas testosteroni* CNB-2, complete genome. |
| flavin-dependent monooxygenase oxygenase subunit | CP006704.1 | *Comamonas testosteroni* TK102, complete genome. |
| hydroxylase of 3-hydroxy-9, 10-secoandrosta -1, 3, 5(10)-triene-9, 17-dione | LC010134.1c | *Comamonas testosteroni* DNA, putative mega-cluster of steroid t441 |
| chromosome I | LN879547.1b | *Comamonas testosteroni* P19 genome assembly Comamonas testosteroni P19, chromosome : I |
| flavin reductase-like, FMN-binding protein | CP001220.2b | *Comamonas testosteroni* CNB-2, complete genome |
| flavin reductase | CP006704.1b | *Comamonas testosteroni* TK102, complete genome. |
| ORF11 | AB063482.1c | *Comamonas testosteroni* ORF11, tesA, tesD, tesE, tesF, tesG genes, complete cds. |
| 3-ketosteroid-9-alpha-hydroxylase reductase subunit | LC010134.1d | *Comamonas testosteroni* DNA, putative mega-cluster of steroid degradation genes, strain: T441. |
| chromosome I | LN879547.1c | *Comamonas testosteroni* P19 genome assembly Comamonas testosteroni P19, chromosome : I. |
| FAD-binding oxidoreductase | CP001220.2c | *Comamonas testosteroni* CNB-2, complete genome |
| 3-ketosteroid-9-alpha-hydroxylase reductase subunit | CP006704.1d | *Comamonas testosteroni* TK102, complete genome. |
| FAD-binding oxidoreductase | CP001220.2d | *Comamonas testosteroni* CNB-2, complete genome |
| chromosome i | LN879547.1d | *Comamonas testosteroni* P19 genome assembly *Comamonas testosteroni* P19, chromosome : I. |
| Acyl-CoA dehydrogenase, type 2-like protein | CP001220.2e | *Comamonas testosteroni* CNB-2, complete genome |
| flavin-dependent monooxygenase oxygenase subunit | CP006704.1e | *Comamonas testosteroni* TK102, complete genome. |
| 3-ketosteriod isomerase | AB489116 | *Comamonas testosteroni* genes for 3-ketosteroiod isomerase, 3-alpha steroid dehydrogenase, complete cds, strain: TA441. |
| ketosteroid isomerase | NZ_AKCL01000160 | *Pseudomonas putida* SJTE-1 contig000160, whole genome shotgun sequence. |
| ketosteroid isomerase | NZ_AKCL01000166 | *Pseudomonas putida* SJTE-1 contig000166, whole genome shotgun sequence. |
| steroid delta-isomerase | NZ_AFMP01000031 | *Sphingomonas* sp. KC8 contig31, whole genome shotgun sequence. |
| steroid delta-isomerase | NZ_AFMP01000031 | *Sphingomonas* sp. KC8 contig31, whole genome shotgun sequence. |
| putative 3-ketosteroid-5-isomerase | NZ_AFMP01000031 | *Sphingomonas* sp. KC8 contig31, whole genome shotgun sequence. |
| steroid delta-isomerase | NZ_AFMP01000018 | *Sphingomonas* sp. KC8 contig18, whole genome shotgun sequence. |
| steroid delta-isomerase | LC010134 | *Comamonas testosteroni* DNA, putative mega-cluster of steroid degradation genes, strain: T441. |
| conserved hypothetical protein | CP001220 | *Comamonas testosteroni* CNB-2, complete genome. |
| ketosteroid isomerase | M22749 | *Pseudomonas testosteroni* ketosteroid isomoerase (ksi) gene, complete cds. |
| delta-5-3-ketosteroid isomerase | J03568 | *P.testosteroni* delta-5-3-ketosteroid isomerase gene, complete cds. |
| steroid delta-isomerase | CP006704 | *Comamonas testosteroni* TK102, complete genome. |
| TesF | AB063482 | *Comamonas testosteroni* ORF11, tesA, tesD, tesE, tesF, tesG genes, complete cds. |
| TesF (acetaldehyde dehydrogenase) | LC010134 | *Comamonas testosteroni* DNA, putative mega-cluster of steroid degradation genes, strain: T441. |
| Acetaldehyde dehydrogenase | CP001220 | *Comamonas testosteroni* CNB-2, complete genome. |
| Acetaldehyde dehydrogenase | CP001220 | *Comamonas testosteroni* CNB-2, complete genome. |
| acetaldehyde dehydrogenase | CP006704 | *Comamonas testosteroni* TK102, complete genome. |
| TesG (possibly 4-hydroxy-2oxovalerate aldolase) | AB063482 | *Comamonas testosteroni* ORF11, tesA, tesD, tesE, tesF, tesG genes, complete cds. |
| 4-hyroxy-2-oxovalerate aldolase | CP006704 | *Comamonas testosteroni* TK102, complete genome. |
| 4-hyroxy-2-oxovalerate aldolase | CP006704 | *Comamonas testosteroni* TK102, complete genome. |
| 4-hyroxy-2-oxovalerate aldolase | LC010134 | *Comamonas testosteroni* DNA, putative mega-cluster of steroid degradation genes, strain: T441 |
| aphG 4-hydroxy-2-oxovalerate aldolase | AB029044 | *Comamonas testosteroni* orfX, orfY, aphT, aphC, aphE, aphF, aphG, aphH, orfJ, aphI gene cluster for meta-pathway enzymes required for degradation of phenol, complete cds |
| 4-hydroxy-2-oxovalerate aldolase (tdnJ) | NG_035478 | *Pseudomonas putida* UCC22 plasmid pTDN1 genes for conversion of aniline to catechol, aniline degradation lower-pathway gene cluster, regulatory protein and transposase, partial and complete cds |
| TadJ 4-hydroxy-2-oxovalerate aldolase | AY940090 | *Delftia tsuruhatensis* strain AD9 transposase (tnpA-L1) gene, partial cds; tad gene cluster, complete sequence; transcriptional regulator (orfX), hydrolase/acyltransferase (orfY), and muconate cycloisomerase (orfZ) genes, complete cds; and transposase (tnpA-L2) gene, partial cds |
| 4-hydroxy-2-oxovalerate aldolase | CP000539 | *Acidovorax* sp. JS42, complete genome. |
| 4-hydroxy-2-oxovalerate aldolase | AF190463 | *Comamonas* sp. JS765 hypothetical CdoX1 (cdoX1), hypothetical CmpX-like protein (cdoX2), hypothetical CdoFa (cdoFa), hypothetical CdoFb (cdoFb), hypothetical CdoR2 (cdoR2), hypothetical CdoX3(cdoX3), 2-hydroxymuconic semialdehyde dehydrogenase (cdoG), 2-oxo-4-pentenoate hydratase (cdoH), acetaldehyde dehydrogenase(cdoI), 4-hydroxy-2-oxovalerate aldolase (cdoJ), and4-oxalocrotonate decarboxylase (cdoK) genes, complete cds; and CdoL (cdoL) gene, partial cds. |
| 4-hydroxy-2-oxovalerate aldolase (pcaJ) | FJ601374 | *Diaphorobacter* sp. PCA039 pca gene cluster, complete sequence. |
| 4-hydroxy-2-oxovalerate aldolase | FR687359 | *Burkholderia rhizoxinica* HKI 454, complete genome. |
| TesE | AB063482 | *Comamonas testosteroni* ORF11, tesA, tesD, tesE, tesF, tesG genes, complete cds. |
| 2-keto-4-pentenoate hydratase (hydratase/decarboxylase) | CP001220 | *Comamonas testosteroni* CNB-2, complete genome. |
| 2-keto-4-pentenoate hydratase | CP006704 | *Comamonas testosteroni* TK102, complete genome. |
| 2-hydroxyhexa-2,4-dienoate hydratase | LC010134 | *Comamonas testosteroni* DNA, putative mega-cluster of steroid degradation genes, strain: T441. |
| TesD/ORF13 | AB063482 | *Comamonas testosteroni* ORF11, tesA, tesD, tesE, tesF, tesG genes, complete cds. |
| TesD (hydrolase for 4, 5-9, 10-Diseco-3-hydroxy-5, 9,  17-trioxoandrosta-1(10), 2-dien-4-oic acid) | LC010134 | *Comamonas testosteroni* DNA, putative mega-cluster of steroid degradation genes, strain: T441. |
| alpha/beta hydrolase fold protein | CP001220 | *Comamonas testosteroni* CNB-2, complete genome. |
| 3-oxoacyl-ACP reductase | CP006704 | *Comamonas testosteroni* TK102, complete genome. |
| Chromosome I | LN879547.1 | *Comamonas testosteroni* P19 genome assembly *Comamonas testosteroni* P19, chromosome : I |
| 2-hydroxymuconic semialdehyde hydrolase | JAFFSZ010000000 | *Rhodococcus equi* ATCC13557 (this paper) |
| putative alpha/beta hydrolase | FN563149.1 | *Rhodococcus equi* 103S chromosome |
| hypothetical protein | AB117721.1 | *Rhodococcus rhodochrous* orf31, orf32, bphC3 genes for hypothetical proteins and 2, 3-dihydroxybiphenyl 1, 2-dioxygenase, partial and complete cds. |
| Oxosteriod 1-dehydrogenase | LGTW01000000 | *Streptomyces scabiei* DNA, scaffold: NODE_015_cov_67.063, strain: S58, whole genome shotgun sequence |
| 3- Oxosteriod 1-dehydrogenase | BCMM01000015 | *Streptomyces scabiei* DNA, scaffold: NODE_015_cov_67.063, strain: S58, whole genome shotgun sequence |
| Oxosteriod 1-dehydrogenase | BCMM01000026 | *Streptomyces scabiei* DNA, scaffold: NODE_026_cov_60.1768, strain: S58, whole genome shotgun sequence |
| 3- Oxosteriod 1-dehydrogenase | LMXT01000039.1 | *Comamonas testosteroni* strain WDL7 contig_68, whole genome shotgun sequence |
| 3-oxosteroid 1-dehydrogenase | LLWJ01000045.1 | *Pseudomonas* sp. TAA207 contig000045, whole genome shotgun sequence |
| 3-oxosteroid 1-dehydrogenase | LLWI01000055.1 | *Pseudomonas* sp. TAD18 contig000055, whole genome shotgun sequence |
| 3-oxosteroid 1-dehydrogenase | BCMK01000015.1 | *Streptomyces acidiscabies* DNA, scaffold: NODE_015_cov_60.072, strain: a10, whole genome shotgun sequence |
| 3-oxosteroid 1-dehydrogenase | BCMN01000006.1 | *Streptomyces turgidiscabies* DNA, scaffold: NODE_006_cov_57.7002, strain: T45, whole genome shotgun sequence |
| 3-oxosteroid 1-dehydrogenase | BCMN01000025.1 | *Streptomyces turgidiscabies* DNA, scaffold: NODE_025_cov_64.0769, strain: T45, whole genome shotgun sequence |
| 3-oxosteroid 1-dehydrogenase | BCMN01000034.1 | *Streptomyces turgidiscabies* DNA, scaffold: NODE_034_cov_67.2766, strain: T45, whole genome shotgun sequence |
| 3-oxosteroid 1-dehydrogenase | CP012901.1 | *Pseudomonas aeruginosa* strain N15-01092, complete sequence |
| 3-oxosteroid 1-dehydrogenase | LOEI01000003.1 | *Klebsiella pneumoniae* strain OC511 AOT23_contig000003, whole genome shotgun sequence |
| 3-oxosteroid 1-dehydrogenase | LOEJ01000018.1 | *Klebsiella pneumoniae* strain K1 AOT20_contig000018, whole genome shotgun sequence |
| 3-oxosteroid 1-dehydrogenase | LOEF01000003.1 | *Klebsiella pneumoniae* strain OC217 AOT21_contig000003, whole genome shotgun sequence |
| 3-oxosteroid 1-dehydrogenase | LOEH01000004.1 | *Klebsiella pneumoniae* strain OC648 AOT24_contig000004, whole genome shotgun sequence |
| 3-oxosteroid 1-dehydrogenase | LOEG01000006.1 | *Klebsiella pneumoniae* strain Z3209 AOT25_contig000006, whole genome shotgun sequence |
| 3-oxosteroid 1-dehydrogenase | CP007255.1 | *Rhodococcus erythropolis* R138, complete genome |
| 3-ketosteroid-delta-1-dehydrogenase | AZXY01000001.1 | *Rhodococcus pyridinivorans* KG-16 contig00001, whole genome shotgun sequence |
| 3-ketosteroid-delta-1-dehydrogenase | LNQH01000008.1 | *Rhodococcus enclensis* strain NIO-1009 contig008, whole genome shotgun sequence |
| catechol 1,2-dioxygenase (1a) | NZ_AKCL01000002 | *Pseudomonas putida* SJTE-1 contig000002, whole genome shotgun sequence. |
| catechol 1,2-dioxygenase | NZ_AKCL01000193 | *Pseudomonas putida* SJTE-1 contig000193, whole genome shotgun sequence. |
| catechol 2,3-dioxygenase | NC_008275 | *Pseudomonas putida* MT53 plasmid pWW53, complete sequence |
| Catechol 2,3 dioxygenase | NC_003350 | *Pseudomonas putida* plasmid pWW0, complete sequence. |
| Catechol 2,3 dioxygenase | NC_007926 | *Pseudomonas putida* plasmid NAH7, complete sequence. |
| Catechol 2,3 dioxygenase | NC_004999 | *Pseudomonas putida* NCIB 9816-4 plasmid pDTG1, complete sequence. |
| Catechol 2,3 dioxygenase | NC_008275 | *Pseudomonas putida* MT53 plasmid pWW53, complete sequence. |
|  |  |  |
|  |  |  |
|  |  |  |
|  |  |  |
|  |  |  |
|  |  |  |
|  |  |  |
|  |  |  |
|  |  |  |
|  |  |  |
| Catechol 2,3 dioxygenase | NC_014124 | *Pseudomonas putida* plasmid pDK1, complete sequence. |
| Catechol 2,3 dioxygenase | NC_010678 | *Ralstonia pickettii* 12J chromosome 2, complete sequence. |
| Catechol 2,3 dioxygenase | NC_007494 | *Rhodobacter sphaeroides* 2.4.1 chromosome 2, complete sequence |
| Catechol 2,3 dioxygenase | NC_010175 | *Chloroflexus aurantiacus* J-10-fl chromosome, complete genome. |
| Catechol 2,3 dioxygenase | NC_012674 | *Pseudomonas fluorescens* strain PC20 plasmid pNAH20, complete sequence |
| Catechol 2,3 dioxygenase | NC_002033 | *Novosphingobium aromaticivorans* plasmid pNL1, complete sequence. |
| Catechol 2,3 dioxygenase (a) | NC_002754 | *Sulfolobus solfataricus* P2 chromosome, complete genome. |
| Catechol 2,3 dioxygenase | NC_008269 | *Rhodococcus jostii* RHA1 plasmid pRHL1, complete sequence. |
| Catechol 2,3 dioxygenase | NC_016644 | *Pseudomonas* sp. MC1 plasmid KOPRI126573, complete sequence. |
| Catechol 2,3 dioxygenase | NC_021250 | *Pseudomonas migulae* strain D2RT plasmid pD2RT, complete sequence. |
| Catechol 2,3 dioxygenase | NC_013446 | *Comamonas testosteroni* CNB-2, complete genome. |
| Catechol 2,3 dioxygenase | NC_018028 | *Pseudomonas stutzeri* CCUG 29243, complete genome. |
| Catechol 2,3 dioxygenase | NC_000964 | *Bacillus subtilis* subsp. subtilis str. 168 chromosome, complete genome. |
|  |  |  |
|  |  |  |
| Catechol 2,3 dioxygenase (a) | NC_008268 | *Rhodococcus jostii* RHA1, complete genome. |
| Catechol 2,3 dioxygenase | NC_012589 | *Sulfolobus islandicus* L.S.2.15 chromosome, complete genome. |
| Catechol 2,3 dioxygenase | NZ_KB900701 | *Bradyrhizobium elkanii* USDA 76 BraelDRAFT_scaffold1.1, whole genome shotgun sequence |
| Catechol 2,3 dioxygenase | NC_006462 | *Thermus thermophilus* HB8 plasmid pTT27, complete sequence. |
| Catechol 2,3 dioxygenase | NC_007973 | *Cupriavidus metallidurans* CH34, complete genome. |
| Catechol 2,3 dioxygenase | NC_012726 | *Sulfolobus islandicus* M.16.4 chromosome, complete genome. |
| Catechol 2,3 dioxygenase | NC_012623 | *Sulfolobus islandicus* Y.N.15.51 chromosome, complete genome. |
| Catechol 2,3 dioxygenase | NC_013769 | *Sulfolobus islandicus* L.D.8.5 chromosome, complete genome. |
| Catechol 2,3 dioxygenase | NC_012586 | *Sinorhizobium fredii* NGR234 plasmid pNGR234b, complete sequence. |
| Catechol 2,3 dioxygenase | NC_020995 | *Enterococcus casseliflavus* EC20, complete genome. |
| Catechol 2,3 dioxygenase (b) | NC_002754 | *Sulfolobus solfataricus* P2 chromosome, complete genome. |
| Catechol 2,3 dioxygenase | NC_017276 | *Sulfolobus islandicus* REY15A chromosome, complete genome. |
| Catechol 2,3 dioxygenase | NC_017275 | *Sulfolobus islandicus* HVE10/4 chromosome, complete genome. |
| Catechol 2,3 dioxygenase | NC_014318 | *Amycolatopsis mediterranei* U32 chromosome, complete genome. |
| Catechol 2,3 dioxygenase | NC_007181 | *Sulfolobus acidocaldarius* DSM 639 chromosome, complete genome. |
| Catechol 2,3 dioxygenase | NC_016978 | *Comamonas testosteroni* plasmid pI2, complete sequence. |
| Catechol 2,3 dioxygenase | NC_020247 | *Sulfolobus acidocaldarius* Ron12/I, complete genome. |
| Catechol 2,3 dioxygenase | NC_020246 | *Sulfolobus acidocaldarius* N8, complete genome. |
| Catechol 2,3 dioxygenase | NC_006270 | *Bacillus licheniformis* ATCC 14580, complete genome. |
| Catechol 2,3 dioxygenase | NC_003997 | *Bacillus anthracis* str. Ames chromosome, complete genome. |
| Catechol 2,3 dioxygenase | NC_014551 | *Bacillus amyloliquefaciens* DSM7 complete genome. |
| Catechol 1,2 dioxygenase | NC_007953 | *Burkholderia xenovorans* LB400 chromosome 3, complete sequence. |
| Catechol 2,3 dioxygenase | NC_016047 | *Bacillus subtilis* subsp. spizizenii TU-B-10, complete genome. |
| Catechol 2,3 dioxygenase | NC_014622 | *Paenibacillus polymyxa* SC2, complete genome. |
| Catechol 2,3 dioxygenase | NC_005945 | *Bacillus anthracis* str. Sterne chromosome, complete genome. |
| Catechol 2,3 dioxygenase | NZ_CP007640 | *Bacillus atrophaeus* subsp. globigii strain BSS. |
| Catechol 2,3 dioxygenase (short removed) | NC_005042 | *Prochlorococcus marinus* subsp. marinus str. CCMP1375 complete genome |
| Catechol 1,2 dioxygenase | NC_006351 | *Burkholderia pseudomallei* K96243 chromosome 2, complete sequence. |
| Catechol 1,2 dioxygenase (a) | NC_002947 | *Pseudomonas putida* KT2440 chromosome, complete genome. |
| Catechol 1,2 dioxygenase | NC_011002 | *Burkholderia cenocepacia* J2315 chromosome 3, complete genome. |
| Catechol 1,2 dioxygenase (b) | NC_002947 | *Pseudomonas putida* KT2440 chromosome, complete genome. |
| Catechol 1,2 dioxygenase (a) | NZ_HG938353 | *Neorhizobium galegae*, complete genome. |
| Catechol 1,2 dioxygenase (a) | NC_010682 | *Ralstonia pickettii* 12J chromosome 1, complete sequence. |
| Catechol 1,2 dioxygenase | NZ_KI530699 | *Acinetobacter gyllenbergii* NIPH 230 adfcq-supercont1.1, whole genome shotgun sequence |
| catechol 1,2-dioxygenase | NC_003078 | *Sinorhizobium meliloti* 1021 plasmid pSymB, complete sequence. |
| Catechol 1,2 dioxygenase (a) | NC_003450 | *Corynebacterium glutamicum* ATCC 13032 chromosome, complete genome. |
|  |  |  |
|  |  |  |
| catechol 1,2-dioxygenase | NC_004463 | *Bradyrhizobium japonicum* USDA 110 chromosome, complete genome. |
|  |  |  |
|  |  |  |
| catechol 1,2-dioxygenase | NZ_KB849655 | *Acinetobacter junii* CIP 64.5 acLZZ-supercont1.3, whole genome |
|  |  |  |
| Catechol 1,2 dioxygenase | NC_015663 | *Enterobacter aerogenes* KCTC 2190 chromosome, complete genome. |
| Catechol 1,2 dioxygenase | NZ_KB851227 | *Acinetobacter lwoffii* NCTC 5866 = CIP 64.10 acLsp-supercont1.12, whole shotgun sequence. |
| catechol 1,2 dioxygenase | NZ_KB849749 | *Acinetobacter radioresistens* DSM 6976 = NBRC 102413 = CIP 103788  acLrZ-supercont1.9, whole genome shotgun sequence. |
|  |  |  |
| Catechol 1,2 dioxygenase (a) | NZ_KI421499 | *Bradyrhizobium genosp*. SA-4 str. CB756 BrageDRAFT_scaffold1.1,  whole genome shotgun sequence. |
|  |  |  |
| Catechol 1,2 dioxygenase | NC_025133 | *Sphingobium wenxiniae* strain JZ-1 plasmid pPBA, complete sequence. |
| Catechol 2,3 dioxygenase (b) | NZ_KI421499 | *Bradyrhizobium genosp*. SA-4 str. CB756 BrageDRAFT_scaffold1.1, whole genome shotgun sequence. |
| Catechol 1,2 dioxygenase | NC_002516 | *Pseudomonas aeruginosa* PAO1 chromosome, complete genome. |
|  |  |  |
|  |  |  |
|  |  |  |
|  |  |  |
|  |  |  |
|  |  |  |
|  |  |  |
|  |  |  |
|  |  |  |
| extradiol catechol dioxygenase (a) | NZ_AKCL01000023 | *Pseudomonas putida* SJTE-1 contig000023, whole genome shotgun sequence. |
| putative aromatic ring-opening dioxygenase | FN563149.1 | *Rhodococcus equi* 103S chromosome. |
| 2, 3-dihydroxybiphenyl 1, 2-dioxygenase | AB117721.1 | *Rhodococcus rhodochrous* orf31, orf32, bphC3 genes for hypothetical proteins and 2, 3-dihydroxybiphenyl 1, 2-dioxygenase, partial and complete cds. |
| protocatechuate 4,5-dioxygenase | CP003588.1 | *Pseudomonas putida* ND6, complete genome. |
| protocatechuate 4,5-dioxygenase beta subunit / protocatechuate 4,5-dioxygenase alpha subunit | CP000712.1 | *Pseudomonas putida* F1, complete genome. |
| protocatechuate 4,5-dioxygenase | CP003734.1 | *Pseudomonas putida* DOT-T1E, complete genome. |
| gallate dioxygenase | AE015451.2 | *Pseudomonas putida* KT2440 complete genome |
| Protocatechuate 4,5-dioxygenase | CP002290.1 | *Pseudomonas putida* BIRD-1, complete genome. |
| protocatechuate 4,5-dioxygenase subunit alpha | CP005976.1 | *Pseudomonas putida* H8234, complete genome. |
| Protocatechuate 4,5-dioxygenase | CP000949.1 | *Pseudomonas putida* W619, complete genome. |
| hydroxyquinol 1,2-dioxygenase | CP005959.1 | *Corynebacterium glutamicum* MB001, complete genome. |
| putative hydroxyquinol/catechol 1,2-dioxygenase | HE802067.1 | *Corynebacterium glutamicum* K051 complete genome, strain ATCC 13032, sub-strain K051. |
| Protocatechuate 3,4-dioxygenase beta subunit | BA000036.3 | *Corynebacterium glutamicum* ATCC 13032 DNA, complete genome. |
| CATECHOL 1,2-DIOXYGENASE | BX927157.1 | *Corynebacterium glutamicum* ATCC 13032, IS fingerprint type 4-5, complete genome; segment 10/10. |
| hydroxyquinol 1,2-dioxygenase | CP012194.1 | *Corynebacterium glutamicum* strain CP, complete genome. |
| hydroxyquinol 1,2-dioxygenase | CP011309.1 | [*Brevibacterium] flavum* strain ATCC 15168, complete genome. |
| hydroxyquinol 1,2-dioxygenase | CP010451.1 | *Corynebacterium glutamicum* strain B253, complete genome. |
| hypothetical protein | AP009044.1 | *Corynebacterium glutamicum* R DNA, complete genome. |
| hydroxyquinol 1,2-dioxygenase | CP013991.1 | *Corynebacterium glutamicum* strain USDA-ARS-USMARC-56828, complete |
| COG3485 Protocatechuate 3,4-dioxygenase beta subunit | CP004048.1 | *Corynebacterium glutamicum* SCgG2, complete genome. |
| Protocatechuate 3,4-dioxygenase beta subunit | CP004047.1 | *Corynebacterium glutamicum* SCgG1, complete genome. |
| hydroxyquinol 1,2-dioxygenase | CP007724.1 | *Corynebacterium glutamicum* strain AR1, complete genome. |
| hydroxyquinol 1,2-dioxygenase | CP007722.1 | *Corynebacterium glutamicum* strain ATCC 21831, complete genome. |
| catechol 1,2-dioxygenase | CP007569.1 | *Bradyrhizobium japonicum* SEMIA 5079 genome. |
| catechol 1,2-dioxygenase | AP014685.1 | *Bradyrhizobium diazoefficiens* DNA, complete genome, strain: NK6. |
| catechol 1,2-dioxygenase | BA000040.2a | *Bradyrhizobium japonicum* USDA 110 DNA, complete genome. |
| catechol 1,2-dioxygenase | AP012206.1a | *Bradyrhizobium japonicum* USDA 6 DNA, complete genome. |
| catechol 1,2-dioxygenase | CP010313.1a | *Bradyrhizobium japonicum* strain E109, complete genome. |
| catechol 1,2-dioxygenase | CP013949.1 | *Bradyrhizobium* sp. CCGE-LA001, complete genome. |
| catechol 1,2-dioxygenase | CU234118.1 | *Bradyrhizobium* sp. ORS278,complete sequence. |
| catechol 1,2-dioxygenase | BA000040.2b | *Bradyrhizobium japonicum* USDA 110 DNA, complete genome. |
| catechol 1,2-dioxygenase | AP012206.1b | *Bradyrhizobium japonicum* USDA 6 DNA, complete genome. |
| catechol 1,2-dioxygenase | CP010313.1b | *Bradyrhizobium japonicum* strain E109, complete genome. |
| catechol 1,2-dioxygenase | CP000494.1 | *Bradyrhizobium* sp. BTAi1, complete genome. |
| catechol 1,2-dioxygenase | AP012603.1 | *Bradyrhizobium oligotrophicum* S58 DNA, complete genome. |
| catechol 1,2-dioxygenase | AP014704.1 | *Methylobacterium aquaticum* DNA, complete genome, strain: MA-22A. |
| TesB (meta-cleavage enzyme, 2,3-Dihydroxybiphenyl-1,2-dioxygenase) | AB040808 | *Comamonas testosteroni* tesB, ORF1, ORF2, ORF3 genes, complete cds. |
| homogentisate 1,2-dioxygenase (1) | NZ_AKCL01000092 | *Pseudomonas putida* SJTE-1 contig000092, whole genome shotgun sequence. |
| protocatechuate 3,4-dioxygenase alpha chain (2) | NZ_AKCL01000092 | *Pseudomonas putida* SJTE-1 contig000092, whole genome shotgun sequence. |
| protocatechuate 3,4-dioxygenase subunit beta (3) | NZ_AKCL01000092 | *Pseudomonas putida* SJTE-1 contig000092, whole genome shotgun sequence. |
| benzoate 1,2-dioxygenase subunit beta | NZ_AKCL01000002 | *Pseudomonas putida* SJTE-1 contig000002, whole genome shotgun sequence. |
| benzoate 1,2-dioxygenase subunit alpha | NZ_AKCL01000002 | *Pseudomonas putida* SJTE-1 contig000002, whole genome shotgun sequence. |
| taurine dioxygenase | NZ_AKCL01000007 | *Pseudomonas putida* SJTE-1 contig000007, whole genome shotgun sequence. |
| 4-hydroxyphenylpyruvate dioxygenase | NZ_AKCL01000201 | *Pseudomonas putida* SJTE-1 contig000201, whole genome shotgun sequence. |
| quercetin 2,3-dioxygenase | NZ_AKCL01000201 | *Pseudomonas putida* SJTE-1 contig000201, whole genome shotgun sequence. |
| alpha-ketoglutarate-dependent dioxygenase | NZ_AKCL01000201 | *Pseudomonas putida* SJTE-1 contig000201, whole genome shotgun sequence. |
| protocatechuate 4,5-dioxygenase subunit alpha | NZ_AKCL01000023 | *Pseudomonas putida* SJTE-1 contig000023, whole genome shotgun sequence. |
| Extradiol ring-cleavage dioxygenase III subunit B. | NZ_AKCL01000055 | *Pseudomonas putida* SJTE-1 contig000055, whole genome shotgun sequence. |
| 4-hydroxyphenylpyruvate dioxygenase | NZ_AKCL01000022 | *Pseudomonas putida* SJTE-1 contig000022, whole genome shotgun sequence. |
| 2-nitropropane dioxygenase | NZ_AKCL01000190 | *Pseudomonas putida* SJTE-1 contig000190, whole genome shotgun sequence. |
| 2-nitropropane dioxygenase | NZ_AKCL01000135 | *Pseudomonas putida* SJTE-1 contig000135, whole genome shotgun sequence. |
| taurine dioxygenase | NZ_AKCL01000081 | *Pseudomonas putida* SJTE-1 contig000081, whole genome shotgun sequence. |
| DOPA 4,5-dioxygenase | NZ_AKCL01000071 | *Pseudomonas putida* SJTE-1 contig000071, whole genome shotgun sequence. |
| taurine dioxygenase | NZ_AKCL01000071 | *Pseudomonas putida* SJTE-1 contig000071, whole genome shotgun sequence. |
| ring-cleaving dioxygenase | NZ_AKCL01000205 | *Pseudomonas putida* SJTE-1 contig000205, whole genome shotgun sequence. |
| phytanoyl-CoA dioxygenase | NZ_AFMP01000051 | *Sphingomonas* sp. KC8 contig50, whole genome shotgun sequence. |
| dioxygenase | NZ_AFMP01000051 | *Sphingomonas* sp. KC8 contig50, whole genome shotgun sequence. |
| dioxygenase | NZ_AFMP01000044 | *Sphingomonas* sp. KC8 contig43, whole genome shotgun sequence. |
| dioxygenase | NZ_AFMP01000039 | *Sphingomonas* sp. KC8 contig38, whole genome shotgun sequence. |
| 4-hydroxyphenylpyruvate dioxygenase | NZ_AFMP01000011 | *Sphingomonas* sp. KC8 contig11, whole genome shotgun sequence. |
| homogentisate 1,2-dioxygenase | NZ_AFMP01000011 | *Sphingomonas* sp. KC8 contig11, whole genome shotgun sequence. |
| 2-nitropropane dioxygenase | NZ_AFMP01000011 | *Sphingomonas* sp. KC8 contig11, whole genome shotgun sequence. |
| phytanoyl-CoA dioxygenase | NZ_AFMP01000016 | *Sphingomonas* sp. KC8 contig16, whole genome shotgun sequence. |
| phytanoyl-CoA dioxygenase | NZ_AFMP01000016 | *Sphingomonas* sp. KC8 contig16, whole genome shotgun sequence. |
| taurine dioxygenase | NZ_AFMP01000016 | *Sphingomonas* sp. KC8 contig16, whole genome shotgun sequence. |
| 2-nitropropane dioxygenase | NZ_AFMP01000016 | *Sphingomonas* sp. KC8 contig16, whole genome shotgun sequence. |
| 2-nitropropane dioxygenase | NZ_AFMP01000031 | *Sphingomonas* sp. KC8 contig31, whole genome shotgun sequence. |
| 2-nitropropane dioxygenase | NZ_AFMP01000031 | *Sphingomonas* sp. KC8 contig31, whole genome shotgun sequence. |
| glyoxalase/bleomycin resistance/protein/dioxygenase | NZ_AFMP01000031 | *Sphingomonas* sp. KC8 contig31, whole genome shotgun sequence. |
| biphenyl 2,3-dioxygenase | NZ_AFMP01000036 | *Sphingomonas* sp. KC8 contig35, whole genome shotgun sequence. |
| Rieske-type ring hydroxylating dioxygenase beta subunit | NZ_AFMP01000026 | *Sphingomonas* sp. KC8 contig26, whole genome shotgun sequence. |
| aromatic ring-cleaving dioxygenase | NZ_AFMP01000004 | *Sphingomonas* sp. KC8 contig04, whole genome shotgun sequence. |
| 2-nitropropane dioxygenase | NZ_AFMP01000059 | *Sphingomonas* sp. KC8 contig58, whole genome shotgun sequence. |
| glyoxalase/bleomycin resistance/protein/dioxygenase | NZ_AFMP01000009 | *Sphingomonas* sp. KC8 contig09, whole genome shotgun sequence. |
| tryptophan 2 3-dioxygenase | NZ_AFMP01000035 | *Sphingomonas* sp. KC8 contig70, whole genome shotgun sequence. |
| alpha-ketoglutarate-dependent dioxygenase | NZ_AFMP01000032 | *Sphingomonas* sp. KC8 contig32, whole genome shotgun sequence. |
| dioxygenase beta subunit | NZ_AFMP01000019 | *Sphingomonas* sp. KC8 contig19, whole genome shotgun sequence. |
| glyoxalase/bleomycin resistance/protein/dioxygenase | NZ_AFMP01000040 | *Sphingomonas* sp. KC8 contig39, whole genome shotgun sequence. |
| 3-hydroxyanthranilate 3,4-dioxygenase | NZ_AFMP01000040 | *Sphingomonas* sp. KC8 contig39, whole genome shotgun sequence. |
| glyoxalase/bleomycin resistance/protein/dioxygenase | NZ_AFMP01000013 | *Sphingomonas* sp. KC8 contig13, whole genome shotgun sequence. |
| protocatechuate 3,4-dioxygenase | NC_008314 | *Ralstonia* *eutropha* H16 chromosome 2. |
| protocatechuate 3,4-dioxygenase | NC_008313 | *Ralstonia* *eutropha* H16 chromosome 1. |
| hydroxyquinol 1,2-dioxygenase | NZ_KI421499 | *Bradyrhizobium* *genosp*. SA-4 str. CB756 BrageDRAFT_scaffold1.1, |
| protocatechuate dioxygenase | NC_008268 | *Rhodococcus* *jostii* RHA1, complete genome. |
| TesB | LC010134 | *Comamonas* *testosteroni* DNA, putative mega-cluster of steroid degradation genes, strain: T441 |
| biphenyl-2,3-diol-1,2-dioxygenase | CP001220 | *Comamonas* *testosteroni* CNB-2, complete genome. |
| biphenyl-2,3-diol-1,2-dioxygenase | AF493052 | *Comamonas* *testosteroni* biphenyl-2,3-diol-1,2-dioxygenase gene, complete cds. |
| biphenyl 2,3-dioxygenase | CP006704 | *Comamonas* *testosteroni* TK102, complete genome. |
| Dioxygenase | JF502261 | *Rhodococcus* sp. R04 dioxygenase gene, complete cds. |
| protocatechuate 3,4-dioxygenase | NC_013446 | *Comamonas* *testosteroni* CNB-2, complete genome. |
| protocatechuate 3,4-dioxygenase subunit beta | NC_013446 | *Comamonas* *testosteroni* CNB-2, complete genome. |
| protocatechuate 4,5-dioxygenase subunit alpha | NC_010002 | *Delftia* *acidovorans* SPH-1, complete genome. |
| protocatechuate 3,4-dioxygenase subunit beta | NC_010002 | *Delftia* *acidovorans* SPH-1, complete genome. |
| protocatechuate 3,4-dioxygenase subunit beta | NC_003450 | *Corynebacterium* *glutamicum* ATCC 13032 chromosome, complete genome. |
| protocatechuate 3,4-dioxygenase subunit beta | NC_010002 | *Delftia* *acidovorans* SPH-1, complete genome. |
| protocatechuate 3,4-dioxygenase subunit beta | NC_012586 | *Sinorhizobium* *fredii* NGR234 plasmid pNGR234b, complete sequence. |
| protocatechuate 3,4-dioxygenase | NC_008314 | *Ralstonia* *eutropha* H16 chromosome 2. |
| protocatechuate 3,4-dioxygenase subunit alpha | NC_020800 | *Xanthomonas* *axonopodis* Xac29-1, complete genome. |
| protocatechuate 3,4-dioxygenase subunit beta | NC_020800 | *Xanthomonas* *axonopodis* Xac29-1, complete genome. |
| protocatechuate 3,4-dioxygenase subunit beta | NC_006834 | *Xanthomonas* *oryzae* pv. oryzae KACC 10331, complete genome. |
| protocatechuate 3,4-dioxygenase | NZ_HG938353 | *Neorhizobium* *galegae*, complete genome. |
| protocatechuate 3,4-dioxygenase | NC_013446 | *Comamonas* *testosteroni* CNB-2, complete genome. |
| protocatechuate 4,5-dioxygenase subunit alpha | NC_010002 | *Delftia* *acidovorans* SPH-1, complete genome. |
| protocatechuate 3,4-dioxygenase subunit beta | NC_010002 | *Delftia* *acidovorans* SPH-1, complete genome. |
| protocatechuate 3,4-dioxygenase subunit beta | NC_003450 | *Corynebacterium* *glutamicum* ATCC 13032 chromosome, complete genome. |
| protocatechuate 3,4-dioxygenase subunit beta | NC_010002 | *Delftia* *acidovorans* SPH-1, complete genome. |
| protocatechuate 3,4-dioxygenase subunit beta | NC_012586 | *Sinorhizobium* *fredii* NGR234 plasmid pNGR234b, complete sequence. |
| protocatechuate 3,4-dioxygenase | NC_008314 | *Ralstonia* *eutropha* H16 chromosome 2. |
| protocatechuate 3,4-dioxygenase subunit alpha | NC_020800 | *Xanthomonas* *axonopodis* Xac29-1, complete genome. |
| protocatechuate 3,4-dioxygenase subunit beta | NC_020800 | *Xanthomonas* *axonopodis* Xac29-1, complete genome. |
| protocatechuate 3,4-dioxygenase subunit beta | NC_006834 | *Xanthomonas* *oryzae* pv. oryzae KACC 10331, complete genome. |
| protocatechuate 3,4-dioxygenase | NZ_HG938353 | *Neorhizobium* *galegae*, complete genome. |
| reductase | U49504 | *Pseudomonas* sp. |
| Dioxygenase | AF252550 | *Comamonas* *testosteroni* |
| dox operon (doxABCDEFGHIJ) genes | M60405 | *Pseudomonas* sp. |
| naphthalene dioxygenase | U49496 | *Pseudomonas* sp |
| naphthalene dioxygenase | AF004284 | *Pseudomonas* *putida* |
| naphthalene dioxygenase (nahAc) gene | AY048759 | *Pseudomonas* *fluorescens* |
| Dioxygenase | AB004059 | *Pseudomonas* *putida* |
| naphthalene dioxygenase | M83949 | *Pseudomonas* *putida* |
| naphthalene dioxygenase | AF039533 | *Pseudomonas* *stutzeri* |
| naphthalene dioxygenase | M23914 | *Pseudomonas* *putida* |
| naphthalene dioxygenase | AB255564 | uncultured bacterium |
| naphthalene dioxygenase | AF004283 | *Pseudomonas* *fluorescens* |
| naphthalene dioxygenase | D84146 | *Pseudomonas* *aeruginosa* |
| nitrobenzene dioxygenase | AF379638 | *Comamonas* sp. JS765 |
| naphthalene dioxygenase | DQ846881 | *Rhodococcus* *opacus* |
| aromatic dioxygenase | AF121905 | *Rhodococcus* sp. I24 |
| naphthalene dioxygenase | AY392424 | *Rhodococcus* sp. P200 |
| naphthalene dioxygenase | AY392423 | *Rhodococcus* sp. P400 |
| o-xylene oxygenase gene | AB206671 | *Rhodococcus* *opacus* TKN14 |
| NidA (nidA) gene | AY330100 | *Mycobacterium* sp. KMS |
| dioxygenase large alpha subunit (nidA) gene | DQ537942 | *Mycobacterium* *gilvum* strain czh-101 |
| dioxygenase large alpha subunit (nidA) gene | AF548343 | *Mycobacterium* *flavescens* strain PYR-GCK |
| Iron-sulfur protein large subunit | AB017794 | *Nocardioides* sp. |
| dioxygenase | EF026099 | *Mycobacterium* sp. SNP11 |
| dioxygenase | DQ358754 | *Mycobacterium* sp. CH-1 |
| extradiol dioxygenase | DQ157862 | *Mycobacterium* sp. CH-2 |
| biphenyl dioxygenase | AB113649 | *Bacillus* sp. JF8 |
| Ring hydroxylating dioxygenase | AJ494743 | *Mycobacterium* sp. 6PY1 |
| extradiol dioxygenase | AB031319 | *Nocardioides* sp. KP7 |
| NidB | DQ157863 | *Mycobacterium* sp. CH-2 |
| NidA (nidA) gene | AF548345 | *Mycobacterium* *frederiksbergense* strain FAn9T |
| phthalate dioxygenase | AY365117 | *Mycobacterium* *vanbaalenii* strain PYR-1 |
| naphthalene inducible dioxygenase | AF249301 | *Mycobacterium* sp. PYR-1 |
| NidA (nidA) gene | DQ537941 | *Mycobacterium* *pallens* strain czh-8 |
| dioxygenase | DQ358753 | *Mycobacterium* sp. CH-1 |
| NidA and NidB | AY330098 | *Mycobacterium* sp. JLS |
| NidA | AB179737 | *Mycobacterium* sp. MHP-1 |
| nidA | AF548347 | *Mycobacterium* *gilvum* strain BB1 |
| pdoA1 | AJ494745 | *Mycobacterium* sp. 6PY1 |
| NidA | AY330102 | *Mycobacterium* sp. MCS |
| dioxygenase | AF546905 | *Mycobacterium* sp. S65 |
| dioxygenase | AJ536756 | *Ralstonia* *oxalatica* transposon Tn4371. |
| biphenyl dioxygenase | AB086835 | *Comamonas* *testosteroni* |
| biphenyl dioxygenase | U47637 | *Pandoraea* *pnomenusa* strain B-356 |
| biphenyl dioxygenase | M83673 | *Pseudomonas* *pseudoalcaligenes* KF707 |
| biphenyl dioxygenase | AY027651 | *Pseudomonas* sp. Cam-1 |
| biphenyl dioxygenase | M86348 | *Paraburkholderia* *xenovorans* LB400 |
| biphenyl dioxygenase | U95054 | *Pseudomonas* sp. B4 |
| biphenyl dioxygenase | AF049345 | *Pseudomonas* *pseudoalcaligenes* |
| biphenyl dioxygenase | AJ010057 | *Burkholderia* sp. JB1 |
| terpA | EF527236 | *Rhodococcus* sp. L4 |
| dioxygenase | Y18245 | *Pseudomonas* *putida* |
| benzene dioxygenase | M17904 | *Pseudomonas* *putida* |
| biphenyl dioxygenase | AJ251217 | *Pseudomonas* sp. B4 |
| dioxygenase | AY831463 | *Pseudomonas* *putida* strain GJ31 |
| toluene dioxygenase | J04996 | *Pseudomonas* *putida* |
| dioxygenase | AJ006307 | *Ralstonia* sp. JS705 |
| dioxygenase | U15298 | *Pseudomonas* sp. |
| chlorobenzene dioxygenase | U78099 | *Burkholderia* sp. PS12 |
| cumene dioxygenase | D37828 | *Pseudomonas* *fluorescens* |
| ethylbenzene dioxygenase | AF049851 | *Pseudomonas* *fluorescens* |
| dioxygenase | U53507 | *Pseudomonas* JR1 |
| dioxygenase | AJ293587 | *Pseudomonas* *putida* |
| dioxygenase | AF006691 | *Pseudomonas* *putida* |
| dioxygenase | AB048707 | *Rhodococcus* sp. RHA1 |
| dioxygenase | AB120955 | *Rhodococcus* sp. RHA1 |
| dioxygenase | AB120956 | *Rhodococcus* sp. RHA1 |
| dioxygenase | AB048708 | *Rhodococcus* sp. RHA1 |
| terminal dioxygenase | U27591 | *Rhodococcus* sp. M5 |
| dioxygenase | X80041 | *Rhodococcus* *globerulus* |
| dioxygenase | D32142 | *Rhodococcus* sp. RHA1 |
| isopropylbenzene 2,3-dioxygenase | U24277 | *Rhodococcus* *erythropolis* |
| terminal dioxygenase | D88020 | *Rhodococcus* *erythropolis* |
| biphenyl dioxygenase | EF152282 | *Sphingobium* *yanoikuyae* |
| oxidoreductase | AF380367 | *Burkholderia* sp. DBT1 |
| dioxygenase | AB024945 | *Alcaligenes* *faecalis* |
| dioxygenase | DQ501245 | *Cycloclasticus* sp. P1 |
| Ring hydroxylating dioxygenase | AJ633551 | *Sphingomonas* sp. CHY-1 |
| extradiol dioxygenase | AF169302 | *Burkholderia* *cepacia* |
| dioxygenase | CP000511 | *Mycobacterium* *vanbaalenii* PYR-1 |
| dioxygenase | CP000479 | *Mycobacterium* *avium* 104 |
| dioxygenase | CP000509 | *Nocardioides* sp. JS614, |
| dioxygenase | CP000656 | *Mycobacterium* *gilvum* PYR-GCK |
| dioxygenase | AB091693 | *Sphingomonas* sp. P2 |
| dioxygenase | AAXZ01000003 | *Rhodobacteraceae* *bacterium* HTCC2150 |
| dioxygenase | AB272984 | *Rhodococcus* *rhodochrous* |
| dioxygenase | CH482384 | *Pseudomonas* *aeruginosa* 2192 |
| dioxygenase | EU024110 | *Sphingomonas* sp. LB126 fluorene/dibenzofuran degradation operon, |
| dioxygenase | AF060489 | *Sphingomonas* sp. CB3 |
| dioxygenase | AAQG01000001 | *Sphingomonas* sp. SKA58 |
| dioxygenase | AB201843 | *Paenibacillus* sp. YK5 |
| dioxygenase | U51165 | *Cycloclasticus* *oligotrophus* XYLX |
| dioxygenase | AB161232 | *Sphingomonas* sp. A4 |
| dioxygenase | AB240454 | *Sphingomonas* sp. A4 |
| dioxygenase | AB121977 | *Xanthobacter* *polyaromaticivorans* |
| dioxygenase | AF157565 | *Sphingopyxis* *macrogoltabida* strain TFA |
| dioxygenase | AB070456 | *Rhodococcus* sp. YK2 |
| dioxygenase | AF061751 | *Burkholderia* sp. strain RP007 |
| dioxygenase | CP000089 | *Dechloromonas* *aromatica* RCB |
| dioxygenase | D89064 | *Pseudomonas* sp. |
| dioxygenase | U18133 | *Conidiobolus* *coronatus* |
| pobA and pobB genes | X78823 | *P*.*pseudoalcaligenes* (POB310) |
| phthalate dioxygenase | AF095748 | *Burkholderia* *cepacia* |
| 2-halobenzoate 1,2-dioxygenase | X79076 | *Burkholderia* *cepacia* |
| dioxygenase | AF009224 | *Acinetobacter* sp. ADP1 |
| dioxygenase | AF071556 | *Acinetobacter* sp. ADP1 |
| dioxygenase | AB084235 | *Terrabacter* sp. DBF63 |
| dioxygenase | X72850 | *Sphingomonas* sp. |
| dioxygenase | D17319 | *Pseudomonas* sp. KKS102 |
| napthalene dioxygenase | AF082663 | *Rhodococcus* sp. NCIMB12038 |
| putative cis-naphthalene 1,2-dioxygenase | AJ401612 | *Rhodococcus* sp. 1BN |
| benzene dioxygenase | AF148496 | *Pseudomonas* *putida* |
| dioxygenase | EF600714 | *Pandoraea* *pnomenusa* |
| dioxygenase | AP011117 | *Rhodococcus* *opacus* B4 |
| dioxygenase | AF452376 | *Rhodococcus* *aetherivorans* strain I24 |
| dioxygenase | BA000007 | *Escherichia* *coli* O157:H7 str |
| dioxygenase | AE005174 | *Escherichia* *coli* O157:H7 str. EDL933 |
| dioxygenase | Z37966 | *Escherichia* *coli* K-12 |
| dioxygenase | U00096 | *Escherichia* *coli* str. K-12 substr. MG1655 |
| dioxygenase | AP009048 | *Escherichia* *coli* str. K-12 substr. W3110 |
| dioxygenase | CP000038 | *Shigella* *sonnei* Ss046 |
| dioxygenase | CP000802 | *Escherichia* *coli* HS |
| dioxygenase | CP000036 | *Shigella* *boydii* Sb227 |
| dioxygenase | CP000266 | *Shigella* *flexneri* 5 str. 8401 |
| dioxygenase | ACLI01000099 | *Corynebacterium* *efficiens* YS-314 |
| dioxygenase | BX571866 | *Photorhabdus* *luminescens* subsp. laumondii TTO1 |
| dioxygenase | AB237655 | *Pseudomonas* *putida* plasmid NAH7 |
| dioxygenase | AY887963 | *Pseudomonas* *fluorescens* strain PC20 plasmid pNAH20 |
| dioxygenase | AY125981 | *Pseudomonas* *fluorescens* plasmid pLP6a |
| dioxygenase | AF491307 | *Pseudomonas* *putida* NCIB 9816-4 plasmid pDTG1 |
| dioxygenase | BX640437 | *Bordetella* *bronchiseptica* strain RB50 |
| dioxygenase | AE005674 | *Shigella* *flexneri* 2a str. 301 |
| dioxygenase | AE014073 | *Shigella* *flexneri* 2a str. 2457T |
| dioxygenase | AY208917 | *Pseudomonas* sp. ND6 plasmid pND6-1 |
| dioxygenase | AF036940 | *Ralstonia* sp. U2 |
| dioxygenase | DQ167474 | *Polaromonas* *naphthalenivorans* CJ2 |
| dioxygenase | CP000529 | *Polaromonas* *naphthalenivorans* CJ2 |
| dioxygenase | CP000539 | *Acidovorax* sp. JS42 |
| dioxygenase | CP000077 | *Sulfolobus* *acidocaldarius* DSM 639 |
| dioxygenase | CP000520 | *Mycobacterium* sp. KMS plasmid pMKMS02 |
| dioxygenase | CP000456 | *Arthrobacter* sp. FB24 |
| dioxygenase | CP000518 | *Mycobacterium* sp. KMS |
| dioxygenase | CP000519 | *Mycobacterium* sp. KMS plasmid pMKMS01 |
| dioxygenase | CP000580 | *Mycobacterium* sp. JLS |
| dioxygenase | CP000384 | *Mycobacterium* sp. MCS |
| dioxygenase | CP000385 | *Mycobacterium* sp. MCS |
| dioxygenase | AF331043 | *Arthrobacter* *keyseri* plasmid pRE1 |
| dioxygenase | AP008980 | *Terrabacter* sp. DBF63 plasmid pDBF1 |
| dioxygenase | EF494237 | *Rhodococcus* sp. DK17 plasmid pDK2 |
| dioxygenase | AB048709 | *Rhodococcus* sp. RHA1 |
| dioxygenase | AB154536 | *Rhodococcus* sp. RHA1 |
| dioxygenase | AB154537 | *Rhodococcus* sp. RHA1 |
| putative phthalate dioxygenase | AAR90178 | *Rhodococcus* sp. RHA1 |
| dioxygenase | DQ007994 | *Rhodococcus* sp. TFB |
| dioxygenase | AF546904 | *Mycobacterium* sp. S65 |
| dioxygenase | DQ028634 | *Mycobacterium* *vanbaalenii* PYR-1 |
| biphenyl dioxygenase | EF151283 | *Sphingobium* *yanoikuyae* strain B1 |
| dioxygenase | CP000676 | *Novosphingobium* *aromaticivorans* DSM 12444 |
| dioxygenase | AY502075 | *Rhodococcus* sp. DK17 |
| dioxygenase | AF079317 | *Sphingomonas* *aromaticivorans* plasmid pNL1 |
| dioxygenase | AB272985 | *Rhodococcus* *erythropolis* |
| dioxygenase | AB272986 | *Rhodococcus* sp. HA99 |
| dioxygenase | DQ403247 | *Rhodococcus* sp. R04 |
| dioxygenase | AF119621 | *Pseudomonas* *abietaniphila* BKME-9 |
| dioxygenase | CP000272 | *Paraburkholderia* *xenovorans* LB400 |
| dioxygenase | CP000699 | *Sphingomonas* *wittichii* RW1 |
| dioxygenase | AJ223219 | *Sphingomonas* sp. |
| dioxygenase | AJ223220 | *Sphingomonas* sp. |
| dioxygenase | CP000701 | *Sphingomonas* *wittichii* RW1 |
| dioxygenase | D88021 | *Rhodococcus* *erythropolis* |
| dioxygenase | AB270530 | *Sphingomonas* sp. KA1 |
| dioxygenase | AB110633 | *Rhodococcus* *opacus* |
| dioxygenase | AB102786 | *Cycloclasticus* sp. A5 |
| dioxygenase | AB075242 | *Terrabacter* sp. YK3 |
| dioxygenase | AE015451 | *Pseudomonas* *putida* KT2440 |
| dioxygenase | CP000432 | *Rhodococcus* *jostii* RHA1 plasmid pRHL1 |
| dioxygenase | CP000433 | *Rhodococcus* *jostii* RHA1 plasmid pRHL2 |
| dioxygenase | AY502076 | *Rhodococcus* sp. DK17 plasmid pDK3 |
| dioxygenase | AB257758 | Plasmid pFKY4 |
| dioxygenase | AB257757 | Plasmid pFKY1 |
| dioxygenase | NG_034878 | *Pseudomonas* *putida* plasmid NPL1 |
| napthalene dioxygenase | NG_041567 | *Pseudomonas* *putida* plasmid pAK5 |
| dioxygenase | HM623873 | *Pseudomonas* *chlororaphis* strain SY-02 |
| dioxygenase | JN248563 | *Pseudomonas* sp. MC1 |
| Naphtalene dioxygenase | HM368649 | *Pseudomonas* sp. N1 |
| dioxygenase | CP007510 | *Pseudomonas* *stutzeri* strain 19SMN4 |
| dioxygenase | CP003677 | *Pseudomonas* *stutzeri* CCUG 29243 |
| dioxygenase | HM204990 | *Pseudomonas* *stutzeri* strain NJ |
| dioxygenase | NG_036701 | *Rhodococcus* sp. TFB |
| dioxygenase | CP008952 | *Rhodococcus* *opacus* strain R7 |
| dioxygenase | CP008951 | *Rhodococcus* *opacus* strain R7 |
| dioxygenase | AB024936 | *Rhodococcus* sp. CIR2 |
| dioxygenase | NG035928 | *Rhodococcus* *opacus* plasmid pWK301 |
| dioxygenase | GQ848233 | *Gordonia* sp. CC-NAPH129-6 |
| napthalene dioxygenase | GQ503240 | *Rhodococcus* sp. B13 |
| napthalene dioxygenase | GQ503241 | *Rhodococcus* sp. B2-1 |
| napthalene dioxygenase | GQ503239 | *Rhodococcus* sp. DB11 |
| dioxygenase | CP002385 | *Mycobacterium* *gilvum* Spyr1 |
| NidA | HM049723 | *Mycobacterium* sp. py142 |
| NidA | HM049719 | *Mycobacterium* sp. py137 |
| NidA | HM049714 | Bacterium py120 |
| NidA | HM049725 | *Mycobacterium* sp. py145 |
| NidA | HM049713 | Bacterium py114 |
| NidA | HM049726 | *Mycobacterium* sp. py146 |
| dioxygenase | CP003169 | *Mycobacterium* *rhodesiae* NBB3 |
| dioxygenase | AB626849 | *Mycobacterium* sp. NJS-P |
| dioxygenase | JQ916945 | *Pseudomonas* sp. Jpyr-1 |
| dioxygenase | CP003872 | *Acidovorax* sp. KKS102 |
| dioxygenase | AB706355 | *Comamonas* *testosteroni* genomic DNA |
| dioxygenase | AB546270 | *Acidovorax* sp. KKS102 |
| dioxygenase | CP006704 | *Comamonas* *testosteroni* TK102 |
| dioxygenase | CP008761 | *Burkholderia* *xenovorans* LB400 |
| dioxygenase | JN874407 | *Cupriavidus* sp. SK-4 |
| dioxygenase | FJ715926 | *Pseudomonas* *putida* strain B6-2 |
| dioxygenase | CP003093 | *Pseudoxanthomonas* *spadix* BD-a59 |
| dioxygenase | CP000530 | *Polaromonas* *naphthalenivorans* CJ2 |
| biphenyl dioxygenase | JQ015309 | *Alcanivorax* sp. HA03 |
| biphenyl dioxygenase | AB733643 | *Janibacter* sp. TYM3221 |
| biphenyl dioxygenase | AB609317 | *Janibacter* sp. TYM3221 |
| dioxygenase | EF635855 | *Pseudomonas* *nitroreducens* strain J5-1 |
| dioxygenase | EU825676 | *Bordetella* sp. IITR02 |
| dioxygenase | AM902716 | *Bordetella* *petrii* strain DSM 12804 |
| dioxygenase | CP006979 | *Pseudomonas* *monteilii* SB3101 |
| dioxygenase | CP006978 | *Pseudomonas* *monteilii* SB3078 |
| dioxygenase | CP003734 | *Pseudomonas* *putida* DOT-T1E |
| dioxygenase | CP000712 | *Pseudomonas* *putida* F1 |
| dioxygenase | AB828709 | *Pseudomonas* *putida* |
| dioxygenase | NC_010002 | *Delftia* *acidovorans* SPH-1 |
| dioxygenase | NC_020800 | *Xanthomonas* *axonopodis* Xac29-1 |
| dioxygenase | NC_006834 | *Xanthomonas* *oryzae* pv. oryzae KACC 10331 |

Supplementary Table 5: Dehydrogenase gene sequences database

| **Gene/enzyme** | **Recommended enzyme names (BRENDA, 2017)** | **Accession n.** | **Description** |
| --- | --- | --- | --- |
| TesH (3-ketosteroid-delta1-dehydrogenase) | 3-oxosteroid 1-dehydrogenase, | AB076368 | *Comamonas* *testosteroni* TA441 tesH, tesI, ORF17, ORF18 genes for 3-ketosteriod-delta1-dehydrogenase, 3-ketosteriod-delta4(5alpha)-dehydrogenase, hypothetical protein, complete cds. |
| TesI (3-ketosteroid-delta4(5alpha)-dehydrogenase) | 3-oxo-5alpha-steroid 4-dehydrogenase | AB076368 | *Comamonas* *testosteroni* TA441 tesH, tesI, ORF17, ORF18 genes for 3-ketosteriod-delta1-dehydrogenase, 3-ketosteriod-delta4(5alpha)-dehydrogenase, hypothetical protein, complete cds. |
| 3-beta-hydroxysteroid dehydrogenase ORF62 | 3beta-hydroxy-DELTA5-steroid dehydrogenase | AB474240 | *Comamonas* *testosteroni* TA441 O genes for hypothetical proteins, 3-beta-hydroxysteriod dehydrogenase, hypothetical protein, partial and complete cds, strain:TA441. |
| 3-alpha steroid dehydrogenase (acts as 17β-dehydrogenase) | 3alpha-hydroxysteroid 3-dehydrogenase | AB489116 | *Comamonas* *testosteroni* TA441 genes for 3-ketosteroiod isomerase, 3-alpha steroid dehydrogenase, complete cds, strain: TA441. |
| 3-beta hydroxysteroid dehydrogenase | 3(or 17)beta-hydroxysteroid dehydrogenase | NZ_AKCL01000092 | *Pseudomonas* *putida* SJTE-1 contig000092, whole genome shotgun sequence |
| 3-beta hydroxysteroid dehydrogenase | 3(or 17)beta-hydroxysteroid dehydrogenase | NZ_AKCL01000160 | *Pseudomonas* *putida* SJTE-1 contig000160, whole genome shotgun sequence |
| 3-beta hydroxysteroid dehydrogenase | 3(or 17)beta-hydroxysteroid dehydrogenase | NZ_AFMP01000007 | *Sphingomonas* sp. KC8 contig07, whole genome shotgun sequence. |
| 7-alpha-hydroxysteroid dehydrogenase | 7alpha-hydroxysteroid dehydrogenase | NZ_AFMP01000007 | *Sphingomonas* sp. KC8 contig07, whole genome shotgun sequence. |
| 3-alpha-hydroxysteroid dehydrogenase | 3alpha-hydroxysteroid 3-dehydrogenase | NZ_AFMP01000031 | *Sphingomonas* sp. KC8 contig31, whole genome shotgun sequence. |
| 3-ketosteroid-delta-1-dehydrogenase | 3-oxosteroid 1-dehydrogenase | NZ_AFMP01000031 | *Sphingomonas* sp. KC8 contig31, whole genome shotgun sequence. |
| 3-beta-hydroxysteroid dehydrogenase | 3(or 17)beta-hydroxysteroid dehydrogenase | NC_005027 | *Rhodopirellula* *baltica* SH 1 chromosome, complete genome. |
| 3-keto-5alpha-steroid delta(1)-dehydrogenase KstD3 | 3-oxosteroid 1-dehydrogenase | NC_012490 | *Rhodococcus* *erythropolis* PR4 DNA, complete genome. |
| 3-ketosteroid delta(1)-dehydrogenase KstD2 | 3-oxosteroid 1-dehydrogenase | NC_012490 | *Rhodococcus* *erythropolis* PR4 DNA, complete genome. |
| 3-ketosteroid-delta-1-dehydrogenase | 3-oxosteroid 1-dehydrogenase | NC_012490 | *Rhodococcus* *erythropolis* PR4 DNA, complete genome. |
| 3-ketosteroid delta(1)-dehydrogenase | 3-oxosteroid 1-dehydrogenase | NC_012490 | *Rhodococcus* *erythropolis* PR4 DNA, complete genome. |
| 3-ketosteroid-delta-1-dehydrogenase | 3-oxosteroid 1-dehydrogenase | NC_008269 | *Rhodococcus* *jostii* RHA1 plasmid pRHL1, complete sequence. |
| 3-ketosteroid-delta-1-dehydrogenase | 3-oxosteroid 1-dehydrogenase | NC_008269 | *Rhodococcus* *jostii* RHA1 plasmid pRHL1, complete sequence. |
| 3-ketosteroid-delta-1-dehydrogenase | 3-oxosteroid 1-dehydrogenase | NC_008268 | *Rhodococcus* *jostii* RHA1, complete genome. |
| 3-ketosteroid-delta-1-dehydrogenase | 3-oxosteroid 1-dehydrogenase | NC_008268 | *Rhodococcus* *jostii* RHA1, complete genome. |
| 3-ketosteroid-delta-1-dehydrogenase | 3-oxosteroid 1-dehydrogenase | NC_008268 | *Rhodococcus* *jostii* RHA1, complete genome. |
| 3-ketosteroid-delta-1-dehydrogenase | 3-oxosteroid 1-dehydrogenase | NC_002944 | *Mycobacterium* *avium* subsp. paratuberculosis str. k10, complete genome |
| 3-ketosteroid-delta-1-dehydrogenase | 3-oxosteroid 1-dehydrogenase | NC_002944 | *Mycobacterium* *avium* subsp. paratuberculosis str. k10, complete genome |
| 3-ketosteroid-delta-1-dehydrogenase | 3-oxosteroid 1-dehydrogenase | NC_002944 | *Mycobacterium* *avium* subsp. paratuberculosis str. k10, complete genome |
| 3-ketosteroid-delta-1-dehydrogenase | 3-oxosteroid 1-dehydrogenase | NC_002944 | *Mycobacterium* *avium* subsp. paratuberculosis str. k10, complete genome |
| 3-ketosteroid-delta-1-dehydrogenase | 3-oxosteroid 1-dehydrogenase | NC_016946 | *Mycobacterium* *intracellulare* ATCC 13950, complete genome. |
| 3-ketosteroid-delta-1-dehydrogenase | 3-oxosteroid 1-dehydrogenase | NC_016946 | *Mycobacterium* *intracellulare* ATCC 13950, complete genome. |
| 3-ketosteroid-delta-1-dehydrogenase | 3-oxosteroid 1-dehydrogenase | NC_016946 | *Mycobacterium* *intracellulare* ATCC 13950, complete genome. |
| 3-ketosteroid-delta-1-dehydrogenase | 3-oxosteroid 1-dehydrogenase | NC_016946 | *Mycobacterium* *intracellulare* ATCC 13950, complete genome. |
| 3-ketosteroid-delta-1-dehydrogenase | 3-oxosteroid 1-dehydrogenase | NC_008596 | *Mycobacterium* *smegmatis* str. MC2 155 chromosome, complete genome. |
| 3-ketosteroid-delta-1-dehydrogenase | 3-oxosteroid 1-dehydrogenase | NC_008596 | *Mycobacterium* *smegmatis* str. MC2 155 chromosome, complete genome. |
| 3-ketosteroid-delta-1-dehydrogenase | 3-oxosteroid 1-dehydrogenase | NC_008596 | *Mycobacterium* *smegmatis* str. MC2 155 chromosome, complete genome. |
| 3-ketosteroid-delta-1-dehydrogenase | 3-oxosteroid 1-dehydrogenase | NC_008596 | *Mycobacterium* *smegmatis* str. MC2 155 chromosome, complete genome. |
| 3-ketosteroid-delta-1-dehydrogenase | 3-oxosteroid 1-dehydrogenase | NC_008611 | *Mycobacterium* *ulcerans* Agy99 chromosome, complete genome. |
| 3-ketosteroid-delta-1-dehydrogenase | 3-oxosteroid 1-dehydrogenase | NC_008611 | *Mycobacterium* *ulcerans* Agy99 chromosome, complete genome. |
| 3-beta-hydroxy-delta(5)-steroid dehydrogenase | 3beta-hydroxy-DELTA5-steroid dehydrogenase | NC_013209 | *Acetobacter* *pasteurianus* IFO 3283-01 DNA, complete genome. |
| 3-beta-hydroxy-delta(5)-steroid dehydrogenase | 3beta-hydroxy-DELTA5-steroid dehydrogenase | NZ_AFSD01000001 | *Agrobacterium* *tumefaciens* F2 chromosome C c1, whole genome shotgun sequence. |
| 3-beta-hydroxy-delta(5)-steroid dehydrogenase | 3beta-hydroxy-DELTA5-steroid dehydrogenase | NC_009667 | *Ochrobactrum* *anthropi* ATCC 49188 chromosome 1, complete sequence. |
| 3-beta-hydroxy-delta(5)-steroid dehydrogenase | 3beta-hydroxy-DELTA5-steroid dehydrogenase | NC_007761 | *Rhizobium* *etli* CFN 42, complete genome. |
| 3-beta-hydroxy-delta(5)-steroid dehydrogenase | 3beta-hydroxy-DELTA5-steroid dehydrogenase | NC_010505 | *Methylobacterium* *radiotolerans* JCM 2831, complete genome. |
| 3-beta-hydroxy-delta(5)-steroid dehydrogenase | 3beta-hydroxy-DELTA5-steroid dehydrogenase | NZ_KB900701 | *Bradyrhizobium* *elkanii* USDA 76 BraelDRAFT_scaffold1.1, whole genome shotgun sequence. |
| 3-beta-hydroxy-delta(5)-steroid dehydrogenase | 3beta-hydroxy-DELTA5-steroid dehydrogenase | NC_008783 | *Bartonella* *bacilliformis* KC583, complete genome. |
| KsdD-like steroid dehydrogenase | 3-oxosteroid 1-dehydrogenase | NC_000962 | *Mycobacterium* *tuberculosis* H37Rv, complete genome. |
| 3-beta-hydroxy-delta(5)-steroid dehydrogenase | 3beta-hydroxy-DELTA5-steroid dehydrogenase | NC_007643 | *Rhodospirillum* *rubrum* ATCC 11170 chromosome, complete genome. |
| 3-oxo-5-alpha-steroid 4-dehydrogenase | 3-oxo-5alpha-steroid 4-dehydrogenase (NADP+) | NC_009614 | *Bacteroides* *vulgatus* ATCC 8482, complete genome. |
| delta 4, 5-alpha steroid dehydrogenase | 3-oxo-5alpha-steroid 4-dehydrogenase (NADP+) | NZ_JH604622 | *Pseudomonas* *fragi* B25 Scaffold1, whole genome shotgun sequence. |
| 3-oxo-5-alpha-steroid 4-dehydrogenase | 3-oxo-5alpha-steroid 4-dehydrogenase (NADP+) | NC_006347 | *Bacteroides* *fragilis* YCH46 DNA, complete genome. |
| NAD(P)H steroid dehydrogenase | 3alpha-hydroxysteroid 3-dehydrogenase | NC_003902 | *Xanthomonas* *campestris* pv. campestris str. ATCC 33913 chromosome, complete genome. |
| NAD(P)H steroid dehydrogenase | 3alpha-hydroxysteroid 3-dehydrogenase | NC_003902 | *Xanthomonas* *campestris* pv. campestris str. ATCC 33913 chromosome, complete genome. |
| 3-oxo-5-alpha-steroid 4-dehydrogenase | 3-oxo-5alpha-steroid 4-dehydrogenase (NADP+) | NC_004663 | *Bacteroides* *thetaiotaomicron* VPI-5482 chromosome, complete genome. |
| Putative steroid dehydrogenase | 3beta-hydroxy-DELTA5-steroid dehydrogenase (possibly) | NC_010397 | *Mycobacterium* *abscessus* chromosome, complete sequence. |
| 3-beta hydroxysteroid dehydrogenase | 3(or 17)beta-hydroxysteroid dehydrogenase | NC_004129 | *Pseudomonas* *protegens* Pf-5, complete genome. |
| 7-alpha-hydroxysteroid dehydrogenase | Recommended names (BRENDA) | NC_002695 | *Escherichia* *coli* O157:H7 str. Sakai chromosome, complete genome. |
| 3-beta hydroxysteroid dehydrogenase/isomerase family protein | 3(or 17)beta-hydroxysteroid dehydrogenase | NC_003997 | *Bacillus* *anthracis* str. Ames chromosome, complete genome. |
| 3-ketosteroid-delta-1-dehydrogenase | 3-oxosteroid 1-dehydrogenase | NC_010382 | *Lysinibacillus* *sphaericus* C3-41, complete genome. |
| 7-alpha-hydroxysteroid dehydrogenase | 7alpha-hydroxysteroid dehydrogenase | NC_007618 | *Brucella* *melitensis* biovar Abortus 2308 chromosome I, complete sequence, strain 2308. |
| 7-alpha-hydroxysteroid dehydrogenase | 7alpha-hydroxysteroid dehydrogenase | NC_009850 | *Arcobacter* *butzleri* RM4018, complete genome. |
| 7-alpha-hydroxysteroid dehydrogenase | 7alpha-hydroxysteroid dehydrogenase | NC_011751 | *Escherichia* *coli* UMN026 chromosome, complete genome. |
| 7-alpha-hydroxysteroid dehydrogenase | 7alpha-hydroxysteroid dehydrogenase | NC_011750 | *Escherichia* *coli* IAI39 chromosome, complete genome. |
| 7-alpha-hydroxysteroid dehydrogenase | 7alpha-hydroxysteroid dehydrogenase | NC_012039 | *Campylobacter* *lari* RM2100, complete genome. |
| 7-alpha-hydroxysteroid dehydrogenase | 7alpha-hydroxysteroid dehydrogenase | NZ_CP007181 | *Campylobacter* *coli* RM4661, complete genome. |
| 7-alpha-hydroxysteroid dehydrogenase | 7alpha-hydroxysteroid dehydrogenase | NZ_KB849749 | *Acinetobacter* *radioresistens* DSM 6976 = NBRC 102413 = CIP 103788 acLrZ-supercont1.9, whole genome shotgun sequence. |
| 7-alpha-hydroxysteroid dehydrogenase | 7alpha-hydroxysteroid dehydrogenase | NC_008599 | *Campylobacter* *fetus* subsp. fetus 82-40, complete genome. |
| 7-alpha-hydroxysteroid dehydrogenase | 7alpha-hydroxysteroid dehydrogenase | NC_000915 | *Helicobacter* *pylori* 26695 chromosome, complete genome. |
| ORF18 (likely 3alpha-dehydrogenase ) 1b | 3alpha-hydroxysteroid 3-dehydrogenase | AB076368 | *Comamonas* *testosteroni* TA441 tesH, tesI, ORF17, ORF18 genes for 3-ketosteriod-delta1-dehydrogenase, 3-ketosteriod-delta4(5alpha)-dehydrogenase, hypothetical protein, complete cds. |
| TesR (regulator gene for degradation) (likely Delta1-dehydrogenase ) | 3-oxosteroid 1-dehydrogenase | AB186487 | *Comamonas* *testosteroni* TA441 degradation gene cluster (ORF genes and tesR gene), complete cds. |
| 3-ketosteroid-delta 4(5 alpha)-dehydrogenase | 3-oxosteroid 1-dehydrogenase | L23428 | *Comamonas* *testosteroni* ATCC 17410 delta 4, 5-alpha steroid dehydrogenase gene, complete cds. |
| 3-oxosteriod 1-dehydrogenase | 3-oxosteriod 1-dehydrogenase | JAFFSZ010000000 | *R*.*equi* ATCC13557 (rast output for spades assembly) |
| 17beta hydroxysteriod dehydrogenase | 17beta hydroxysteriod dehydrogenase | FN563149.1 | *Rhodococcus* *equi* 103S chromosome. |

Supplementary Table 6: Dehydrogenase gene sequences taken from Kisiela et al., (2012).

| **Gene/enzyme** | **RefSeq ID** | **Description** |
| --- | --- | --- |
| 3-ketosteroid-Δ1-dehydrogenase | YP_907615 | *Mycobacterium* *ulcerans* Agy99 |
| 3-ketosteroid-Δ1-dehydrogenase | ZP_04751485 | *Mycobacterium* *kansasii* ATCC 12478 |
| 3-ketosteroid-Δ1-dehydrogenase | YP_001073300 | *Mycobacterium* sp. JLS |
| 3-ketosteroid-Δ1-dehydrogenase | YP_641822 | *Mycobacterium* sp. MCS |
| 3-ketosteroid-Δ1-dehydrogenase | YP_940731 | *Mycobacterium* sp. KMS |
| 3-ketosteroid-Δ1-dehydrogenase | YP_890167 | *Mycobacterium* *smegmatis* str. MC2 155 |
| 3-ketosteroid-Δ1-dehydrogenase | YP_956014 | *Mycobacterium* *vanbaalenii* PYR-1 |
| 3-ketosteroid-Δ1-dehydrogenase | YP_001132789 | *Mycobacterium* *gilvum* PYR-GCK |
| Probable dehydrogenase | YP_001701374 | *Mycobacterium* *abscessus* |
| 3-ketosteroid-Δ1-dehydrogenase | YP_116669 | *Nocardia* *farcinica* IFM 10152 |
| 3-ketosteroid-Δ1-dehydrogenase | YP_704476 | *Rhodococcus* *jostii* RHA1 |
| 3-ketosteroid-Δ1-dehydrogenase | YP_002781639 | *Rhodococcus* *opacus* B4 |
| 3-ketosteroid-Δ1-dehydrogenase | ZP_04388196 | *Rhodococcus* *erythropolis* SK121 |
| 3-ketosteroid-Δ1-dehydrogenase | YP_002764184 | *Rhodococcus* *erythropolis* PR4 |
| 3-ketosteroid-Δ1-dehydrogenase | ZP_05478502 | *Streptomyces* sp. AA4 |
| 3-ketosteroid-Δ1-dehydrogenase | YP_923969 | *Nocardioides* sp. JS614 |
| 3-ketosteroid-Δ1-dehydrogenase | ZP_06607635 | *Aeromicrobium* *marinum* DSM 15272 |
| 3-ketosteroid-Δ1-dehydrogenase | YP_001413067 | *Parvibaculum* *lavamentivorans* DS-1 |
| 3-ketosteroid-Δ1-dehydrogenase | YP_001696275 | *Lysinibacillus* *sphaericus* C3-41 |
| 3-ketosteroid-Δ1-dehydrogenase | ZP_01725238 | *Bacillus* sp. B14905 |
| putative FAD binding domain protein | ZP_05095058 | marine gamma proteobacterium HTCC2148 |
| Fumarate reductase/succinate dehydrogenase flavoprotein subunit | ZP_01626281 | marine gamma proteobacterium HTCC2080 |
| putative FAD binding domain protein | ZP_05094533 | marine gamma proteobacterium HTCC2148 |
| fumarate reductase/succinate dehydrogenase flavoprotein domain protein | ZP_03544409 | *Comamonas* *testosteroni* KF-1 |
| hypothetical protein | ZP_03607292 | *Methanobrevibacter* *smithii* DSM 2375 |
| short chain dehydrogenase | YP_447755 | *Methanosphaera* *stadtmanae* DSM 3091 |
| hypothetical protein | ZP_03636608 | *Holdemania* *filiformis* DSM 12042 |
| short-chain dehydrogenase/reductase SDR | YP_003182859 | *Eggerthella* *lenta* DSM 2243 |
| hypothetical protein | ZP_03759971 | *Clostridium* *asparagiforme* DSM 15981 |
| short chain dehydrogenase/reductase | ZP_04672086 | *Clostridiales* *bacterium* 1_7_47_FAA |
| hypothetical protein | ZP_03777187 | *Clostridium* *hylemonae* DSM 15053 |
| hypothetical protein | ZP_02432210 | *Clostridium* *scindens* ATCC 35704 |
| hypothetical protein | ZP_03293133 | *Clostridium* *hiranonis* DSM13275 |
| hypothetical protein | ZP_02236388 | *Dorea* *formicigenerans* ATCC 27755 |
| hypothetical protein | ZP_03463615 | [*Bacteroides*] *pectinophilus* ATCC43243 |
| Oxidoreductase, short chain dehydrogenase/reductase family protein | ZP_01772477 | *Collinsella* *aerofaciens* ATCC 25986 |
| hypothetical protein | ZP_03296838 | *Collinsella* *stercoris* DSM 13279 |
| hypothetical protein | ZP_03799118 | *Coprococcus* *comes* ATCC 27758 |
| hypothetical protein | ZP_02027406 | *Eubacterium* *ventriosum* ATCC27560 |
| short chain dehydrogenase | ZP_05744752 | *Lactobacillus* *antri* DSM 16041 |
| oxidoreductase, short chain dehydrogenase/reductase family protein | ZP_03682514 | *Catenibacterium* *mitsuokai* DSM 15897 |
| hypothetical protein | ZP_02075484 | *Clostridium* sp. L2-50 |
| hypothetical protein | ZP_02205767 | *Coprococcus* *eutactus* ATCC 27759 |
| 3-oxoacyl-(acyl-carrier-protein) reductase | ZP_06113400 | *Clostridium* *hathewayi* DSM 13479 |
| hypothetical protein | ZP_02429777 | *Clostridium* *ramosum* DSM 1402 |
| short-chain dehydrogenase | ZP_04565678 | *Coprobacillus* sp. D7 |
| short chain dehydrogenase/reductase | YP_001274304 | *Methanobrevibacter* *smithii* ATCC 35061 |
| short-chain dehydrogenase | ZP_05976067 | *Methanobrevibacter* *smithii* DSM 2374 |
| hypothetical protein | YP_637479 | *Mycobacterium* sp. MCS |
| hypothetical protein | YP_936319 | *Mycobacterium* sp. KMS |
| hypothetical protein | YP_001537700 | *Salinispora* *arenicola* CNS-205 |
| hypothetical protein | YP_001159497 | *Salinispora* *tropica* CNB-440 |
| hypothetical protein | ZP_05478503 | *Streptomyces* sp. AA4 |
| hypothetical protein | YP_116670 | *Nocardia* *farcinica* IFM 10152 |
| Probable dehydrogenase | YP_001701375 | *Mycobacterium* *abscessus* |
| hypothetical protein | YP_908262 | *Mycobacterium* *ulcerans* Agy99 |
| hypothetical protein | YP_001848529 | *Mycobacterium* *marinum* M |
| hypothetical protein | ZP_04747468 | *Mycobacterium* *kansasii* ATCC12478 |
| putative 3-ketosteroid delta(4)(5alpha)-dehydrogenase | YP_002764185 | *Rhodococcus* *erythropolis* PR4 |
| 3-ketosteroid-delta4 dehydrogenase | ZP_04388024 | *Rhodococcus* *erythropolis* SK121 |
| fumarate reductase/succinate dehydrogenase | YP_003277400 | *Comamonas* *testosteroni* CNB-2 |
| fumarate reductase/succinate dehydrogenase flavoprotein domain protein | ZP_03544408 | *Comamonas* *testosteroni* KF-1 |
| hypothetical protein | YP_728803 | *Ralstonia* *eutropha* H16 |
| hypothetical protein | YP_001796921 | *Cupriavidus* *taiwanensis* LMG 19424 |
| hypothetical protein | YP_001166106 | *Novosphingobium* *aromaticivorans* DSM 12444 |
| hypothetical protein | YP_001263819 | *Sphingomonas* *wittichii* RW1 |
| hypothetical protein | YP_001262358 | *Sphingomonas* *wittichii* RW1 |
| fumarate reductase/succinate dehydrogenase flavoprotein domain | ZP_03265787 | *Burkholderia* sp. H160 |
| hypothetical protein | YP_713249 | *Frankia* *alni* ACN14a |
| hypothetical protein | YP_001135820 | *Mycobacterium* *gilvum* PYR-GCK |
| hypothetical protein | YP_887193 | *Mycobacterium* *smegmatis* str. MC2 155 |
| hypothetical protein | YP_001068597 | *Mycobacterium* sp. JLS |
| fumarate reductase/succinate dehydrogenase | YP_003277399 | *Comamonas* *testosteroni* CNB-2 |
| 3-ketosteroid-delta1-dehydrogenase | YP_340631 | *Pseudoalteromonas* *haloplanktis* TAC125 |
| fumarate reductase/succinate dehydrogenase flavoprotein domain-containing protein | YP_001674315 | *Shewanella* *halifaxensis* HAW-EB4 |
| fumarate reductase/succinate dehydrogenase flavoprotein domain-containing protein | YP_001501980 | *Shewanella* *pealeana* ATCC 700345 |
| fumarate reductase/succinate dehydrogenase flavoprotein, N-terminal:FAD dependent oxidoreductase | YP_295753 | *Ralstonia* *eutropha* JMP134 |
| 3-oxosteroid 1-dehydrogenase | YP_728801 | *Ralstonia* *eutropha* H16 |
| 3-oxosteroid 1-dehydrogenase | YP_001796919 | *Cupriavidus* *taiwanensis* LMG 19424 |
| fumarate reductase/succinate dehydrogenase flavoprotein domain protein | ZP_02893600 | *Burkholderia* *ambifaria* IOP40-10 |
| fumarate reductase/succinate dehydrogenase flavoprotein subunit | YP_372172 | *Burkholderia* *lata* |
| putative FAD-binding component for oxidoreductase | YP_002234257 | *Burkholderia* *cenocepacia* J2315 |
| 3-oxosteroid 1-dehydrogenase | YP_001262337 | *Sphingomonas* *wittichii* RW1 |
| fumarate reductase/succinate dehydrogenase flavoprotein domain-containing protein | YP_001262329 | *Sphingomonas* *wittichii* RW1 |
| fumarate reductase/succinate dehydrogenase flavoprotein domain-containing protein | YP_001166117 | *Novosphingobium* *aromaticivorans* DSM 12444 |
| fumarate reductase/succinate dehydrogenase flavoprotein domain-containing protein | YP_001263842 | *Sphingomonas* *wittichii* RW1 |
| fumarate reductase/succinate dehydrogenase flavoprotein domain-containing protein | YP_001166011 | *Novosphingobium* *aromaticivorans* DSM 12444 |
| 3-ketosteroid-delta-1-dehydrogenase | YP_640946 | *Mycobacterium* sp. MCS |
| 3-ketosteroid-delta-1-dehydrogenase | YP_939840 | *Mycobacterium* sp. KMS |
| fumarate reductase/succinate dehydrogenase flavoprotein domain-containing protein | YP_003301095 | *Thermomonospora* *curvata* DSM 43183 |
| 3-ketosteroid-delta-1-dehydrogenase | YP_002783051 | *Rhodococcus* *opacus* B4 |
| 3-ketosteroid-delta-1-dehydrogenase | YP_705733 | *Rhodococcus* *jostii* RHA1 |
| 3-oxosteroid 1-dehydrogenase | ZP_04387793 | *Rhodococcus* *erythropolis* SK121 |
| 3-ketosteroid-delta-1-dehydrogenase | YP_702446 | *Rhodococcus* *jostii* RHA1 |
| 3-ketosteroid-delta-1-dehydrogenase | YP_002779402 | *Rhodococcus* *opacus* B4 |
| 3-ketosteroid-delta-1-dehydrogenase | YP_001159498 | *Salinispora* *tropica* CNB-440 |
| 3-ketosteroid-delta-1-dehydrogenase | YP_001537701 | *Salinispora* *arenicola* CNS-205 |
| 3-ketosteroid-delta-1-dehydrogenase | YP_001159451 | *Salinispora* *tropica* CNB-440 |
| 3-ketosteroid-delta-1-dehydrogenase | YP_001537652 | *Salinispora* *arenicola* CNS-205 |
| 3-ketosteroid-delta-1-dehydrogenase | NP_822771 | *Streptomyces* *avermitilis* MA-4680 = NBRC 14893 |
| 3-ketosteroid-delta-1-dehydrogenase | ZP_05016500 | *Streptomyces* *sviceus* ATCC 29083 |
| 3-ketosteroid-delta-1-dehydrogenase | ZP_05533430 | *Streptomyces* *viridochromogenes* DSM 40736 |
| oxidoreductase | YP_003491889 | *Streptomyces* *scabiei* 87.22 |
| 3-ketosteroid-delta-1-dehydrogenase | YP_003115795 | *Catenulispora* *acidiphila* |
| 3-ketosteroid-delta-1-dehydrogenase | ZP_05001264 | *Streptomyces* sp. Mg1 |
| 3-ketosteroid-delta-1-dehydrogenase | ZP_05504907 | *Streptomyces* sp. C |
| succinate dehydrogenase/fumarate reductase flavoprotein subunit | ZP_04025274 | *Tsukamurella* *paurometabola* DSM 20162 |
| succinate dehydrogenase/fumarate reductase flavoprotein subunit | YP_003272100 | *Gordonia* *bronchialis* DSM 43247 |
| succinate dehydrogenase/fumarate reductase flavoprotein subunit | ZP_04387329 | *Rhodococcus* *erythropolis* SK121 |
| 3-ketosteroid delta(1)-dehydrogenase | YP_002766394 | *Rhodococcus* *erythropolis* PR4 |
| 3-ketosteroid-delta-1-dehydrogenase | NP_959464 | *Mycobacterium* *avium* subsp. paratuberculosis K-10 |
| 3-ketosteroid-delta-1-dehydrogenase | YP_879904 | *Mycobacterium* *avium* 104 |
| 3-ketosteroid-delta-1-dehydrogenase | ZP_05215097 | *Mycobacterium* *avium* subsp. avium ATCC 25291 |
| 3-ketosteroid-delta-1-dehydrogenase | ZP_05227620 | *Mycobacterium* *intracellulare* ATCC 13950 |
| 3-ketosteroid-delta-1-dehydrogenase | YP_001853283 | *Mycobacterium* *marinum* M |

**Supplementary Material References List**

Chen, Y.-L., Fu, H.-Y., Lee, T.-H., Shih, C.-J., Huang, L., Wang, Y.-S., et al. (2018). Estrogen degraders and estrogen degradation pathway identified in an activated sludge. *Appl. Environ. Microbiol.* 84:e00001-18. doi: 10.1128/AEM. 00001-18

Coombe, R. G., Tsong, Y. Y., Hamilton, P. B., and Sih, C. J. (1966). Mechanisms of steroid oxidation by microorganisms: X. Oxidative Cleavage of estrone. *J. Biol. Chem.* 241, 1587–1595.

Fahrbach, M., Kuever, J., Meinke, R., Kämpfer, P., and Hollender, J. (2006). *Denitratisoma* *oestradiolicum* gen. nov., sp. nov., a 17beta-oestradiol-degrading, denitrifying betaproteobacterium. *Int. J. Syst. Evol. Microbiol*. 56, 1547–1552. doi: 10.1099/ijs.0.63672-0

Fahrbach, M., Kuever, J., Remesch, M., Huber, B. E., Kämpfer, P., Dott, W., et al. (2008). *Steroidobacter denitrificans* gen. nov., sp. nov., a steroidal hormone-degrading gammaproteobacterium. *Int. J. Syst. Evol. Microbiol*. 58, 2215–2223. doi: 10.1099/ijs.0.65342-0

Fujii, K., Kikuchi, S., Satomi, M., Ushio-Sata, N., and Morita, N. (2002). Degradation of 17beta-estradiol by a gram-negative bacterium isolated from activated sludge in a sewage treatment plant in Tokyo, Japan. *Appl. Environ. Microbiol.* 68, 2057–2060. doi: 10.1128/AEM.68.4.2057-2060.2002

Fujii, K., Satomi, M., Morita, N., Motomura, T., Tanaka, T., and Kikuchi, S. (2003). *Novosphingobium tardaugens* sp. nov., an oestradiol-degrading bacterium isolated from activated sludge of a sewage treatment plant in Tokyo. *Int. J. Syst. Evol. Microbiol.* 53, 47–52. doi: 10.1099/ijs.0.02301-0

Gaulke, L. S., Strand, S. E., Kalhorn, T. F., and Stensel, H. D. (2008). 17alpha-ethinylestradiol transformation via abiotic nitration in the presence of ammonia oxidizing bacteria. *Environ. Sci. Technol.* 42, 7622–7627. doi: 10.1021/ es801503u

Haiyan, R., Shulan, J., Ud Din Ahmad, N., Dao, W., and Chengwu, C. (2007). Degradation characteristics and metabolic pathway of 17alpha-ethynylestradiol by *Sphingobacterium* sp. JCR5. *Chemosphere* 66, 340–346. doi: 10.1016/j. chemosphere.2006.04.064

Hashimoto, T., Onda, K., Morita, T., Begum, L., Tada, K., Miya, A., et al. (2010). Contribution of the estrogen-degrading bacterium *Novosphingobium* sp. strain JEM-1 to estrogen removal in wastewater treatment. *J. Environ. Eng.* 136, 890–896. doi: 10.1061/(ASCE)EE.1943-7870.0000218

Hsiao, T.-H., Chen, Y.-L., Meng, M., Chuang, M.-R., Horinouchi, M., Hayashi, T., et al. (2020). Genetic and metabolite biomarkers reveal actinobacteria-mediated estrogen biodegradation in urban estuarine sediment. *bioRxiv* [Preprint]. doi: 10.1101/2020.10.07.329094v1

Hsiao, T.-H., Chen, Y.-L., Meng, M., Chuang, M.-R., Horinouchi, M., Hayashi, T., et al. (2021). Mechanistic and phylogenetic insights into actinobacteria-mediated oestrogen biodegradation in urban estuarine sediments. *Microb. Biotechnol.* doi: 10.1111/1751-7915.13798 [Epub ahead of print].

Hu, A., He, J., Chu, K.-H., and Yu, C.-P. (2011). Genome sequence of the 17β-estradiol-utilizing bacterium *Sphingomonas* strain KC8. *J. Bacteriol.* 193, 4266–4267. doi: 10.1128/JB.05356-11

Ibero, J., Sanz, B., Galan, E., Diaz, J., and Garcia, L. (2019). High-quality whole-genome sequence of an estradiol-degrading strain, *Novosphingobium tardaugens* NBRC 16725. *Microbiol. Resour. Announc.* 8:e01715-18. doi: 10. 1128/MRA.01715-18

Ibero, J., Galán, B., Rivero-Buceta, V., and García, J. L. (2020). Unraveling the 17β-estradiol degradation pathway in *Novosphingobium tardaugens* NBRC 16725. *Front. Microbiol*. 11:588300. doi: 10.3389/fmicb.2020.588300

Jiang, L., Yang, J., and Chen, J. (2010). Isolation and characteristics of 17betaestradiol-degrading *Bacillus* spp. strains from activated sludge. *Biodegradation* 21, 729–736. doi: 10.1007/s10532-010-9338-z

Ke, J., Zhuang, W., Gin, K. Y., Reinhard, M., Hoon, L. T., and Tay, J. H. (2007). Characterization of estrogen-degrading bacteria isolated from an artificial sandy aquifer with ultrafiltered secondary effluent as the medium. *Appl. Microbiol. Biotechnol.* 75, 1163–1171. doi: 10.1007/s00253-007-0923-y

Kisiela, M., Skarka, A., Ebert, B., and Maser, E. (2012). Hydroxysteroid dehydrogenases (HSDs) in bacteria: a bioinformatic perspective. *J. Steroid. Biochem. Mol. Biol*. 129, 31–46. doi: 10.1016/j.jsbmb.2011.08.002

Kurisu, F., Ogura, M., Saitoh, S., Yamazoe, A., and Yagi, O. (2010). Degradation of natural estrogen and identification of the metabolites produced by soil isolates of *Rhodococcus* sp. and *Sphingomonas* sp. *J. Biosci. Bioeng.* 109, 576–582. doi: 10.1016/j.jbiosc.2009.11.006

Larcher, S., and Yargeau, V. (2013). Biodegradation of 17alpha-ethinylestradiol by heterotrophic bacteria. *Environ. Pollut.* 173, 17–22. doi: 10.1016/j.envpol.2012. 10.028

Li, S., Liu, J., Sun, M., Ling, W., and Zhu, X. (2017). Isolation, characterization, and degradation performance of the 17β-estradiol-degrading bacterium *Novosphingobium* sp. E2S. *Int. J. Environ.* *Res. Public Health* 14:115. doi: 10. 3390/ijerph14020115

Li, S., Liu, J., Williams, M. A., Wanting, L., Sun, K., Lu, C., et al. (2020). Metabolism of 17β-estradiol by *Novosphingobium* sp. ES2-1 as probed via HRMS combined with 13C3-labeling. *J. Hazard Mater.* 389:121875. doi: 10.1016/j.jhazmat.2019. 121875

Li, S., Sun, K., Yan, X., Lu, C., Waigi, M. G., Liu, J., et al. (2021). Identification of novel catabolic genes involved in 17β-estradiol degradation by *Novosphingobium* sp. ES2-1. *Environ. Microbiol.* 23, 2550–2563. doi: 10.1111/ 1462-2920.15475

Liang, R., Liu, H., Tao, F., Liu, Y., Ma, C., Liu, X., et al. (2012). Genome sequence of *Pseudomonas putida* strain SJTE-1, a bacterium capable of degrading estrogens and persistent organic pollutants. *J. Bacteriol.* 194, 4781–4782. doi: 10.1128/JB. 01060-12

Ma, C., Qin, D., Sun, Q., Zhang, F., Liu, H., and Yu, C. P. (2016). Removal of environmental estrogens by bacterial cell immobilization technique. *Chemosphere* 144, 607–614. doi: 10.1016/j.chemosphere.2015.09.014

Muller, M., Patureau, D., Godon, J. J., Delgenes, J. P., and Hernandez-Raquet, G. (2010). Molecular and kinetic characterization of mixed cultures degrading natural and synthetic estrogens. *Appl. Microbiol. Biotechnol.* 85, 691–701. doi: 10.1007/s00253-009-2160-z

O’Grady, D., Evangelista, S., and Yargeau, V. (2009). Removal of aqueous 17α-ethinylestradiol by *Rhodococcus* species. *Environ. Eng. Sci.* 26, 1393–1400. doi: 10.1016/j.envpol.2012.10.028

Ojanotko-Harri, A., Nikkari, T., Harri, M. P., and Paunio, K. U. (1990). Metabolism of progesterone and testosterone by *Bacillus cereus* strain Socransky 67 and *Streptococcus mutans* strain Ingbritt. *Oral Microbiol. Immunol.* 5, 237–239. doi: 10.1111/j.1399-302X.1990.tb00653.x

Pauwels, B., Wille, K., Noppe, H., De Brabander, H., Van De Wiele, T., Verstraete, W et al., (2008). 17alpha-ethinylestradiol cometabolism by bacteria degrading estrone, 17beta-estradiol and estriol. *Biodegradation*, 19, 683-693. doi: 10.1007/s10532-007-9173-z

Pratush, A., Yang, Q., Peng, T., Huang, T., and Hu, Z. (2020). Identification of non-accumulating intermediate compounds during estrone (E1) metabolism by a newly isolated microbial strain BH2-1 from mangrove sediments of the South China Sea. *Environ. Sci. Pollut. Res.* 27, 5097–5107. doi: 10.1007/s11356-019- 06894-1

Qin, D., Ma, C., Hu, A., Zhang, F., and Hu, H. (2016). *Altererythrobacter estronivorus* sp. nov., an estrogen-degrading strain isolated from Yundang lagoon of Xiamen city in China. *Curr. Microbiol.* 72, 634–640. doi: 10.1007/ s00284-016-0995-y

Qin, D., Ma, C., Lv, M., and Yu, C.-P. (2020). *Sphingobium estronivorans* sp. nov. and *Sphingobium bisphenolivorans* sp. nov., isolated from a wastewater treatment plant. *Int. J. Syst. Evol. Microbiol.* 70, 1822–1829. doi: 10.1099/ijsem. 0.003978

Qiu, Q., Wang, P., Kang, H., Tian, K., and Huo, H. (2019). Genomic analysis of a new estrogen-degrading bacterial strain, *Acinetobacter* sp. DSSKY-A-001. *Int. J. Genomics* 2019:2804134. doi: 10.1155/2019/2804134

Roh, H., and Chu, K. H. (2010). A 17beta-estradiol-utilizing bacterium, *Sphingomonas* strain KC8: part I - characterization and abundance in wastewater treatment plants. *Environ. Sci. Technol*. 44, 4943–4950. doi: 10.1021/ es1001902

Sabirova, J. S., Cloetens, L. F., Vanhaecke, L., Forrez, I., Verstraete, W., and Boon, N. (2008). Manganese-oxidizing bacteria mediate the degradation of 17alpha-ethinylestradiol. *Microb. Biotechnol*. 1, 507–512. doi: 10.1111/j.1751- 7915.2008.00051.x

Sang, Y., Xiong, G., and Maser, E. (2012). Identification of a new steroid degrading bacterial strain H5 from the Baltic Sea and isolation of two estradiol inducible genes. *J. Steroid Biochem. Mol. Biol.* 129, 22–30. doi: 10.1016/j.jsbmb.2011. 01.018

Shi, J., Fujisawa, S., Nakai, S., and Hosomi, M. (2004). Biodegradation of natural and synthetic estrogens by nitrifying activated sludge and ammonia-oxidizing bacterium *Nitrosomonas europaea. Water. Res.* 38, 2322-2329. doi: 10.1016/j.watres.2004.02.022

Skotnica-Pitak, J., Khunjar, W. O., Love, N. G., and Aga, D. S. (2009). Characterization of metabolites formed during the biotransformation of 17α-ethinylestradiol by *Nitrosomonas europaea* in batch and continuous flow bioreactors. *Environ. Sci. Technol.* 43, 3549–3555. doi: 10.1021/es80 26659

Song, X., Xu, Y., Li, G., Zhang, Y., Huang, T., and Hu, Z. (2011). Isolation, characterization of *Rhodococcus* sp. P14 capable of degrading high-molecularweight polycyclic aromatic hydrocarbons and aliphatic hydrocarbons. *Mar. Pollut. Bull.* 62, 2122–2128. doi: 10.1016/j.marpolbul.2011.07.013

Tian, K., Meng, F., Meng, Q., Gao, Y., Zhang, L., Wang, L., et al. (2020). The analysis of estrogen-degrading and functional metabolism genes in *Rhodococcus equi* DSSKP-R-001. *Int. J. Genomics* 2020, 9369182–93691213. doi: 10.1155/ 2020/9369182

Wang, Y., Zhao, X., Tian, K., Meng, F., Zhou, D., Xu, X., et al. (2020a). Identification and genome analysis of a novel 17β-estradiol degradation bacterium, *Lysinibacillus sphaericus* DH-B01. *3 Biotech*. 10:166. doi: 10.1007/ s13205-020-2155-0

Wang, P. H., Chen, Y. L., Wei, S. T., Wu, K., Lee, T. H., Wu, T. Y., et al. (2020b). Retroconversion of estrogens into androgens by bacteria via a cobalamin-mediated methylation. *Proc. Natl. Acad. Sci. U.S.A.* 117, 1395–1403. doi: 10. 1073/pnas.1914380117

Weber, S., Leuschner, P., Kampfer, P., Dott, W., and Hollender, J. (2005). Degradation of estradiol and ethinyl estradiol by activated sludge and by a defined mixed culture. *Appl Microbiol. Biotechnol.* 67, 106–112. doi: 10.1007/ s00253-004-1693-4

Xiong, W., Peng, W., and Liang, R. (2018). Identification and genome analysis of *Deinococcus actinosclerus* SJTR1, a novel 17β-estradiol degradation bacterium. *3 Biotech*. 8:433. doi: 10.1007/s13205-018-1466-x

Xiong, W., Yin, C., Peng, W., Deng, Z., Lin, S., and Liang, R. (2020). Characterization of an 17β-estradiol-degrading bacterium *Stenotrophomonas maltophilia* SJTL3 tolerant to adverse environmental factors. *Appl. Microbiol. Biotechnol.* 104, 1291–1305. doi: 10.1007/s00253-019-10281-8

Ye, X., Wang, H., Kan, J., Li, J., Huang, T., Xiong, G., et al. (2017). A novel 17β-hydroxysteroid dehydrogenase in *Rhodococcus* sp. P14 for transforming 17β-estradiol to estrone. *Chem. Biol. Interact.* 276, 105–112. doi: 10.1016/j.cbi. 2017.06.010

Yoshimoto, T., Nagai, F., Fujimoto, J., Watanabe, K., Mizukoshi, H., Makino, T., et al. (2004). Degradation of estrogens by *Rhodococcus zopfii* and *Rhodococcus equi* isolates from activated sludge in wastewater treatment plants. *Appl. Environ. Microbiol*. 70, 5283–5289. doi: 10.1128/AEM.70.9.5283-52 89.2004

Yu, C. P., Roh, H., and Chu, K. H. (2007). 17beta-estradiol-degrading bacteria isolated from activated sludge. *Environ. Sci. Technol.* 41, 486–492. doi: 10.1021/ es06092

Yu, C. P., Deeb, R. A., and Chu, K. H. (2013). Microbial degradation of steroidal estrogens. *Chemosphere* 91, 1225–1235. doi: 10.1016/j.chemosphere.2013. 01.112

Yu, Q., Wang, P., Liu, D., Gao, R., Shao, H., Zhao, H., et al. (2016). Degradation characteristics and metabolic pathway of 17β-estradiol (E2) by *Rhodococcus* sp. DS201. *Biotechnol. Bioprocess. Eng.* 21, 804–813. doi: 10.1007/s12257-016- 0283-5

Zeng, Q., Li, Y., Gu, G., Zhao, J., Zhang, C., and Luan, J. (2009). Sorption and biodegradation of 17β-estradiol by acclimated aerobic activated sludge and isolation of the bacterial strain. *Environ. Eng. Sci.* 26, 783–790. doi: 10.1089/ ees.2008.0116

Zhang, F.-F., Qin, D., Gao, L.-M., and Yu, C.-P. (2012b). Microbial degradation of estrogens in the environment. *Microbiol. China* 39, 711–721.

Zhang, T., Xiong, G., and Maser, E. (2011). Characterization of the steroid degrading bacterium S19-1 from the Baltic Sea at Kiel, Germany. *Chem. Biol. Interact.* 191, 83–88. doi: 10.1016/j.cbi.2010.12.021

Zhang, Y., Qin, F., Qiao, J., Li, G., Shen, C., Huang, T., et al. (2012a). Draft genome sequence of *Rhodococcus* sp. strain P14, a biodegrader of high-molecular-weight polycyclic aromatic hydrocarbons*. J. Bacteriol.* 194:3546. doi: 10.1128/ JB.00555-12

Zhao, H., Tian, K., Qiu, Q., Wang, Y., Zhang, H., Ma, S., et al. (2018). Genome analysis of *Rhodococcus* sp. strain DSSKP-R-001: a highly effective β-estradiol-degrading bacterium. *Int. J. Genomics* 2018:3505428. doi: 10.1155/ 2018/3505428

Zheng, D., Wang, X., Wang, P., Peng, W., Ji, N., and Liang, R. (2016). Genome sequence of *Pseudomonas citronellolis* SJTE-3, an estrogen- and polycyclic aromatic hydrocarbon-degrading bacterium. *Genome Announc.* 4:e01373-16. doi: 10.1128/genomeA.01373-16
